# SIRT2-mediated deacetylation of LCK governs the magnitude of T cell receptor signaling

**DOI:** 10.1038/s41590-025-02377-3

**Published:** 2026-01-29

**Authors:** Imene Hamaidi, Pingyan Cheng, Soo Young Jun, Alak Manna, Min-Hsuan Wang, Anh Nguyen, Ismail Can, Min G. Zhang, Odesha O. Taylor, Luis Uriel Lopez Bailon, Bin Fang, Bradford Perez, Ben C. Creelan, Andriy Marusyk, Dongjun Shin, Tae Hyun Hwang, Anders E. Berglund, Virginia S. Shapiro, Haitao M. Ji, José R. Conejo-Garcia, Sungjune Kim

**Affiliations:** 1 Department of Cancer Biology, Mayo Clinic, Jacksonville, FL USA; 2 Department of Immunology, Mayo Clinic, Jacksonville, FL USA; 3 TuHURA Biosciences Inc., Tampa, FL USA; 4 Department of Radiation Oncology, Mayo Clinic, Jacksonville, FL USA; 5 Taipei Veterans Generation Hospital, Taipei, Taiwan; 6 Division of Hematology, Mayo Clinic, Rochester, MN USA; 7 Department of Drug Discovery, H. Lee Moffitt Cancer Center, Tampa, FL USA; 8 Department of Integrative Immunobiology, Duke School of Medicine, Durham, NC USA; 9 Proteomics Core, H. Lee Moffitt Cancer Center, Tampa, FL USA; 10 Florida Cancer Specialists & Research Institute, Trinity Cancer Center, Trinity, FL USA; 11 Thoracic Oncology Program, H. Lee Moffitt Cancer Center, Tampa, FL USA; 12 Department of Tumor Biology, H. Lee Moffitt Cancer Center, Tampa, FL USA; 13 Tomocube Inc., San Diego, CA USA; 14 AI Research in Section of Surgical Sciences, Vanderbilt University, Nashville, TN USA; 15 Department of Quantitative Health Sciences, Mayo Clinic, Jacksonville, FL USA; 16 Department of Immunology, Mayo Clinic, Rochester, MN USA

**Keywords:** Signal transduction, Acetylation, T-cell receptor

## Abstract

T cell receptor (TCR) signaling is precisely tuned to prevent self-reactivity while allowing protective immunity. Here we found that acetylation modulated TCR signaling. The loss of SIRT2 deacetylase activity in T cells led to amplified calcium mobilization and phosphorylation of key proximal TCR molecules in naive T cells and reversed dampened TCR signaling in anergic T cells. During thymic selection, SIRT2 deficiency lowered the TCR signaling threshold and resulted in a broader TCR repertoire diversity. Mechanistically, we identified acetyl-lysine K228 on the linker region of LCK as a substrate specific for SIRT2 that governed LCK conformation and activity. SIRT2 inhibition in exhausted mouse and human tumor-infiltrating T cells restored TCR responsiveness and antitumor immunity. These findings highlighted SIRT2-modulated protein acetylation as a regulatory mechanism that set the TCR threshold in T cells.

## Abstract (web summary)

Phosphorylation-dephosphorylation and ubiquitination tune TCR signaling to avoid self-reactivity. Kim and colleagues show that acetylation also modulates TCR signaling.

## Main

T cell receptor (TCR) engagement with cognate peptide presented by major histocompatibility complex (MHC) initiates a cascade of intracellular signaling events crucial for T cell development and activation^1^. The strength of this interaction, influenced by co-stimulatory and co-inhibitory receptors, determines the balance between immune activation and tolerance. Thus, TCR signaling must be tightly regulated to ensure effective immunity while preventing autoimmunity.

Antigen recognition is transmitted through immunoreceptor tyrosine-based activation motifs (ITAMs) present on the TCR CD3γ/δ/ε/ζ chains^2^. These ITAMs are phosphorylated by Src family kinases, primarily LCK^3,4^, initiating recruitment of adaptor molecules and tyrosine kinases that activate phospholipase Cγ1 (PLCγ1). PLCγ1 catalyzes the generation of diacylglycerol (DAG) and inositol (1,4,5)-trisphosphate (IP3). IP3 induces calcium (Ca^2+^) release from the endoplasmic reticulum into the cytosol^5^, which activates calcineurin and promotes nuclear translocation of NFAT^6^. Concurrently, DAG recruits RasGRP1 and protein kinase C theta (PKCθ). The Ras activator RasGRP1 signals through ERK1/2 to promote formation of the AP-1 complex (JUN and FOS) that drives interleukin (IL)-2 (*Il2*) transcription^7,8^. Meanwhile, PKCθ promotes IKK degradation, resulting in the release and nuclear translocation of nuclear factor (NF)-κB^6^.

LCK activation represents a key step in proximal TCR signal transduction. Four conformational states of LCK have been described: closed and inactive (Y505-phosphorylated), primed but inactive (Y505-dephosphorylated), open and active (Y394-phosphorylated) and a dual-phosphorylated active form^9^. A pool of preactivated LCK is present at steady state, enabling rapid signal transduction upon TCR engagement. CD45-mediated dephosphorylation of the inhibitory residue Y505 primes LCK for autophosphorylation at Y394 (ref. ^4^). whereas the protein tyrosine kinase CSK re-establishes LCK inactivation by re-phosphorylating Y505 (ref. ^10^). Dephosphorylation of Y394 is regulated by a network of phosphatases, SHP-1, CD45, PTPN2 and PTPN22, which fine-tune LCK activity and prevent aberrant TCR signaling^4,11–14^.

Post-translational modifications of LCK, ZAP70 and LAT were shown to control the initiation, persistence and termination of TCR signaling. These modifications, including phosphorylation, ubiquitination and S-acylation have important roles in shaping both the magnitude and duration of TCR signaling^15–17^. Post-translational modifications allow rapid and reversible changes in protein activity to meet acute cellular demands^18^. Among these, lysine acetylation has emerged as a key regulatory mechanism across cellular pathways^19^, yet its role in proximal TCR signaling remains poorly understood. Sirtuins are NAD⁺-dependent deacetylases (SIRT1–7) with distinct subcellular localization and functions^20^. SIRT2, the only predominantly cytoplasmic member, regulates the cell cycle, metabolism and inflammation^21^. Here we found that SIRT2 modulated TCR signaling by deacetylating LCK.

## Results

### Loss of SIRT2 amplifies Ca^2+^ flux and TCR signaling

**Fig. 1:**
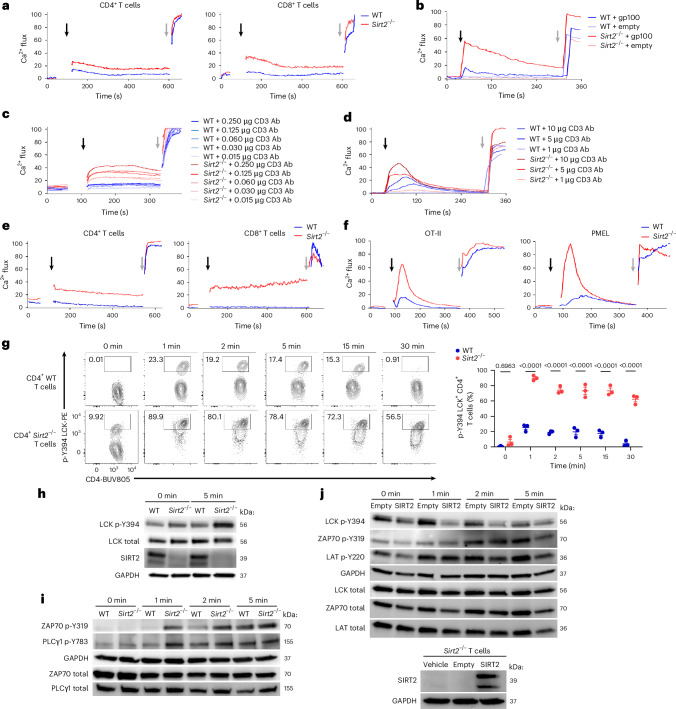
SIRT2 deficiency enhances Ca^2+^ flux and proximal TCR signaling in T cells. **a**, Ca^2+^ flux in naive CD4⁺ (left) and CD8⁺ (right) T cells isolated from the spleen of WT and *Sirt2*^−/−^ mice, loaded with Indo-1 AM and 0.5 µg ml^−1^ biotinylated CD3 Abs. Baseline fluorescence ratio was recorded for 60 s before simulation with 2.5 µg ml^−1^ streptavidin (black arrow). Ionomycin was added later (gray arrow) as a control of maximal Ca^2+^ release. *n* = 3 mice per group. **b**, Ca^2+^ flux in WT or *Sirt2*^−/−^ CD8^+^ PMEL T cells treated as in **a** and stimulated with gp100-loaded or empty EL4 cells. *n* = 3 mice per group. **c**, Ca^2+^ flux in WT or *Sirt2*^−/−^ CD4^+^ T cells treated as in **a** and stimulated with a concentration range (0.015, 0.03, 0.06, 0.125 and 0.25 µg ml^−1^) of biotinylated CD3 Abs. *n* = 3 mice per group. **d**, Ca^2+^ flux in WT or *Sirt2*^−/−^ CD4^+^ T cells treated as in **a** and stimulated with a concentration range (1, 5 and 10 µg ml^−1^) of biotinylated CD3 Abs. *n* = 3 mice per group. **e**, Ca^2+^ flux in preactivated WT or *Sirt2*^−/−^ CD4^+^ and CD8^+^ T cells prestimulated with 5 µg ml^−1^ plate-bound CD3 for 48 h, and then re-stimulated with 0.5 µg ml^−1^ biotinylated CD3 as in **a**. *n* = 3 mice per group. **f**, Ca^2+^ flux in WT or *Sirt2*^−/−^ CD4^+^ OT-II (left) and CD8^+^ PMEL (right) T cells cultured with IL-15 for 5 days following 72 h OVA or gp100 stimulation to generate T_M_-like cells, and then treated as in **a** with 2.5 µg ml^−1^ biotinylated CD3 Abs. *n* = 3 mice per group. **g**, Flow cytometry analysis of p-Y394 LCK (left) and frequency of p-Y394 LCK^+^ CD4^+^ T cells (right) before and after stimulation of WT or *Sirt2*^−/−^ naive CD4^+^ T cells with 0.5 µg ml^−1^ CD3 Ab for 0–30 min. Each dot represents one mouse (*n* = 3 mice per group). Data are mean ± s.e.m. **h**, Immunoblot analysis of p-Y394 and total LCK in WT and *Sirt2*^−/−^ naive CD3^+^ T cells before and after stimulation with plate-bound CD3 Abs for 0 min and 5 min. **i**, Immunoblot analysis of phosphorylated and total forms of ZAP70 and PLCγ1 in WT and *Sirt2*^−/−^ CD4^+^OT-II T cells preactivated with OVA peptide for 48 h, before and after stimulation with plate-bound CD3 Abs for 0, 1, 2 and 5 min. **j**, Immunoblot analysis of phosphorylated and total forms of LCK, ZAP70 and LAT in *Sirt2*^−/−^ T cells transduced with empty or SIRT2-expressing lentivector, before and after stimulation with plate-bound CD3 Abs for 0, 1, 2 and 5 min (top) and immunoblot of SIRT2 expression in *Sirt2*^−/−^ T cells transduced with vehicle, empty lentivector or SIRT2-expressing lentivector. In **h**–**j**, glyceraldehyde-3-phosphate dehydrogenase (GAPDH) levels were used as loading control. Data are representative of two (**b**–**e**,**j**) and three (**a**,**f**–**i**) independent experiments. Source data

**Extended Data Fig. 1:**
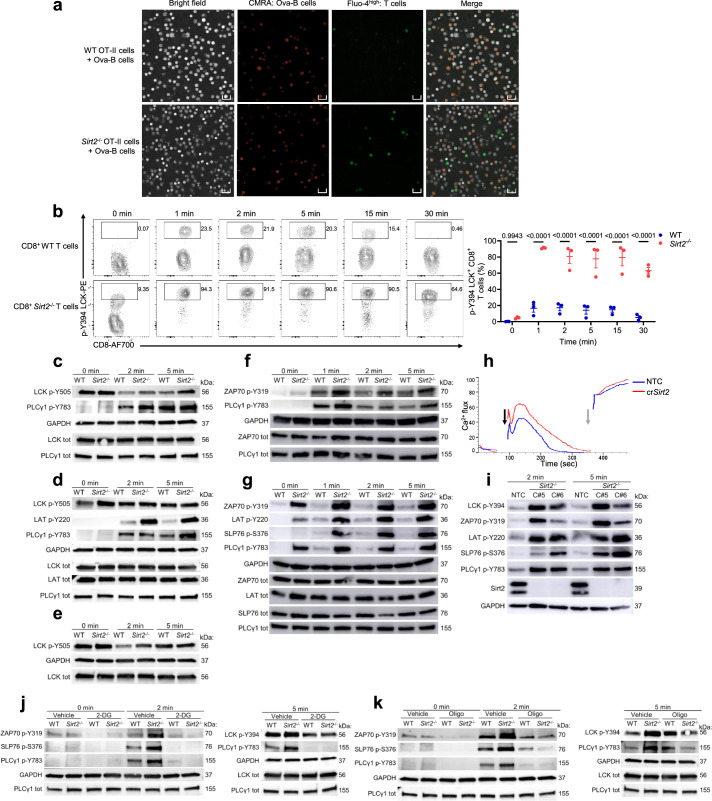
Sirt2 deficiency enhances calcium flux and proximal TCR signaling in T cells. **a**, Tomocube microscopy of WT and *Sirt2*^−/−^ OT-II T cells labeled with Fluo-4-AM (green) undergoing calcium flux after mixing with OVA-loaded B cells labeled with Cell Tracker Orange CMRA (orange). Overlays of fluorescent and bright-field images are shown. Scale bar, 15 µm. *n* = 5 mice per group. **b**, Flow-cytometric analysis of p-Y394 LCK in naive WT and *Sirt2*^−/−^ CD8⁺ T cells before and after stimulation with 0.5 µg ml^−1^ CD3 Abs for 0–30 min. Frequencies of p-Y394-LCK⁺ CD8⁺ T cells shown on right. Each dot = one mouse (*n* = 3 mice per group). Data = mean ± SEM. P values by two-way ANOVA. **c-e**, Immunoblot analysis of phosphorylated and total proximal TCR molecules before and after stimulation with 2.5 µg ml^−1^ CD3 Abs for 0–5 min in WT and *Sirt2*^−/−^ CD3^+^ (**c**), CD4⁺ OT-II (**d**) and CD8⁺ PMEL (**e**) T cells. **f,g**, Immunoblot analysis of phosphorylated and total proximal TCR molecules before and after stimulation with 2.5 µg ml^−1^ CD3 Abs for 0–5 min of WT and *Sirt2*^−/−^CD4^+^ OT-II (**f**) and CD8^+^ PMEL (**g**) T_M_-like cells generated by OVA and gp100 stimulation respectively followed by 5 days culture in IL-15. **h**, Calcium flux in Cas9–crRNA nontargeting control (NTC) and Cas9–crRNA SIRT2-targeted (cr*Sirt2*) Jurkat cells loaded with Indo-1 AM and labeled with 0.5 µg ml^−1^ biotinylated CD3 Abs. Baseline fluorescence recorded for 60 s before stimulation with 2.5 µg ml^−1^ streptavidin (black arrow) and ionomycin (gray arrow). *n* = 3 independent clones. **i**, Immunoblot analysis of phosphorylated and total proximal TCR molecules after stimulation with 2.5 µg ml^−1^ CD3 Abs for 2 and 5 min in NTC and cr*Sirt2* Jurkat clone no. 5 and no. 6. **j,k**, Immunoblot analysis of phosphorylated and total proximal TCR signaling molecules in CD3⁺ T cells from WT and *Sirt2*^−/−^ mice treated with either the glycolysis inhibitor 2-deoxy-D-glucose (2-DG, 1 mM) (**j**) or the oxidative-phosphorylation inhibitor oligomycin (100 nM) (**k**) for 24 h, followed by stimulation with 2.5 µg ml^−1^ CD3 Abs for 0–5 min. **c-g, i-k**, GAPDH was used as loading control. Data are representative of one (**a**) two (**j,k**) and three (**b-i**) independent experiments. Source data

Because intracellular Ca^2+^ flux represents the pivotal event during TCR signaling, we characterized the impact of SIRT2 deficiency on Ca^2+^ mobilization following TCR ligation, which occurs within seconds after TCR engagement, using Indo-1, a Ca^2+^ fluorescent ratiometric dye. Markedly elevated Ca^2+^ flux was observed in *Sirt2*^−/−^ T cells freshly isolated from spleen or lymph nodes (LNs) compared to wild-type (WT) T cells upon TCR ligation with CD3 antibodies (Abs) (Fig. 1a). Consistent with enhanced TCR sensitivity, physiological antigenic stimulation with gp100-loaded EL4 mouse thymoma cells (used as antigen-presenting cells) triggered stronger Ca^2+^ mobilization in *Sirt2*^−/−^ gp100-specific CD8⁺ TCR transgenic PMEL T cells compared to WT T cells (Fig. 1b). No Ca²⁺ flux was detected in response to unloaded EL4 cells (Fig. 1b), confirming the antigen-specific response by PMEL T cells. Enhanced Ca²⁺ signaling in SIRT2-deficient T cells was further validated using live-cell imaging, where *Sirt2*^−/−^ OVA-specific CD4⁺ TCR transgenic OT-II T cells exhibited greater Ca²⁺ flux than their WT counterparts following stimulation with OVA-loaded B cells (Extended Data Fig. 1a).

To determine whether the amplified TCR signaling observed in *Sirt2*^−/−^ T cells reflected a lowered activation threshold, we measured Ca²⁺ mobilization in response to varying doses of CD3 Ab stimulation. To probe subtle differences in TCR signaling thresholds, we first used suboptimal doses of CD3 Abs to mimic the weak antigenic stimulation within the tumor microenvironment. *Sirt2*^−/−^ T cells exhibited increased Ca²⁺ flux compared to WT T cells, even at low (0.015–0.250 µg ml^−1^) CD3 Ab concentrations (Fig. 1c), indicating enhanced sensitivity to TCR engagement. Similarly, when using a broader CD3 Ab range (1–10 µg ml^−1^), *Sirt2*^−/−^ T cells consistently displayed stronger TCR responses than WT T cells (Fig. 1d).

In anergic T cells, there is a profound reduction in Ca^2+^ mobilization upon restimulation^16,22^. Consistently, WT T cells cultured in vitro for 48 h with CD3 antibodies in the absence of CD28 co-stimulation (anergic conditions), exhibited a complete decoupling of proximal TCR signaling, as evidenced by minimal Ca^2+^ mobilization upon restimulation (Fig. 1e). *Sirt2*^−/−^ T cells cultured in the same conditions preserved their capacity for Ca^2+^ mobilization upon restimulation (Fig. 1e). Additionally, Ca^2+^ mobilization was significantly increased in IL-15-differentiated *Sirt2*^−/−^ CD4^+^ OT-II or CD8^+^ PMEL memory-like T (T_M_-like) cells compared to WT counterparts (Fig. 1f).

Next, we investigated the cascade of phosphorylation events upstream of Ca^2+^ mobilization that mediate proximal TCR signaling. Consistent with increased Ca^2+^ mobilization upon TCR ligation, higher amounts of the active form of phospho-LCK (p-Y394) was apparent in *Sirt2*^−/−^ T cells (Fig. 1g,h and Extended Data Fig. 1d). While phosphorylation at the inhibitory tyrosine Y505 (p-Y505) decreased upon TCR stimulation in both WT and *Sirt2*^−/−^ T cells, *Sirt2*^−/−^ T cells retained higher p-Y505 compared to WT T cells at both baseline and after activation (Extended Data Fig. 1c–e), reflecting the elicitation of compensatory inhibitory feedback mechanism. Similarly, enhanced phosphorylation of proximal TCR signaling molecules including the protein kinase ZAP70 and PLCγ1 was observed in preactivated *Sirt2*^−/−^ T cells, as well as in IL-15-differentiated CD4^+^ OT-II and CD8^+^ *Sirt2*^−/−^ PMEL T_M_-like cells (Fig. 1i and Extended Data Fig. 1f,g). Increased Ca^2+^ mobilization and proximal TCR complex phosphorylation were also observed in CRISPR *Sirt2*^−/−^ human Jurkat cells (Extended Data Fig. 1h,i). Re-introduction of SIRT2 in *Sirt2*^−/−^ T cells reduced phosphorylation of LCK, as well as that of the LCK-downstream targets ZAP70 and adaptor LAT following TCR ligation (Fig. 1j), confirming that SIRT2 directly impacted proximal TCR signaling.

SIRT2 negatively regulates the activity of key metabolic enzymes such as hexokinase 1, phosphofructokinase P, enolase-1 and lactate dehydrogenase^21^ and its inhibition promotes metabolic resilience and supports the bioenergetic fitness required for effective T cell responses^21^. To test whether the enhanced TCR signaling observed in *Sirt2*^−/−^ T cells could be attributed, at least in part, to altered metabolic activity, we pretreated WT and *Sirt2*^−/−^ T cells with the glycolysis inhibitor 2-deoxy-D-glucose (2-DG) or the mitochondrial ATP synthase inhibitor oligomycin before TCR stimulation. As expected, metabolic inhibition globally suppressed proximal TCR signaling in both *Sirt2*^−/−^ and WT T cells (Extended Data Fig. 1j,k), but WT T cells exhibited slightly stronger proximal signaling than *Sirt2*^−/−^ T cells (Extended Data Fig. 1j,k), suggesting that the enhanced TCR signaling in *Sirt2*^−/−^ T cells might be partially driven by their elevated metabolic activity. These results indicated that SIRT2 deficiency amplified TCR signaling and reduced the activation threshold throughout T cell ontology, whereas SIRT2 deficiency reversed dampened TCR signaling in anergic T cells.

### SIRT2 binds and deacetylates LCK to regulate its activity

**Fig. 2:**
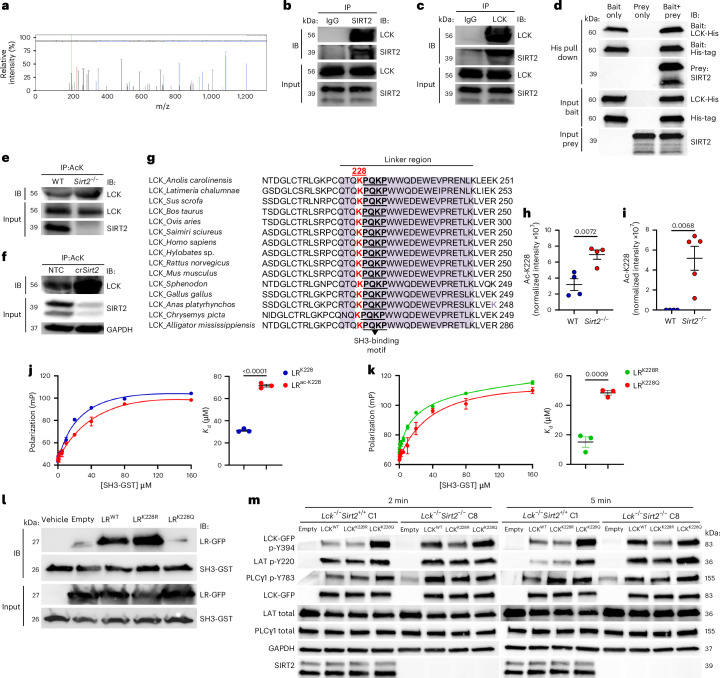
IP–MS/MS analysis identifies K228 as a SIRT2-regulated acetylation site that controls LCK kinase activity. **a**, Collision-induced dissociation spectra of the LCK_ILEQSGEWWK peptide detected by LC–MS/MS following SIRT2 immunoprecipitation from mouse CD3⁺ T cell lysates. **b**,**c**, Immunoblot analysis of SIRT2 and LCK after co-IP of LCK with SIRT2 Abs (**b**) and reverse co-IP of SIRT2 with LCK Abs (**c**) from cell lysates of mouse activated CD3^+^ T cells. Inputs show total protein controls. **d**, Immunoblot analysis of His-tag, LCK-His and SIRT2 following pulldown assay of purified His-tagged human LCK (bait) bound to cobalt resin and incubated with vehicle (lane 1) or purified human SIRT2 (prey) (lane 3). Cobalt resin incubated with vehicle (no bait) followed by addition of SIRT2 served as control (lane 2). Equal protein inputs are shown. **e**,**f**, Acetylation of LCK detected by immunoblot analysis of LCK following acetyl-lysine immunoprecipitation from WT and *Sirt2*^−/−^ activated mouse CD3⁺ T cells (**e**) or NTC (Cas9–crRNA nontargeting control) and cr*Sirt2* (Cas9–crRNA targeting SIRT2) (**f**). LCK or GAPDH blots from total lysates serve as loading controls. **g**, Sequence alignment of LCK orthologs showing conservation of the LR (purple) and K228 (red font) across species; the underlined PQKP sequence indicates an SH3-binding motif. **h**,**i**, Quantification of LCKK228 acetylation in WT and *Sirt2*^−/−^ preactivated CD3⁺ T cells (*n* = 4) (**h**) and in WT (*n* = 4) and *Sirt2*^−/−^ (*n* = 5) CD4⁺ OT-II T_M_-like cells (**i**) by LC–MS/MS following LCK IP. Acetylated peptide intensity was normalized to total peptide intensity. Each dot represents one mouse. **j**,**k**, FP binding assays of either unmodified LR^K228^ or LR^ac-K228^ peptides (**j**), and either LR^K228R^ or LR^K228Q^ mutants (**k**), incubated with increasing concentrations (0–160 µM) of SH3-GST protein, showing representative binding curves (left) and mean dissociation constants (*K*_d_) (right): LR^K228^ = 30 ± 1.5 µM; LR^ac-K228^ = 73 ± 2.2 µM; LR^K228R^ = 18 ± 0.7 µM; and LR^K228Q^ = 48 ± 2.6 µM. *n* = 3 independent experiments. **l**, Pulldown assay of GFP-tagged LR^WT^, LR^K228R^ or LR^K228Q^ using GST-fused SH3 immobilized on glutathione resin, followed by immunoblotting with GFP and GST antibodies. Controls include vehicle and empty vector. Equal inputs are shown. **m**, Immunoblots showing phosphorylated and total proximal LCK, LAT and PLCγ1 after stimulation with 2.5 µg ml^−1^ plate-bound CD3 Abs for 2 min and 5 min in *Lck*^−/−^*Sirt2*^+/+^ (clone C1) and *Lck*^−/−^*Sirt2*^−/−^ (clone C8) Jurkat cell clones reconstituted with empty, LCK^WT^, LCK^K228R^ or LCK^K228Q^ vectors. GAPDH was used as a loading control. Data are mean ± s.e.m. *P* values are determined by two-tailed Student’s *t*-test (**h**–**k**). Data are representative of at least two (**a**–**g**,**l**) and three (**h**–**k**,**m**) independent experiments. Source data

To gain insight into the molecular mechanisms underlying amplified TCR signaling with SIRT2 deficiency, we analyzed the SIRT2 interactome in T cells using a published dataset (PXD012811)^21^. Mass spectrometry (MS) analysis revealed interactions between SIRT2 and several proximal TCR effector molecules, most prominently LCK (Fig. 2a), so we focused on characterizing the interaction between SIRT2 and LCK. Binding between SIRT2 and LCK was detected through co-immunoprecipitation (IP) and reverse co-IP followed by immunoblot assays on CD3^+^ T cell lysates (Fig. 2b,c). Direct binding was tested using purified SIRT2 and LCK-His proteins (Fig. 2d). Analysis of the acetylation levels of LCK in WT versus *Sirt2*^−/−^ T cells by IP with acetyl-Lys (acK) Abs, followed by immunoblot for LCK indicated increased acetylation of LCK in *Sirt2*^−/−^ versus WT CD3^+^ T cells and Jurkat cells (Fig. 2e,f), suggesting that LCK was a deacetylase target of SIRT2.

**Extended Data Fig. 2:**
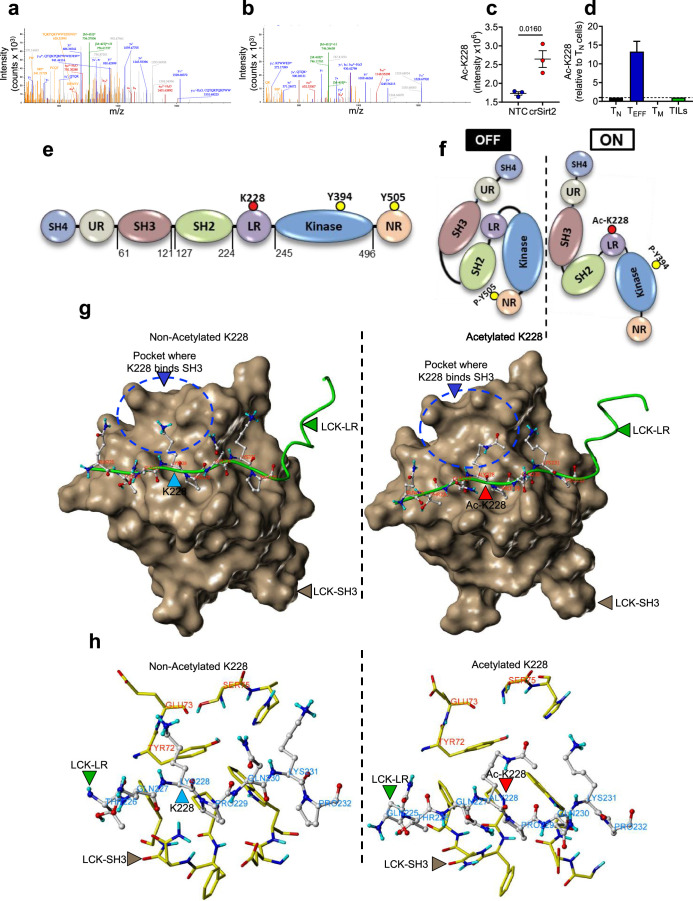
IP-MS/MS analysis identifies K228 as an acetylation site modulating LCK conformation. **a,b**, CID spectra of LSRPCQTQKPQKPWWEDEWEVPR peptide derived from LCK carrying acetylation on K228 identified by LC–MS/MS from LCK-immunoprecipitated primary mouse T cell lysates (**a**) and human Jurkat cell lysates (**b**). **c**, Quantification of LCKK228 acetylation in NTC (Cas9–crRNA non-targeting control) and crSIRT2 (Cas9–crRNA targeting SIRT2) Jurkat cell lysates by LC–MS/MS following LCK immunoprecipitation. Acetylated peptide intensity was normalized to total peptide intensity. *n* = 3 biological replicates. Data are mean ± SEM. P values by two-tailed Student’s t-test. **d**, Quantification of LCKK228 acetylation in freshly isolated T cells (T_N_), OVA-activated OT-II T cells (T_EFF_-like), IL-15–differentiated OT-II T_M_-like cells and mouse TILs isolated from B16F10 subcutaneous by LC–MS/MS following LCK immunoprecipitation. Intensities of acetylated peptides shown relative to T_N_ cells. T_N_, *n* = 1 (pooled from 10 mice); T_EFF_, *n* = 4 independent mice; T_M_, *n* = 4 independent mice; TILs, *n* = 1 (pooled from 10 mice). **e**, Schematic of LCK domains. Src homology 4 (SH4), unique region (UR), SH3, SH2, linker region (LR), kinase domain and negative-regulatory tail (NR) are depicted. **f**, Model of LCK conformational control by intramolecular interactions. Left, inactive closed form stabilized by: (i) phospho-Y505 (on the NR) binding to the SH2 domain and (ii) LR–SH3 interaction. Right, active open form achieved through: (i) Y505 dephosphorylation releasing the NR from SH2 and (ii) LR disengagement from SH3. **g,h**, Predicted binding mode of the LCK linker region to the SH3 domain modeled using AlphaFold3 and visualized in PyMOL. Left panels depict the non-acetylated form; right panels show the structural rearrangement upon K228 acetylation, which disrupts charge interaction and excludes the side chain from the pocket. g, Surface representation showing a shallow pocket on the SH3 domain (circled in blue) predicted to accommodate K228. The sequence of the LCK linker region (residues Q225–P232) was labeled. h, Stick model of the same complex showing the linker region (Q225–P232) and the three SH3 domain residues Y72, E73 and S75 defining the pocket predicted to accommodate K228. Data are representative of one (**d**), three (**b,c**) and four (**a**) independent experiments. Source data

We next conducted an in-depth profiling of LCK post-translational modifications. Immunoprecipitation followed by LC–MS/MS analysis of LCK from WT and *Sirt2*^−*/*−^ primary mouse CD3⁺ T cells and human Jurkat cells revealed a unique acetylation site at lysine 228 (K228) within the linker region (LR) of LCK (Extended Data Fig. 2a,b). This residue was highly conserved in mouse and human T cells and is adjacent to a known SH3-binding motif (PQKP)^23^ (Fig. 2g). We found increased acetylation levels of K228 in preactivated *Sirt2*^−/−^CD3^+^ T cells, IL-15-differentiated CD4^+^ OT-II T_M_-like cells and Jurkat cells compared to their WT counterparts (Fig. 2h,i and Extended Data Fig. 2c). Notably, differential K228 acetylation were observed among T cell subsets, with the highest levels in effector-like (T_EFF_-like) CD44^+^CD62L^−^ T cells and the lowest in IL-15 differentiated CD44^+^CD62L^+^ T_M_-like cells, followed by B16F10 tumor-infiltrating lymphocytes freshly isolated from subcutaneous (s.c.) tumors and unstimulated CD44^−^CD62L^+^ naive T (T_N_) cells (Extended Data Fig. 2d).

**Extended Data Fig. 3:**
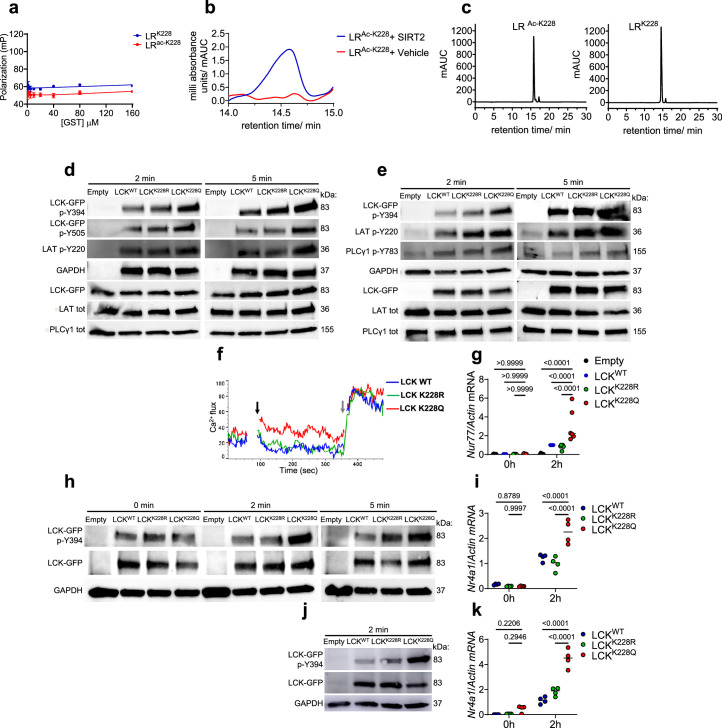
Lysine K228 is a SIRT2-regulated acetylation site controlling LCK kinase activity. **a**, Negative controls for fluorescence polarization assay of either unmodified LR^K228^ or LR^ac-K228^ peptides), incubated with increasing concentrations (0–160 µM) of SH3-GST protein showing representative binding curves *n* = 3 independent experiments. **b**, HPLC trace of LR^ac-K228^ peptide incubated with vehicle or purified SIRT2 protein as described in the Methods. Peak corresponds to deacetylated LR^K228^. **c**, HPLC traces of standard acetylated LR^ac-K228^ (left) and deacetylated LR^K228^ (right) peptides with retention times of 15.797 min and 14.553 min, respectively. **d,e**, Immunoblots showing phosphorylated and total proximal TCR signaling molecules after stimulation with 2.5 µg/ml plate-bound CD3 Abs for 2 min and 5 min in two independent *Lck*^−/−^ Jurkat clones reconstituted with empty, LCK^WT^, LCK^K228R^ or LCK^K228Q^ vectors. **f**, Calcium flux in *Lck*^−/−^ Jurkat cells reconstituted with LCK^WT^, LCK^K228R^ or LCK^K228Q^ vectors loaded with Indo-1 AM and labeled with 2.5 µg/ml biotinylated CD3 Abs. Indo-1 ratios indicate baseline and post-stimulation fluorescence following 2.5 µg/ml streptavidin (black arrow) and ionomycin (gray arrow). **h,j**, Immunoblots of p-Y394 and total LCK after stimulation with 2.5 µg/ml plate-bound CD3 Abs, in mouse (**h**) and human (**j**) *Lck*^−/−^ CD3⁺ T cells reconstituted with empty, LCK^WT^, LCK^K228R^ or LCK^K228Q^ vectors. **g,i,k**, Relative *Nr4a1* mRNA expression quantified by qRT-PCR in resting T cells and 2 h post-stimulation with 2.5 µg/ml plate-bound CD3 Abs in *Lck*^−/−^ Jurkat cells (**g**; *n* = 6 independent clones), mouse CD3⁺ T cells (**i**; *n* = 4 technical replicates from 5 pooled mice) and human CD3⁺ T cells (**k**; *n* = 4 technical replicates). Data are mean ± SEM. *P* values are determined by two-way ANOVA (**g**,**i,k**). Data are representative of two (**b,c**) and three (**a,d-k**) independent experiments. Source data

LCK activity is controlled by intramolecular interactions that regulate its conformational state. In resting T cells, LCK predominantly adopts an inactive closed conformation, which is stabilized by two intramolecular inhibitory interactions: binding of the phosphorylated inhibitory Y505 residue on the negative regulatory (NR) domain to the Src homology 2 (SH2) domain, and binding of the LR to the SH3 domain^24^ (Extended Data Fig. 2e,f). To explore whether acetylation of K228 altered SH3–LR interaction, we performed AlphaFold3-based structural docking analysis^25^, which predicted that K228 is accommodated in a shallow surface pocket of the SH3 domain, flanked by Tyr72, Glu73 and Ser75 (Extended Data Fig. 2g,h) In this configuration, unacetylated K228 forms favorable electrostatic interactions with Glu73 in the SH3 domain, stabilizing the closed conformation (Extended Data Fig. 2g,h). In contrast, K228 acetylation disrupts this electrostatic interaction, favoring an open, primed LCK state (Extended Data Fig. 2g,h). These structural predictions were validated by fluorescence polarization (FP) assays, showing that LR peptides with acetylated K228 (LR^Ac-K228^) had significantly lower binding affinity for the SH3-gluthatione-S-transferase (GST) domain (*K*_d_ = 71 ± 2.2 μM) than non-acetylated peptides (LR^K228^) (*K*_d_ = 31 ± 1.5 μM) (Fig. 2j). As a negative control, the GST protein alone showed no binding activity to either peptide (Extended Data Fig. 3a).

Next, we used the K228Q and K228R mutants of LR (LR^K228Q^ and LR^K228R^) to mimic the acetylated and non-acetylated states of LCK, respectively. FP assays demonstrated that the LR^K228R^ peptide bound more tightly to the SH3 domain than the LR^K228Q^ peptide (Fig. 2k). Furthermore, GST pulldown assays followed by immunoblotting confirmed that SH3-GST preferentially bound LR^WT^ and LR^K228R^ over LR^K228Q^ (Fig. 2l). An HPLC-based assay using recombinant SIRT2 and LR^Ac-K228^ peptides showed efficient deacetylation of LR^Ac-K228^ by SIRT2 (Extended Data Fig. 3b,c), indicating acetyl-K228 LCK was a direct substrate of SIRT2.

**Extended Data Fig. 4:**
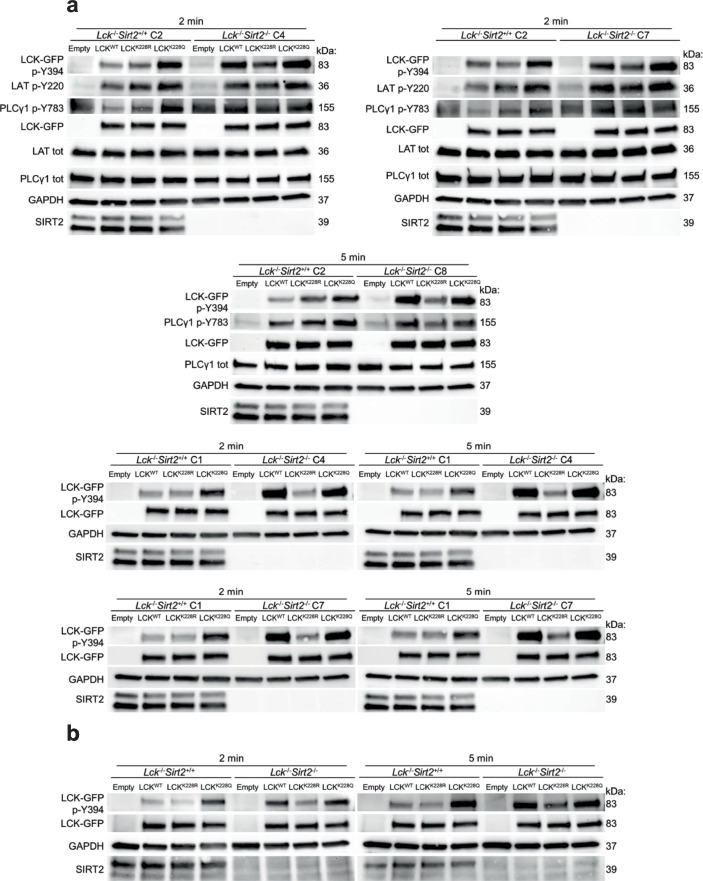
Loss of SIRT2 enhances LCK activation and downstream TCR signaling through LCKK228 acetylation. **a**, Immunoblot analysis of phosphorylated and total LCK, LAT and PLCγ1 following TCR stimulation with 2.5 µg/ml CD3 Abs for 2–5 min in CRISPR-Cas9–generated LCK-deficient Jurkat clones (*Lck*^−/−^*Sirt2*^+/+^, C1 and C2) and double LCK–SIRT2-deficient Jurkat clones (*Lck*^−/−^*Sirt2*^−/−^, C4, C7 and C8) reconstituted with empty, LCK^WT^, LCK^K228R^ or LCK^K228Q^ vectors. **b**, Immunoblot analysis of phosphorylated and total LCK following stimulation with 2.5 µg/ml CD3 Abs for 2–5 min in LCK-deficient primary mouse CD3^+^ T cells in WT or *Sirt2*^−/−^ backgrounds reconstituted with empty, LCK^WT^, LCK^K228R^ or LCK^K228Q^ vectors. GAPDH used as loading control. Data representative of three independent experiments. Source data

To understand the functional relevance of K228 deacetylation by SIRT2 on LCK kinase activity, *Lck*^−/−^ Jurkat clones, primary mouse CD3^+^ T cells and human CD3^+^ T cells from healthy donors were reconstituted with an empty vector, LCK^WT^ or LCK^K228R^ and LCK^K228Q^ mutants. The acetylation-mimetic LCK^K228Q^ consistently promoted increased LCK phosphorylation, Ca²⁺ mobilization and transcription of *Nr4a1*, an early transcriptional target downstream of TCR signaling (Extended Data Fig. 3d–k), upon TCR ligation compared to LCK^WT^. In contrast, the deacetylation-mimetic LCK^K228R^ induced LCK phosphorylation, Ca²⁺ mobilization and *Nr4a1* transcription levels comparable to those induced with LCK^WT^ following TCR ligation (Extended Data Fig. 3d–k). To further define the direct contribution of SIRT2-mediated deacetylation of LCK at K228 to the regulation of TCR signaling, we generated *Lck*^−/−^ and *Lck*^−/−^*Sirt2*^−/−^ Jurkat clones and reconstituted them with LCK^WT^, LCK^K228R^ or LCK^K228Q^. Reconstituted LCK^WT^ displayed markedly increased phosphorylation following TCR stimulation in *Lck*^−/−^*Sirt2*^−/−^ Jurkat cell compared to the *Lck*^−/−^*Sirt2*^+/+^ Jurkat cells (Fig. 2m and Extended Data Fig. 4a), consistent with enhanced LCK^K228^ acetylation and LCK hyperactivation in *Sirt2*^−/−^ cells. In contrast, reconstituted LCK^K228R^ remained hypophosphorylated in both *Lck*^−/−^*Sirt2*^+/+^ and *Lck*^−/−^*Sirt2*^−/−^ Jurkat cells, whereas reconstituted LCK^K228Q^ exhibited robust phosphorylation in both *Lck*^−/−^*Sirt2*^+/+^ and *Lck*^−/−^*Sirt2*^−/−^ Jurkat cells (Fig. 2m and Extended Data Fig. 4a). LCK activation closely correlated with phosphorylation of downstream TCR signaling intermediates, including LAT and PLC-γ1 (Fig. 2m and Extended Data Fig. 4a), further supporting a role for K228 acetylation in tuning proximal TCR signaling. Similar results were observed in LCK-deficient primary mouse T cells (Extended Data Fig. 4b). These structural, biochemical and functional data indicated that SIRT2 directly reduced LCK activity by deacetylating K228.

### SIRT2 deficiency augments downstream TCR signaling

**Fig. 3:**
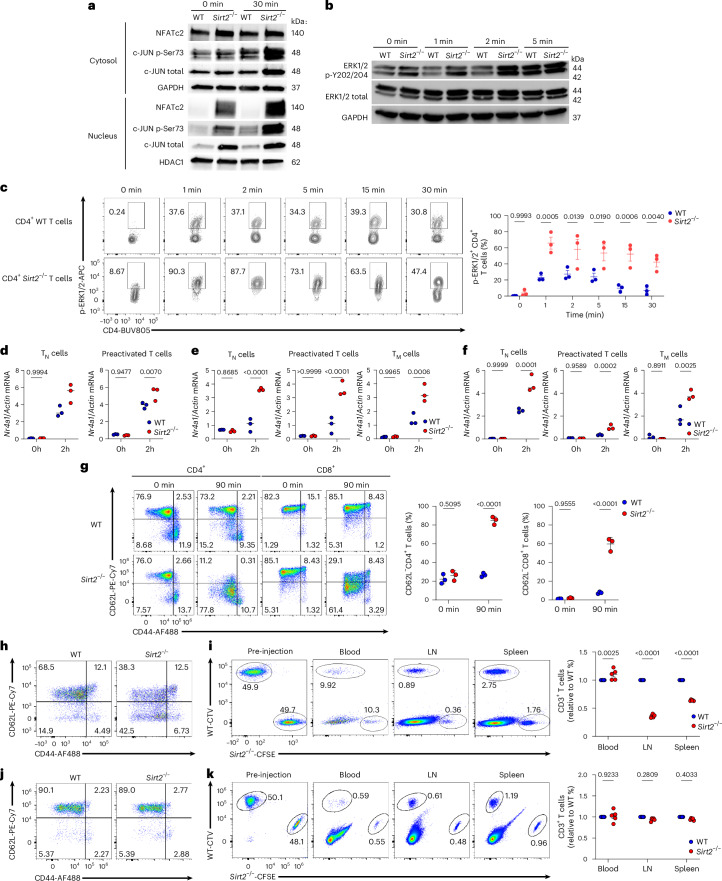
SIRT2 deficiency augments downstream TCR signaling in T cells. **a**, Immunoblots of NFATc2, phospho-S73 and total c-JUN in cytosolic and nuclear fractions of WT and *Sirt2*^−/−^ mouse naive CD3⁺ T cells before and after stimulation with 5 µg ml^−1^ plate-bound CD3 for 30 min. GAPDH and HDAC1 serve as cytosolic and nuclear loading controls, respectively. **b**, Immunoblot of p-Y202/204 and total ERK1/2 in WT and *Sirt2*^−/−^ naive CD3⁺ T cells before and after stimulation with 5 µg ml^−1^ plate-bound CD3 Abs for 0, 1, 2 and 5 min. GAPDH was used as loading control. **c**, Representative flow cytometry analysis of p-ERK1/2 (left) and frequencies of p-ERK1/2^+^ CD4 ^+^T cells (left) before and after stimulation with 0.5 µg ml^−1^ CD3 Ab for 0, 1, 2, 5, 15 and 30 min, in WT *or Sirt2*^−/−^ naive CD4^+^ T cells. Each dot represents one mouse (*n* = 3 mice per group). **d**–**f**, Relative *Nr4a1* mRNA expression in WT and *Sirt2*^−/−^ CD3⁺ T cells freshly isolated (T_N_ cells) or preactivated for 48 h with 5 µg ml^−1^ plate-bound CD3 (preactivated T cells) after isolation from the spleen of C57BL/6 mice (**d**), CD4⁺ T cells freshly isolated (T_N_ cells), preactivated for 48 h with 10 µg ml^−1^ OVA peptide (preactivated T cells) or differentiated into T_M_-like cells by 5 days of culture with IL-15 (T_M_ cells) after isolation from the spleen of OT-II mice (**e**), or CD8⁺ T cells freshly isolated (T_N_ cells), preactivated for 48 h with 1 µg ml^−1^ gp100 peptide (preactivated T cells) or differentiated by 5 days of culture with IL-15 (T_M_ cells) after isolation from the spleen of PMEL mice (**f**) and analyzed by RT–qPCR before (0 h) and after (2 h) stimulation with 5 µg ml^−1^ plate-bound CD3 Abs for 2 h. Each dot represents one mouse (*n* = 3 mice per group). **g**, Representative flow cytometry analysis of CD62L expression (left) and frequencies of CD62L^−^ T cell subsets (right) in CD4⁺ and CD8⁺ T cells from WT and *Sirt2*^−/−^ mice before and after stimulation with 0.25 µg ml^−1^ CD3 Abs for 90 min. Each dot reprersents one mouse (*n* = 3 mice per group). **h**,**j**, Representative flow cytometry analysis of CD62L expression in CFSE-labeled *Sirt2*^−/−^ and CTV-labeled WT T cells preactivated with 0.5 µg ml^−1^ CD3 Abs for 1 h (**h**) or left unstimulated (**j**) before injection into C57BL/6 recipient mice. **i**,**k**, Representative flow cytometry analysis (right) and frequencies normalized to WT T cells within each mouse (right) of CFSE⁺ *Sirt2*^−/−^ and CTV⁺ WT T cells preactivated as in **h** (**i**) or left unstimulated (**k**) before injection and in the blood, LNs and spleens of recipient mice 1 h after co-transfer transfer (1:1) into C57BL/6 recipient mice. Each dot represents one recipient mouse (*n* = 5). Data are mean ± s.e.m. *P* values were determined by two-way analysis of variance (ANOVA) (**c**–**g**,**i**,**k**). Data are representative of at least two (**a**,**b**) and three (**c**–**k**) independent experiments. Source data

**Extended Data Fig. 5:**
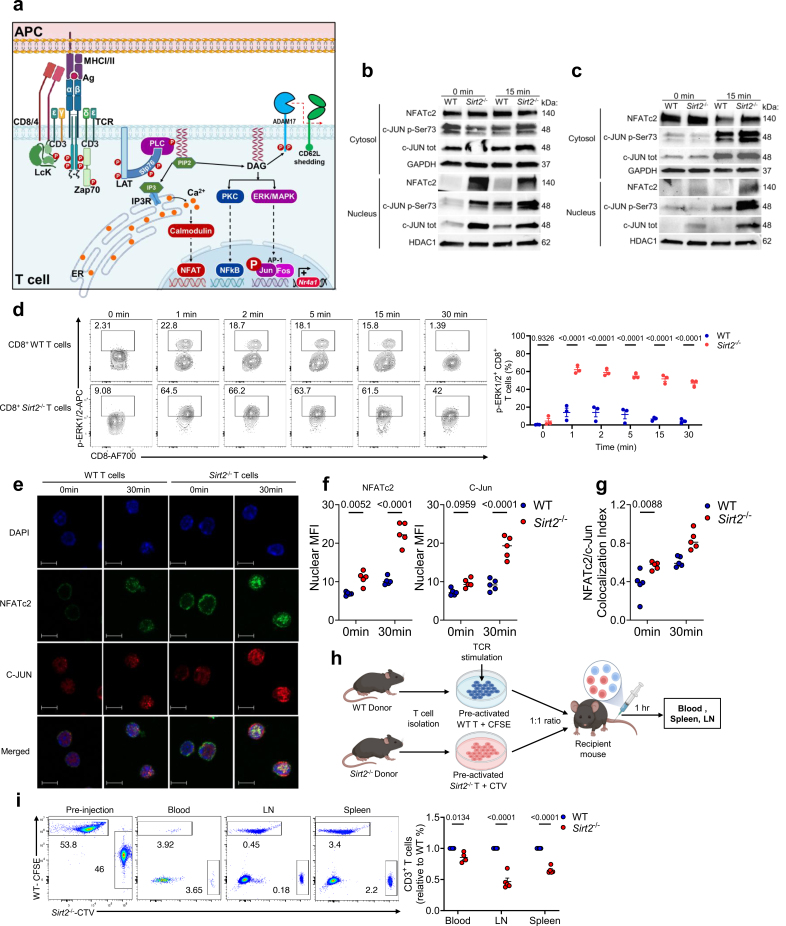
Sirt2 deficiency augments downstream TCR signaling in T cells. **a**, Schematic of downstream TCR signaling. Antigen recognition by TCR triggers recruitment of protein tyrosine kinases and adaptor molecules, leading to PLCγ1 activation. PLCγ1 catalyzes DAG and IP₃ production. IP₃ induces Ca²⁺ release promoting NFAT nuclear translocation. DAG recruits RasGRP1 and PKCθ. RasGRP1 signals through ERK1/2, leading to AP-1 activation, while PKCθ promotes NF-κB activation. Nr4a1 is an early-gene upregulated post-TCR activation, and CD62L is downregulated via ectodomain shedding by ADAM17. Created using BioRender.com. **b,c**, Immunoblots of NFATc2, phospho-S73 and total c-JUN in cytosolic and nuclear fractions before and after stimulation with 5 µg/ml CD3 Abs for 30 min in WT and *Sirt2*^−/−^ CD4^+^ OT-II cells pre-stimulated with OVA peptide for 48 h (**b**) or cultured for additional 5 days with IL-15 (**c**). GAPDH and HDAC1 serve as cytosolic and nuclear loading controls, respectively. **d**, Flow cytometry plots (left) and frequencies of p-ERK1/2^+^CD8^+^ WT and *Sirt2*^−/−^ T cells (left) before and after stimulation with 0.5 µg/ml CD3 Ab for 0, 1, 2, 5, 15 and 30 min. Each dot = one mouse (n = 3 mice per group). **e–g**, Confocal microscopy of NFATc2 and c-JUN localization in WT and *Sirt2*^−/−^ CD3^+^ T cells before and 30 min after stimulation. e, Representative images showing DAPI (blue), NFATc2 (Alexa 488; red) and c-JUN (Alexa 647; green) and overlays fluorescence. Scale bar, 10 µm. f, Nuclear NFATc2 and c-JUN mean fluorescence intensity (MFI) before and after stimulation. **g**, Colocalization index of nuclear NFATc2 and c-JUN before and after s stimulation (r; –1, no colocalization; +1, perfect colocalization). *n* = 5. **h**, Experimental design of CTV-labeled *Sirt2*^−/−^ T cells and CFSE-labeled WT T cells stimulated with CD3 Abs, mixed at a 1:1 ratio and transferred into C57BL/6 mice. Created using BioRender.com. **i**, Fow-cytometric plots (right) and frequencies of pre-activated CFSE⁺ WT and CTV⁺ *Sirt2*^−/−^ T cells normalized to WT T cells within each mouse (right) before injection and 1 h after co-transfer (1:1) in blood, lymph nodes (LN) and spleens of recipient mice. Each dot = one mouse (n = 5). Data are mean ± SEM. *P* values are determined by two-way ANOVA (**d,f,g,i**). Data are representative of two (**b,c,e,f,g**) and three (**d,i**) independent experiments. Source data

To investigate the downstream consequences of an amplified proximal TCR signaling in *Sirt2*^−/−^ T cells, we assessed activation of the NFAT and ERK–MAPK–AP-1 signaling axes, induction of *Nr4a1* transcription and L-selectin (CD62L) shedding in *Sirt2*^−/−^ and WT T cells following TCR stimulation (Extended Data Fig. 5a). Nuclear translocation of NFATc2 (Fig. 3a and Extended Data Fig. 5b,c) phospho-ERK1/2 (Fig. 3b,c and Extended Data Fig. 5d) and phosphorylation and nuclear translocation of c-Jun (Fig. 3a and Extended Data Fig. 5b,c) were increased in *Sirt2*^−/−^ T cells compared to WT T cells upon TCR ligation, suggesting robust TCR signaling.

NFAT cooperates with Fos-Jun to induce the transcription of cytokine and activation-associated genes^26^. When unable to interact with AP-1, NFAT promotes T cell anergy and exhaustion by inducing the expression of signaling molecules that dampen TCR signaling^27^. The colocalization of NFATc2 and c-Jun was significantly increased in the nucleus of *Sirt2*^−/−^ T cells compared to WT T cells upon TCR ligation (Extended Data Fig. 5e–g). We observed elevated transcription of *Nr4a1*, an early target of TCR signaling that encodes the protein Nur77, is rapidly induced upon T cell activation and used as a quantitative readout of TCR signal strength^28^, in naive and preactivated *Sirt2*^−/−^ T cells, as well as IL-15-differentiated OT-II and PMEL *Sirt2*^−/−^ T_M_-like cells compared to WT T cells (Fig. 3d–f). Upon TCR engagement, expression of the homing molecule CD62L undergoes downregulation through proteolytic shedding of its ectodomain^29^. *Sirt2*^−/−^ T cells stimulated with CD3 Abs exhibited rapid downregulation of surface CD62L expression, whereas WT T cells showed only a modest decrease (Fig. 3g). CD62L mediates lymphocyte rolling into the high endothelial venules of peripheral lymphoid tissues^29^. When carboxyfluorescein succinimidyl ester (CFSE)-labeled WT and CellTraceViolet (CTV)-labeled *Sirt2*^−/−^ T cells were stimulated with CD3 Abs to induce CD62L shedding and then intravenously (i.v.) transferred into recipient C57BL/6 mice, lower numbers of preactivated *Sirt2*^−/−^ compared to WT T cells were recruited from the bloodstream into the LNs or spleen of recipient mice (Fig. 3h,i). When nonstimulated naive WT and *Sirt2*^−/−^ T cells were injected into recipient mice at a 1:1 ratio, no significant differences in migration profiles were observed (Fig. 3j,k). Similar results were obtained when the labeling dyes were swapped and CTV-labeled WT and CFSE-labeled *Sirt2*^−/−^ T cells were adoptively transferred into C57BL/6 mice (Extended Data Fig. 5h,i). Altogether, these findings indicated that SIRT2 deficiency increased signaling through proximal and downstream TCR pathways.

### Loss of SIRT2 alters T cell development

**Fig. 4:**
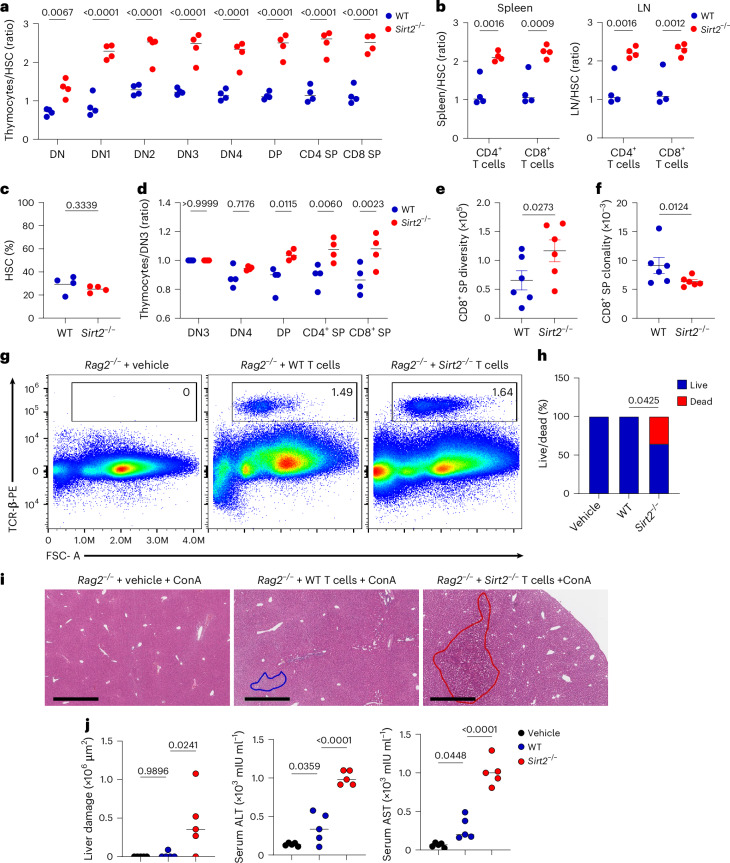
SIRT2 deficiency alters T cell development and exacerbates ConA-induced autoimmune activation. **a**, Ratio of donor-derived WT CD45.1⁺ and *Sirt2*^−/−^ CD45.2⁺ DN1–DN4, DP, CD4⁺ SP and CD8⁺ SP thymocytes in the thymus of lethally irradiated C57BL/6×C57BL/6.SJL F1 recipient analyzed 8 weeks after reconstitution with a 1:1 mixture of WT and *Sirt2*^−/−^ whole BM cells normalized to the input of WT versus *Sirt2*^−/−^ Sca-1^+^ c-kit^+^ HSCs in the BM of the same recipient mice at 8 weeks post-reconstitution. Each dot represents one mouse (*n* = 4 recipient mice). **b**, Ratio of WT CD45.1⁺ versus *Sirt2*^−/−^ CD45.1⁺ CD4⁺ and CD8⁺ T cells in the spleen and LN recipient mice as in **a** at 8 weeks post-reconstitution, normalized to the WT versus *Sirt2*^−/−^ HSC input in the BM of the same recipient mice at 8 weeks post-reconstitution (*n* = 4 recipient mice). **c**, Frequency of WT CD45.1⁺ versus *Sirt2*^−/−^ CD45.2⁺ Sca-1^+^ c-kit^+^ HSC at 8 weeks post-reconstitution in the BM of recipient mice as in **a** (*n* = 4 recipient mice). **d**, Ratio of DN3, DN4, DP, CD4⁺ SP and CD8⁺ SP thymocytes in the thymus of recipient mice as in **a** at 8 weeks post-reconstitution normalized to the number of WT versus *Sirt2*^−/−^ DN3 thymocytes. **e**,**f**, Frequency of productive TCRβ rearrangements (**e**) and TCR clonality index (**f**) in CD8⁺ SP thymocytes from WT and *Sirt2*^−/−^ mice determined by TCR deep sequencing (*n* = 6 mice per group). **g**, Representative flow cytometry analysis of TCRβ⁺ cells in the spleen of *Rag2*^−/−^ mice 24 h after adoptive transfer of vehicle or CD3⁺ T cells from WT or *Sirt2*^−/−^ donor mice (*n* = 5 mice per group). **h**, Survival of *Rag2*^−/−^ mice injected i.v. with 15 mg kg^−1^ body weight ConA 24 h after adoptive transfer of vehicle, WT or *Sirt2*^−/−^ CD3⁺ T cells, assessed 12 h after ConA injection. Vehicle, *n* = 9 mice; WT, *n* = 12 mice; and *Sirt2*^−/−^, *n* = 14 mice. **i**, Representative H&E staining of liver sections from *Rag2*^−/−^ mice adoptively transferred with vehicle, WT CD3⁺ T cells and *Sirt2*^−/−^ CD3⁺ T cells as in **h** at 24 h after ConA injection. Areas of necrosis are circled. Scale bar, 700 µm. **j**, Quantification of liver damage area, serum ALT and serum AST at 24 h after ConA injection in *Rag2*^−/−^ mice adoptively transferred with vehicle, WT CD3⁺ T cells and *Sirt2*^−/−^ CD3⁺ T cells as in **h**. *n* = 5 mice per group. Data are shown as mean ± s.e.m. Statistical analysis was performed using two-way ANOVA (**a**,**b**,**d**), two-tailed Student’s *t*-test (**c**), two-sided Dunn’s test (**e**,**f**), two-sided Fisher’s exact test (**h**) and one-way ANOVA (**j**). Data are representative of two (**f**–**n**) and four (**a**) independent experiments. Source data

**Extended Data Fig. 6:**
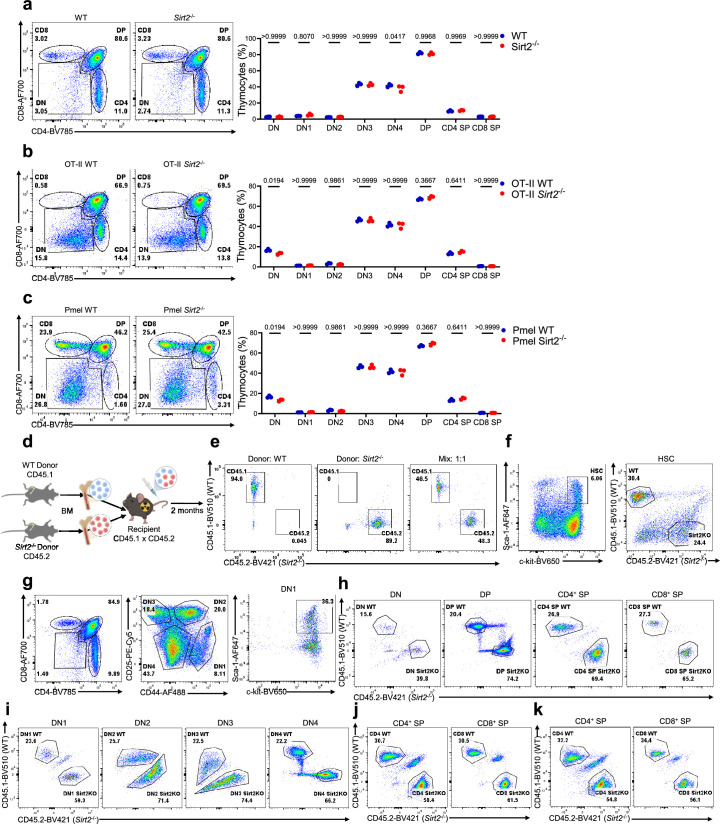
Loss of SIRT2 alters T cell development under competitive bone marrow reconstitution. **a–c**, representative flow-cytometric plots showing CD4 and CD8 distribution in WT and *Sirt2*^−/−^ thymi from C57BL/6 (**a**), OT-II (**b**), and PMEL (**c**) mice. Frequencies of DN (DN1–DN4), DP and SP thymocytes for CD4⁺ and CD8⁺ subsets are indicated in the right panel. Each dot = one mouse (n = 3 per group). **d**, schema of experimental design of mixed bone marrow chimeras generated by reconstituting lethally irradiated C57BL/6 × C57BL/6.SJL F1 recipient mice (n = 4) with a 1:1 mixture of bone marrow cells from WT (CD45.1⁺) and Sirt2⁻/⁻ (CD45.2⁺) donors. Mice were sacrificed 8 weeks after bone marrow transplantation. **e**, Flow-cytometric plots of WT CD45.1⁺ and *Sirt2*^−/−^ CD45.2⁺ bone marrow cells from WT (CD45.1), *Sirt2*^−/−^ (CD45.2) donor mice and the 1:1 mix prior injection. **f**, Flow-cytometric plots of hematopoietic stem cells (HSCs) identified as lineage⁻ (Lin⁻), mature T cell⁻ (CD3⁻), c-kit⁺ and Sca-1⁺ (left) and the distribution of WT CD45.1⁺ and *Sirt2*^−/−^ CD45.2⁺ HSC at 8 weeks post-reconstitution in the bone marrow of recipient mice. **g-i**, Flow-cytometric plots of donor-derived WT CD45.1⁺ and *Sirt2*^−/−^ CD45.2⁺ DN1–DN4, DP, CD4⁺ SP and CD8⁺ SP thymocytes in the thymus of lethally irradiated C57BL/6 × C57BL/6.SJL F1 recipient mice analyzed 8 weeks after reconstitution with a 1:1 mixture of WT and *Sirt2*^−/−^ bone marrow cells. Analysis was based on the distribution of CD4 and CD8 markers in CD45.1⁺ (WT) and CD45.2⁺ (*Sirt2*^−/−^) T cells. DN stages (DN1–DN4) were defined by CD44 and CD25 expression, and DN1 cells were further assessed for c-Kit⁺Sca-1⁺ expression. **j**,**k** Flow-cytometric plots of WT CD45.1⁺ *versus Sirt2*^−/−^ CD45.1⁺ CD4⁺ and CD8⁺ T cells in the spleen (**j**) and lymph nodes (**k**) of recipient mice as in **d** at 8 weeks post-reconstitution. Data are mean ± SEM. *P* values are determined by two-way ANOVA (**a-c**). Data are representative of three (**a-c**) and four (**d-k**) independent experiments. Source data

Because T cell development relies on pre-TCR and TCR signaling through LCK^30^, we investigated the potential impact of SIRT2 deficiency on thymocyte development in polyclonal TCR (C57BL/6) and monoclonal TCR transgenic (OT-II and PMEL) mice. *Sirt2*^−/−^ thymi from C57BL/6, OT-II and PMEL transgenic backgrounds showed normal distribution of double-negative (DN) DN1–DN4, double-positive (DP), CD4⁺ and CD8⁺ single-positive (SP) compartments compared to WT thymi (Extended Data Fig. 6a–c), suggesting that SIRT2 deficiency did not markedly affect the balance between positive and negative selection under steady-state conditions; however, when mixed BM chimeras (BMCs) were generated by transferring a 1:1 mixture of WT (CD45.1) and *Sirt2*^−/−^ (CD45.2) BM cells into lethally irradiated CD45.1 CD45.2 WT mice (Extended Data Fig. 6d–k), the frequency of *Sirt2*^−/−^ DN1, DN2, DN3, DN4, DP, CD4⁺ SP and CD8⁺ SP thymocytes was higher than WT thymocytes (Fig. 4a) and there was an increased output of mature *Sirt2*^−/−^ CD4⁺ and CD8⁺ SP T cells in the periphery, including the spleen and LNs in comparison to WT T cells (Fig. 4b). Equal engraftment of *Sirt2*^−/−^ and WT hematopoietic stem cells (HSCs) was observed in the bone marrow (BM) of recipient mice (Fig. 4c), whereas enrichment of *Sirt2*^−/−^ thymocytes was observed beyond the DN3 stage (Fig. 4d), indicating that loss of SIRT2 conferred a competitive advantage in thymocytes during thymopoiesis and selection.

**Extended Data Fig. 7:**
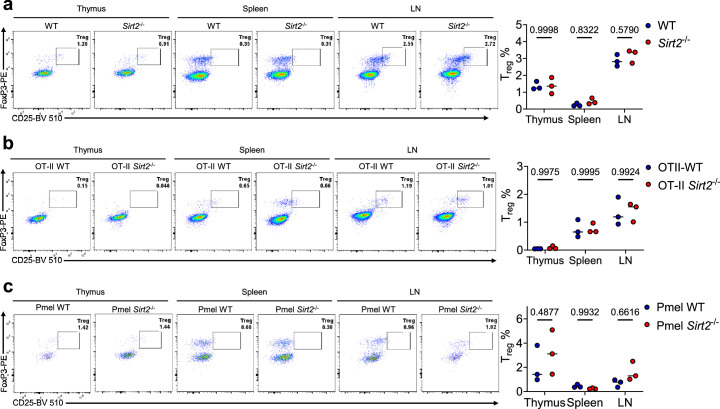
SIRT2 deficiency does not impact T_reg_ cell development. **a-c**, representative flow-cytometric plots (left) and frequencies (right) of T_reg_ cells based on CD44 and FoxP3 expressions in WT and *Sirt2*^−*/*−^ thymi, lymph nodes and spleens from C57BL/6 (**a**), OT-II (**b**) and PMEL (**c**) mice. Each dot represents one mouse (n = 3 mice per group). Data are mean ± SEM. P values were determined by two-way ANOVA. Data are representative of two independent experiments. Source data

To assess whether the TCR repertoire was impacted by loss of SIRT2, we performed deep TCR-β sequencing on CD8⁺ SP thymocytes isolated from WT and *Sirt2*^*−*/*−*^ mice. *Sirt2*^*−*/*−*^ CD8⁺ SPs exhibited a broader TCR-β repertoire diversity, with a significantly increased number of unique productive V(D)J rearrangements compared to WT CD8⁺ SPs (Fig. 4e). Repertoire diversity is inversely related to clonality, which measures the evenness of clonal distribution, with values near 1 indicating monoclonality or oligoclonality and values near 0 indicating maximal polyclonality^31^. Consistently, *Sirt2*^*−*/*−*^ CD8⁺ SP thymocytes displayed lower clonality compared to WT (Fig. 4f). Strong and persistent TCR signaling promotes FoxP3⁺ regulatory T (T_reg_) cell development in the thymus^32^. In C57BL/6, OT-II and PMEL mice, we observed no significant differences in the frequency of FoxP3⁺ T_reg_ cells between WT and *Sirt2*^−/−^ mice (Extended Data Fig. 7), indicating that SIRT2 deficiency did not impair T_reg_ cell development under steady-state conditions. Together, these findings indicated that SIRT2 deficiency offered a competitive advantage during T cell development and broadened TCR repertoire diversity.

### Loss of SIRT2 augments T cell responses to antigenic stimulation

**Extended Data Fig. 8:**
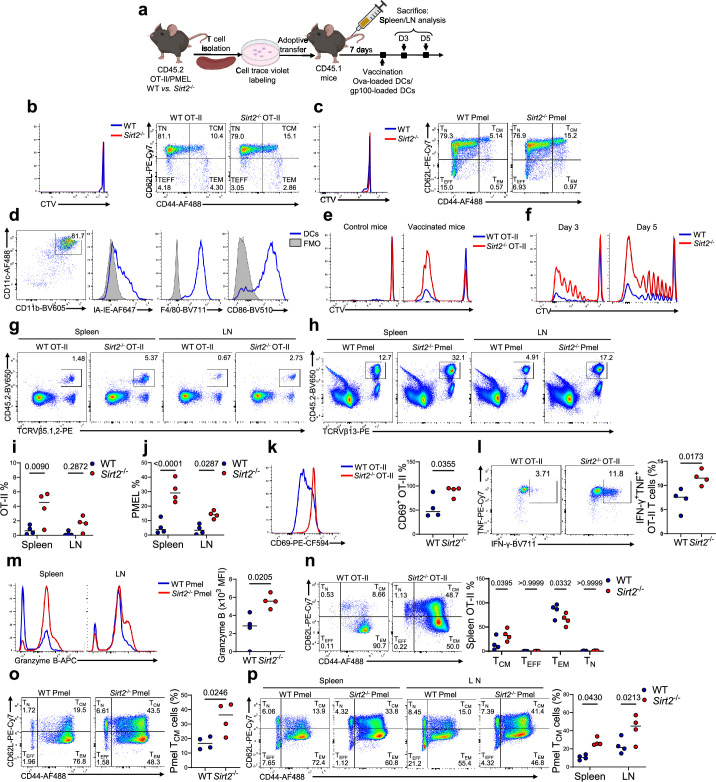
SIRT2 deficiency enhances OT-II and PMEL T cells proliferation, activation, and effector function following cognate antigen challenge *in vivo.* **a**, Experimental design of adoptive cell transfer showing CD4⁺ and CD8⁺ T cells isolated from spleens of WT and *Sirt2*^−/−^OT-II and PMEL mice, respectively, labeled with CTV and transferred into CD45.1⁺ congenic mice. One week later, recipient mice were vaccinated subcutaneously with OVA-loaded DCs (OT-II) or gp100-loaded DCs (PMEL). Mice were sacrificed on days 3 and 5 post-vaccination, and lymph nodes (LN) and spleens were collected. Created using BioRender.com. **b,c**, Flow-cytometric analysis of CTV labeling (left) and maturation status (right) of WT and *Sirt2*^−/−^ CD4⁺ OT-II (b) and CD8⁺ PMEL (c) T cells before transfer. **d**, Flow-cytometric analysis of DCs based on CD11c and CD11b expression; activation status assessed by I-A/I-E, F4/80 and CD86 expression. **e**, CTV dilution profiles of WT and *Sirt2*^−*/*−^ OT-II cells from vaccinated and non-vaccinated mice at day 3 post-immunization. **f**, CTV dilution profiles of WT and *Sirt2*^−*/*−^ PMEL T cells at day 3 and day 5 post-vaccination. **g–j**, Flow-cytometric plots of CD45.2⁺TCRVβ5.1/5.2⁺ OT-II (**g**) and CD45.2⁺TCRVβ13⁺ PMEL (**h**) transferred T cells in spleen and LN at day 5 post-vaccination. Frequencies of OT-II (**i**) and PMEL (**j**) T cells shown. Each dot = one mouse (n = 4 per group). **k,l**, Flow-cytometric plots (left) and frequencies (right) of CD69 (**k**) and dual TNF/IFN-γ expression (**l**) in WT and *Sirt2*^−*/*−^ OT-II T cells at day 5 post-immunization. Each dot = one mouse (n = 4 per group). **m**, Flow-cytometric analysis of Granzyme B expression in WT and *Sirt2*^−*/*−^ PMEL T cells at day 5 post-vaccination. Histograms show MFI in spleen. Each dot = one mouse (n = 4 per group). **n–p**, Flow-cytometric plots of T_N_, T_EFF_, T_EM_ and T_CM_ subsets based on CD62L and CD44 expression within CD4⁺ OT-II (**n**, day 5) and CD8⁺ PMEL T cells (**o**, day 3; **p**, day 5). Frequencies of T_CM_ cells indicated. Each dot = one mouse (n = 4 per group). Data are mean ± SEM. *P* values are determined by two-tailed Student’s *t*-test (**k**,**l,m,o**) and two-way ANOVA **(i,j,n,p**). Source data

To evaluate whether enhanced TCR signaling in *Sirt2*^*−*/*−*^ T cells translated into heightened T cell activation in physiological conditions in vivo, we transferred CTV-labeled CD4⁺ OT-II or CD8⁺ PMEL T cells from CD45.2 WT or *Sirt2*^*−*/*−*^ mice into CD45.1 WT mice, followed by vaccination with peptide-loaded dendritic cells (OVA for OT-II; gp100 for PMEL), or left unvaccinated as controls, and evaluated T cell response on day 3 and day 5 post-immunization (Extended Data Fig. 8a–d). Both donor *Sirt2*^−/−^ OT-II or PMEL T cells had higher frequencies and more pronounced CTV dilution in peripheral lymphoid tissues than donor WT T cells (Extended Data Fig. 8e–j), indicating that *Sirt2*^−/−^ T cells exhibited significantly greater expansion than WT T cells in vivo. *Sirt2*^−/−^ T cells also exhibited elevated expression of the activation markers CD25 and CD69 (Extended Data Fig. 8k), along with increased production of effector molecules, including interferon (IFN)-γ, tumor necrosis factor (TNF) and granzyme B (Extended Data Fig. 8l,m). No activation or proliferation was observed in unvaccinated mice (Extended Data Fig. 8e), confirming these responses were antigen-specific. *Sirt2*^−/−^ OT-II and PMEL T cells also exhibited a higher frequency of CD44^+^CD62L^+^ T_CM_ cells, a subset known for its superior proliferative capacity, long-term persistence and robust recall responses, all of which are associated with sustained immune protection and enhanced antitumor immunity^33^, in the spleen and LNs of recipient mice (Extended Data Fig. 8n–p).

**Extended Data Fig. 9:**
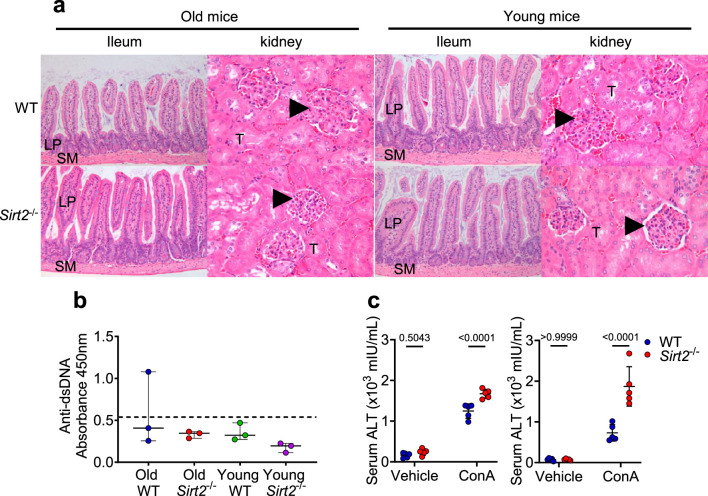
Sirt2 deficiency does not induce spontaneous autoimmunity but exacerbates ConA-induced autoimmune activation. **a**, Small intestines and kidneys, from old ( > 1 year) and young (7 weeks) WT and *Sirt2*^−/−^ mice, were fixed, sectioned, and stained with H&E. All mice isplayed normal glomeruli (arrows) and tubules (T) with no evidence of inflammation in the ileum lamina propria (LP) or submucosa (SM). Images from one representative mouse of *n* = 3 per group are shown. **b**, Anti–double-stranded DNA (dsDNA) activity measured by ELISA on sera from old ( > 1 year) and young (7-week-old) WT and *Sirt2*^−/−^ mice. Technical replicates (n = 3) per mouse were averaged, and net A_450_ values (after blank subtraction) were plotted per group. Each dot represents one mouse (n = 3 per group). The dashed line represents the limit of normal/non-immune range values (A_450_ < 0.425; anti-dsDNA activity < 50 U/ml). **c**, Serum ALT (left) and AST (right) levels in WT and *Sirt2*^−/−^ mice 24 h after injection of vehicle or ConA to induce fulminant hepatitis. Each dot represents one mouse (n = 3 per group). Data are mean ± SEM. *P* values are determined by one-way ANOVA (b) and two-way ANOVA (**c**). Data are representative of two independent experiments. Source data

We next evaluated whether SIRT2 deficiency altered the balance between immune tolerance and autoimmunity by assessing spontaneous autoimmune manifestations in aged mice. Comprehensive necropsies and histological analyses showed no evidence of spontaneous inflammation or autoimmune pathology in young and aged WT and *Sirt2*^−/−^ mice (Extended Data Fig. 9a). Additionally, no anti-double-stranded DNA activity was detected in the sera of either young or aged WT and *Sirt2*^*−*/*−*^ mice (Extended Data Fig. 9b), suggesting that central tolerance mechanisms remained intact, and that the lowered TCR threshold in *Sirt2*^*−*/*−*^ thymocytes still permitted effective negative selection of autoreactive clones; however, in the concanavalin A (ConA)-induced hepatitis model of T cell-driven inflammation^34^, *Sirt2*^*−*/*−*^ mice exhibited significantly increased liver damage following ConA injection, as indicated by elevated serum alanine aminotransferase (ALT) and aspartate aminotransferase (AST) compared to WT mice, whereas no differences were observed under basal conditions (Extended Data Fig. 9c). To confirm that this phenotype was T cell-intrinsic, WT or *Sirt2*^*−*/*−*^ CD3⁺ donor T cells were adoptively transferred into immunodeficient *Rag2*^*−*/*−*^ mice followed by ConA administration. Efficient T cell engraftment was confirmed by flow cytometry analysis of spleens from recipient *Rag2*^*−*/*−*^ mice (Fig. 4g). *Rag2*^*−*/*−*^ mice that received *Sirt2*^*−*/*−*^ T cells exhibited exacerbated liver damage and increased mortality compared to *Rag2*^*−*/*−*^ mice that received WT T cells (Fig. 4h). Histological analysis of hematoxylin and eosin (H&E)-stained liver sections revealed widespread hepatic necrosis in *Rag2*^*−*/*−*^ mice that received *Sirt2*^*−*/*−*^ T cells (Fig. 4i) and serum ALT and AST were significantly higher in the *Sirt2*^*−*/*−*^ T cell group compared to the WT T cells or vehicle group (Fig. 4j). Thus, while SIRT2 deficiency did not promote spontaneous autoimmunity, the heightened T cell activation in *Sirt2*^*−*/*−*^ T cells lead to amplified responses to induced autoimmunity.

### SIRT2 blockade restores TCR response and delays tumor growth

**Fig. 5:**
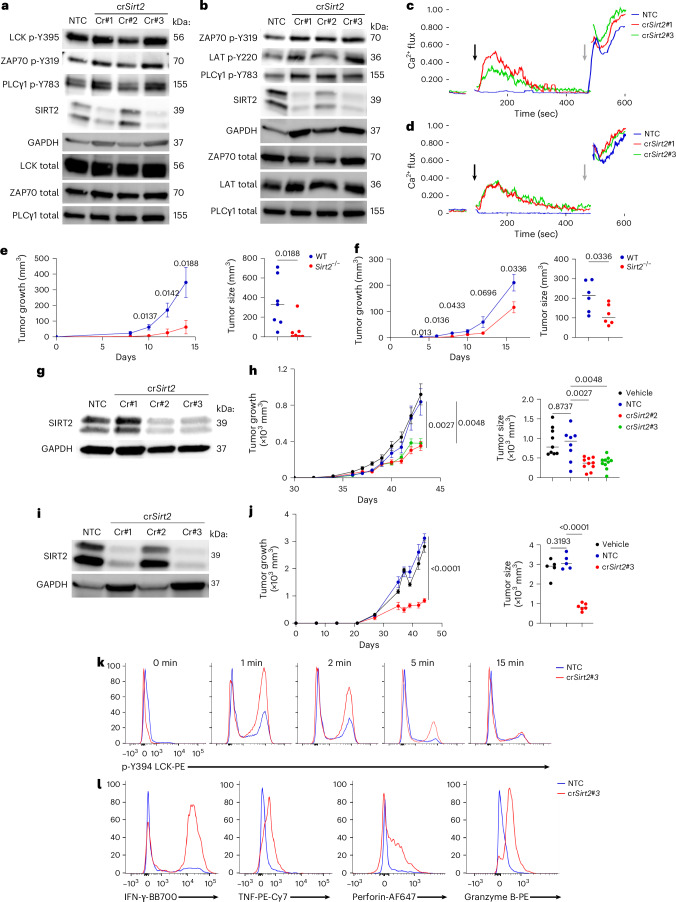
SIRT2 inhibition restores TCR responsiveness in exhausted human TILs and delays tumor growth in vivo. **a**,**b**, Immunoblot analysis of phosphorylated and total LCK, ZAP70 and PLCγ1 in TILs from NSCLC patient no. 3 (**a**) and patient no. 7 (**b**) that were electroporated with Cas9–crRNA NTC or Cas9–crRNAs targeting SIRT2 (Cr#1, Cr#2 and Cr#3) and stimulated with 2.5 µg ml^−1^ CD3 Abs for 5 min. **c**,**d**, Ca^2+^ flux analysis of NSCLC patient no. 3-derived (**c**) and patient no. 7-derived (**d**) TILs loaded with Indo-1 AM and labeled with 2.5 µg ml^−1^ biotinylated CD3 Abs in response to 2.5 µg ml^−1^ streptavidin (black arrow) and ionomycin (gray arrow). **e**, Growth curve at day 0–15 (left) and volume at day 14 (right) of s.c. Yumm1.7 tumors in WT and *Sirt2*^−/−^ mice (*n* = 7 mice per group). **f**, Growth curves at day 0–15 (left) and volumes at day 16 (right) of s.c. *KPMSH2*^KIN^ tumors in WT and *Sirt2*^−/−^ mice (*n* = 6 mice per group). **g**,**i**, Immunoblot of SIRT2 expression in TILs expanded from SCLC (**g**) and NSCLC (**i**) donors 1 month post-electroporation with Cas9–crRNA NTC or Cas9–crRNAs targeting SIRT2 (Cr#1, Cr#2 and Cr#3), with GAPDH (**g**) and β-actin (**i**) as loading controls. **h**, Growth curves at day 0–15 (left) and volume at day 43 (right) of NSCLC tumor cells injected s.c. in NSG mice treated with vehicle, NTC or SIRT2-deficient TILs (cr*Sirt2*#2, cr*Sirt2*#3) at 21 days after tumor injection. Vehicle (*n* = 9 mice), NTC (*n* = 8 mice), cr*Sirt2*#2 (*n* = 10 mice) and cr*Sirt2*#3 (*n* = 10 mice). **j**, Growth curves at day 0–50 (left) and volume at day 44 (right) of NSCLC tumor cells injected s.c. in NSG mice treated with vehicle, NTC or SIRT2-deficient TILs (cr*Sirt2*#3) at 21 days after tumor injection. Vehicle (*n* = 5 mice), NTC (*n* = 5 mice) and cr*Sirt2*#3 (*n* = 6 mice). **k**, Flow cytometry analysis of p-Y394 LCK before and after stimulation with 2.5 µg ml^−1^ CD3 Abs for 0, 1, 2, 5 and 15 min in NTC and cr*Sirt2*#3 TILs isolated at end point from NSCLC-PDX tumors grown in NSG mice infused with NTC or cr*Sirt2*#3 TILs, as in **j**. **l**, Flow cytometry analysis of IFN-γ, TNF, perforin and granzyme B expression in NTC and cr*Sirt2*#3 TILs isolated at end point from NSCLC-PDX tumors grown in NSG mice infused with NTC or cr*Sirt2*#3 TILs, as in **j**. Data are shown as mean ± s.e.m. Statistical analysis was performed using a two-tailed Student’s *t*-test (**e**,**f**) and one-way ANOVA (**h**,**j**). Data are representative of two independent experiments. Source data

**Extended Data Fig. 10:**
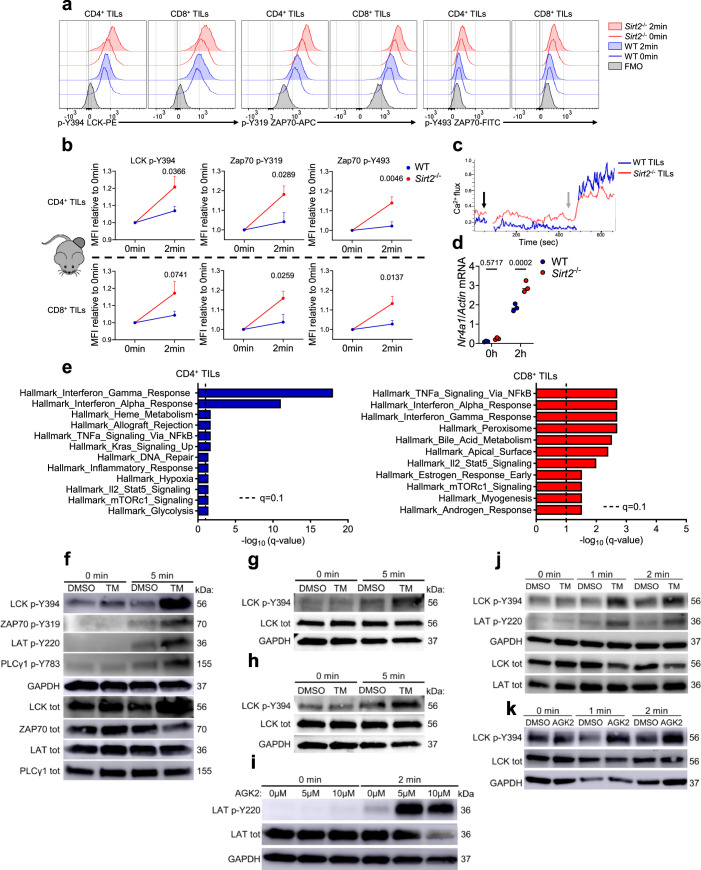
SIRT2 deficiency restores TCR responsiveness in exhausted mouse and human TILs. **a**, Flow-cytometric analysis of phospho-Y394 LCK, phospho-Y319 ZAP70 and phospho-Y493 ZAP70 before and 2 min after stimulation with 2.5 µg/ml CD3 Abs in WT and *Sirt2*^−/−^ CD4⁺ and CD8⁺ TILs isolated from B16F10 subcutaneous tumors. FMO, fluorescence minus one. **b**, Mean fluorescence intensity (MFI) of phospho-Y394 LCK, phospho-Y319 ZAP70 and phospho-Y493 ZAP70 before and 2 min after TCR stimulation normalized to baseline. Each dot represents one mouse (n = 3 mice per group). **c**, Calcium flux in WT and *Sirt2*^−/−^ B16F10 TILs loaded with Indo-1 AM and labeled with biotinylated CD3 2.5 µg/ml Abs. Baseline fluorescence ratio was recorded for 60 s before simulation with 2.5 µg/ml streptavidin (black arrow). Ionomycin was added later (gray arrow) as a control of maximal Ca^2+^ release. **d**, *Nr4a1* mRNA expression relative to actin quantified by qRT-PCR in resting WT and *Sirt2*^−/−^ B16F10 TILs and 2 h after stimulation with 2.5 µg/ml plate-bound CD3 Abs (n = 3 biological replicates). **e**, Gene set enrichment analysis (GSEA) of upregulated differentially expressed genes in *Sirt2*^−/−^ *versus* WT CD4⁺ (left) and CD8⁺ (right) B16F10 TILs. False discovery rate (FDR) q < 0.1 was used as the cut-off. **f–i**, Immunoblot analysis of phosphorylated and total proximal TCR signaling molecules before and after stimulation with 2.5 µg/ml CD3 Abs for 0–5 min in human TILs from four lung cancer patients pre-treated with vehicle (DMSO) or SIRT2 inhibitor TM (2 µM; **f–h**) or AGK2 (5–10 µM; **i**) for 24 h. **j,k**, Immunoblot analysis of phospho-Y394 and total LCK before and after stimulation with 2.5 µg/ml CD3 Abs for 0–2 min in human CD3⁺ T cells from two healthy donors pre-treated with vehicle (DMSO) or SIRT2 inhibitor TM (2 µM; **j**) or AGK2 (5 µM; **k**) for 24 h. GAPDH (**f-k**) levels were used as loading controls. Data are mean ± SEM. *P* values are determined by two-way ANOVA (**b,d**). Data are representative of one (**e**), two (**f-k**) and three (**a-d**) independent experiments. Source data

Tumor-reactive T cells typically exhibit weak TCR signaling and low affinity for tumor antigens^35^. Despite attempts to isolate higher-affinity T cell clones or engineer affinity-enhanced TCRs to improve the antitumor response, success has been limited^36,37^. Therefore, we examined the TCR responsiveness of murine melanoma tumor-infiltrating lymphocytes (TILs) obtained from s.c. B16F10 tumors in WT and *Sirt2*^*−*/*−*^ mice. Mouse *Sirt2*^*−*/*−*^ TILs displayed increased phosphorylation of LCK (Y394) and ZAP70, elevated Ca^2+^ mobilization and higher *Nr4a1* transcription compared to WT TILs (Extended Data Fig. 10a–d). Additionally, pathway enrichment analysis of differentially expressed genes revealed upregulation of pathways downstream of TCR signaling in *Sirt2*^*−*/*−*^ compared to WT CD4^+^ and CD8^+^ TILs (Extended Data Fig. 10e). We also examined the impact of SIRT2 inhibition on TCR response in human T cells obtained from healthy donors and TILs isolated from non-small cell lung cancer (NSCLC) or small cell lung cancer (SCLC) biopsies. The selective SIRT2 inhibitors thiomyristoyl (TM)^38^ and AGK2 (ref. ^39^) or SIRT2 CRISPR/Cas9 knockout restored proximal TCR signaling and Ca^2+^ mobilization in human T cells from healthy donors and exhausted TILs from patients with NSCLC and SCLC following TCR engagement (Fig. 5a–d and Extended Data Fig. 10f–k). When *Sirt2*^−/−^ and WT mice were s.c. challenged with either Yumm1.7 melanoma cells or *KPMSH2*^*KIN*^ lung cancer cells^40^ and tumor growth was monitored over time, tumor growth was significantly delayed in *Sirt2*^*−*/*−*^ mice compared to WT mice in both models (Fig. 5e,f), demonstrating the potential therapeutic benefit of SIRT2 deficiency in enhancing antitumor immunity.

We also evaluated the impact of SIRT2 manipulation in the context of TIL therapy using patient-derived xenograft (PDX) models of NSCLC and SCLC, each paired with autologous TILs isolated from the same patient tumor. When tumor-bearing PDX mice were infused with either nontargeting control (NTC) human TILs or CRISPR-deleted SIRT2-deficient human TILs, mice that received Sirt2-deficient TILs showed significantly delayed tumor growth compared to those receiving vehicle or NTC TILs in both NSCLC and SCLC models (Fig. 5g–j). To determine whether the delayed tumor growth was mediated by enhanced TCR signaling, we isolated infused human NSCLC TILs from the tumors of PDX mice and evaluated the effects of SIRT2 deletion on their TCR responsiveness. Sirt2-deficient human NSCLC TILs exhibited increased phosphorylation of LCK at Y394 following TCR stimulation compared to NTC controls (Fig. 5k). These Sirt2-deficient TILs also showed elevated cytokine production (Fig. 5l), further indicating enhanced activation. Collectively, these findings highlighted SIRT2 blockade as a promising therapeutic strategy to enhance antitumor immunity by boosting TCR signaling in TILs.

## Discussion

Here, we showed that SIRT2 regulated TCR signaling through deacetylation of LCK at lysine 228, a conserved residue within its LR. Acetylation at K228 disrupted the intramolecular SH3–LR interaction, favoring an open, active LCK conformation. Consequently, SIRT2 loss amplified proximal TCR signaling, Ca²⁺ flux and downstream activation of NFAT and ERK, resulting in heightened T cell responses.

LCK activation is regulated by conformational changes^41^. K228 within the LCK-LR, a highly conserved domain^42^, was hyperacetylated in mouse *Sirt2*^−/−^ T cells and human Jurkat cells compared to WT controls. While X-ray crystallography evidence for conformational changes induced by K228 acetylation is lacking, disruption of the LR–SH3 interaction by K228 acetylation supports a model in which SIRT2 controls the stoichiometry of the closed, primed and open form of LCK, thereby modulating proximal TCR signaling. The LCK^K228Q^ mutant, which mimicked K228 acetylation, reproduced the phenotype observed in SIRT2-deficient T cells, confirming that K228 acetylation enhances LCK activity. Notably, mutations of neighboring residues (K232E and P233G) on the LR of LCK also increases LCK activity^42^, supporting a structural link between this region and LCK conformational changes.

K228 acetylation increased upon T cell activation but declined in T_M_-like cells and melanoma TILs. This pattern parallels SIRT2 upregulation in T_M_ cells and TILs^21^, suggesting that SIRT2-mediated deacetylation of LCK in these states. The accumulation of acetylated LCK in T_EFF_-like cells likely stabilizes an open LCK conformation and facilitates robust signaling, whereas reduced LCK acetylation in T_N_, T_M_ and exhausted T cells may favor the inactive state and limit responsiveness in these quiescent cells.

LCK has key roles in thymic development^43,44^. Although *Sirt2*^−/−^ mice displayed normal thymic architecture at steady state, mixed WT:*Sirt2*^−/−^ BMCs showed increased *Sirt2*^−/−^ thymocytes from the DN3 stage onward, consistent with enhanced pre-TCR signaling during β-selection. Stronger pre-TCR or TCR signaling in *Sirt2*^−/−^ thymocytes may therefore promote their developmental progression. We also detected an increased frequency of *Sirt2*^−/−^ early thymic progenitor cells in chimeric mice, although these cells lack pre-TCR and TCR expression, and the mechanism underlying this increase remains unclear. The broader TCR repertoire in *Sirt2*^−/−^ mice likely reflects a lower TCR signaling threshold that allows positive selection of more low-affinity clones while maintaining negative selection and self-tolerance. Consistent with this, *Sirt2*^−/−^ mice lacked spontaneous autoimmunity but showed greater susceptibility to ConA-induced hepatitis, indicating that excessive TCR activation may exacerbate inflammatory responses. The subtle T cell developmental phenotype of *Sirt2*^−/−^ mice closely resembles that observed in *c-Cbl*^−/−^ mice, in which lack of E3 ubiquitin ligase activity at the level of proximal TCR signaling results in normal thymic development, but enhanced positive selection and a broader TCR repertoire due to lower TCR signaling thresholds^45,46^. While strong and persistent TCR signaling seems to drive Foxp3^+^ T_reg_ cell development in the thymus^32^, *Sirt2*^−/−^ mice did not exhibit significant changes in T_reg_ cell development compared to WT mice, with or without tumor challenge^21^.

Tumor-reactive T cells typically display weak TCR signaling due to central and peripheral tolerance mechanisms eliminating high-affinity clones^47^ and to chronic inhibitory signaling in tumors^48^. Our data indicate that SIRT2 contributes to this hyporesponsive state, as its expression increased in activated and tumor-infiltrating T cells. Pharmacological SIRT2 inhibition restored TCR signaling in exhausted human TILs, suggesting that SIRT2 acts as a brake on TCR responsiveness within tumors. These findings are particularly relevant in the context of ongoing efforts to enhance T cell-based immunotherapies by restoring functional TCR signaling^49^. While promising, such approaches often fail in the metabolically restricted tumor microenvironment, where T cell persistence and responsiveness are severely compromised^50^. SIRT2 inhibition enhances both T cell metabolic fitness^21^ and proximal TCR signaling, providing dual metabolic and signaling benefits. Together, these effects position SIRT2 inhibition as a unique strategy to restore T cell responsiveness in metabolically suppressed tumors and to improve the efficacy of T cell and checkpoint-based immunotherapies.

## Methods

### Ethics statement

All animal experiments were performed under the approval of the Institutional Animal Care and Use Committee of Mayo Clinic (animal study protocols A00007348 and A00007297) and were conducted in accordance with the regulations of the Association for Assessment and Accreditation of Laboratory Animal Care International (AAALAC International).

### Mice

C57BL/6J mice, C57BL/6SJL mice, PMEL mice with gp100-reactive TCR: B6.Cg-*Thy1*^a^/Cy Tg(TcraTcrb)8Rest/J, OT-II mice with MHC II-restricted OVA-specific TCR: B6.Cg-Tg(TcraTcrb)425Cbn/J, *Sirt2*^−/−^ mice: B6.129-*Sirt2*^tm1.1Fwa^/J and B6.Cg-Rag2tm1.1Cgn/J (*Rag2*^−/−^) and NSG mice: NOD.Cg-*Prkdc*^scid^ *Il2rg*^tm1Wjl^/SzJ were purchased from The Jackson Laboratory. PMEL mice and OT-II mice were crossed with *Sirt2*^−/−^ mice to generate *Sirt2*^−/−^ PMEL mice and *Sirt2*^−/−^ OT-II mice, respectively. C57BL/6J (CD45.2) and C57BL/6.SJL-Ptprca (CD45.1) mice were crossed to generate F1 hybrid CD45.1 × CD45.2 mice. All mice were bred and maintained under specific-pathogen-free conditions at the animal facility of the Mayo Clinic. Mice were housed under controlled environmental conditions with a 12-h light–dark cycle, ambient temperature of 20–24 °C and relative humidity of 40–60%, with ad libitum access to autoclaved food and water. All animal protocols were approved by the Institutional Animal Care and Use Committee of the Mayo Clinic and conducted in accordance with institutional and National Institutes of Health guidelines. Mice were used at 7–8 weeks of age, with age- and sex-matched controls included in all experiments.

### Mouse T cell culture

Spleens, LNs and thymi collected from WT and *Sirt2*^−/−^ mice on C57BL/6, PMEL or OT-II backgrounds and processed into single-cell suspensions. CD3^+^, CD4^+^ and CD8^+^ T cells were negatively enriched using Mouse Pan T cell, CD4^+^ T cell and CD8^+^ T cell isolation kits (Miltenyi Biotec), respectively according to the manufacturer’s instructions. The purity of the isolated cells was confirmed by flow cytometry (>95%). Purified T cells were cultured in complete RPMI-1640 medium (Thermo Fisher Scientific) supplemented with 10% fetal bovine serum (FBS; Biowest) and 1% penicillin–streptomycin (P/S) (Thomas Scientific).

To generate preactivated T cells (T_EFF_-like cells), WT and *Sirt2*^−/−^ T cells were stimulated under the following conditions for 48 h: (1) C57BL/6T cells were activated on anti-CD3-coated plates (5 μg ml^−1^, clone 145-2C11; BioXCell); (2) PMEL T cells were stimulated with gp100_25–33_ peptide (1 μg ml^−1^; AnaSpec); and (3) OT-II T cells were stimulated with OVA_323–339_ peptide (10 μg ml^−1^; InvivoGen).

To generate T_M_-like cells ex vivo, PMEL and OT-II splenocytes were activated with 1 μg ml^−1^ gp100_25–33_ (AnaSpec) and 10 μg ml^−1^ OVA_323–339_ (InvivoGen), respectively for 3 days and subsequently cultured in the presence of 10 ng ml^−1^ IL-15 (R&D) for 4 days.

For metabolic inhibition experiments, purified CD3^+^ T cells from WT and *Sirt2*^−/−^ mice were treated with either 2-deoxy-D-glucose (2-DG; 1 mM; Sigma-Aldrich) to inhibit glycolysis or oligomycin (100 nM; Sigma-Aldrich) to block mitochondrial ATP synthase. Inhibitors were added for 2 h before stimulation on anti-CD3-coated plates (5 μg ml^−1^, clone 145-2C11; Bio X Cell) for the indicated time points (0–5 min). Cells were then collected and lysed for immunoblot analysis of proximal TCR signaling.

### Human PBMCs

Human peripheral blood mononuclear cells (PBMCs) were obtained from healthy donors (LifeSouth) by density gradient centrifugation using Ficoll-Paque PLUS Media (GE Healthcare). CD3^+^ T cells were negatively enriched using a human Pan T cell isolation kit (Miltenyi Biotec). Enriched CD3^+^ T cells were cultured in complete RPMI medium and activated with anti-CD3 Abs-coated plates (5 μg ml^−1^, OKT-3, BioXCell).

### Primary human cell culture

Primary cultures of SCLC cells were established by plating 1 ml of malignant pleural effusion in the presence of the Rho kinase inhibitor Y-27632 (10 µM; Tocris) onto irradiated fibroblast feeder cells derived from a different patient with lung cancer, as previously described^51^. Cells were cultured in F12:DMEM (Thermo Fisher Scientific) supplemented with 10% FBS, insulin (5 µg ml^−1^, Sigma-Adrich), EGF (10 ng ml^−1^; Sigma-Aldrich), hydrocortisone (400 ng ml^−1^; Sigma-Aldrich), adenine (24 µg ml^−1^; Sigma-Aldrich) and Y-27632 (Tocris). After three passages, cultures were maintained without feeder cells.

### Rapid expansion protocol

Human TILs were isolated from tumor biopsies of patients with advanced NSCLC or SCLC. Collection and expansion of human TILs were approved by an Institutional Review Board protocol. All human samples provided were and remained de-identified.

For TIL expansion, 5 × 10^5^ TILs were stimulated with 30 ng ml^−1^ human anti-CD3 (OKT-3, BioLegend) in the presence of 1 × 10^8^ irradiated (5,000 rad) allogenic PBMC feeder cells. TILs were cultured in REP Media I consisting of RPMI-1640 medium supplemented with 10% human AB serum (Valley Biomedical), 2 mM L-glutamine (HyClone, Thermo Fisher Scientific), 1 mM HEPES (Sigma-Aldrich), 1% P/S (Thomas Scientific), 50 µg ml^−1^ gentamicin (Thermo Fisher Scientific) and 50 μM β-mercaptoethanol (Thermo Fisher Scientific). On day 5, 70 % of the medium was replaced with REP Media II consisting of a 1:1 (v:v) mixture of REP Media I and AIM V (Invitrogen), supplemented with recombinant human IL-2 (3,000 IU ml^−1^; PeproTech). After 14 days, TILs were collected, counted and rested before future analysis.

Human TILs were cultured in complete RPMI medium in the presence of dimethylsulfoxide (DMSO) (vehicle) or the indicated concentrations of thiomyristoyl (2 µM, Selleck Chemicals) or AGK2 (5–10 µM, Selleck Chemicals).

### Cell lines

Human Jurkat cells, murine EL4 cells, B16F10 and Yumm1.7 murine melanoma cells, and 293T human embryonic kidney cells were obtained from the American Type Culture Collection. Cells were passaged minimally and maintained in complete Dulbecco’s modified Eagle medium (DMEM)/F12 (Thermo Fisher Scientific) containing 10% FBS (Biowest) and 1% P/S (Thomas Scientific. Cell lines were routinely tested for negative mycoplasma contamination.

KPMSH2^KIN^ lung cancer cells, which express the same epitope recognized in human G12D-mutated KRAS, were obtained from J. Conejo-García (Duke University)^52^. Cells were routinely cultured in RPMI-1640 (Thermo Fisher Scientific) supplemented with 10% FBS (Biowest), 1% P/S, 2 mM L-glutamine and 0.5 mM sodium pyruvate (all from Thermo Fisher Scientific).

### Subcutaneous tumor models

Anesthetized mice were injected s.c. in the flank with either 2 × 10^5^ B16F10 or Yumm1.7 melanoma cells, or 1 × 10^6^ KPMSH2^KIN^ lung cancer cells, suspended in 100 μl of sterile PBS. Tumor volumes were measured twice weekly for 2 weeks and calculated using the formula: $\mathrm{volume}=\mathrm{length}\times {\mathrm{width}}^{2}\div2$.

### Histology

Comprehensive necropsies were performed for autoimmune pathology assessment. Animals were killed, and a wide range of tissues were collected, including the entire alimentary tract (esophagus, stomach, duodenum, jejunum, ileum, cecum and colon), mesentery, mesenteric, inguinal and cervical LNs, skin, subcutis, skeletal muscle, salivary glands, reproductive tract, liver, gall bladder, spleen, pancreas, kidneys, adrenal glands, larynx, trachea with attached thyroid and parathyroid glands, heart, thymus and lungs. The small intestine was insufflated with 10% neutral buffered formalin and rolled into Swiss rolls for histological processing. All tissues were fixed in 10% neutral buffered formalin until further processing, then embedded in paraffin, sectioned at 4–5-μm thickness and mounted on poly-L-lysine-coated slides. Sections were stained with H&E to assess general cellular morphology. Histopathological evaluation was performed by light microscopy, focusing on features indicative of autoimmunity, including glomerular basement membrane thickening and inflammation of the ileal lamina propria or submucosa.

In the ConA-induced hepatitis model, livers were processed similarly and stained with H&E. Areas of hepatic injury, including apoptosis and necrosis, were quantified using ImageScope software. For each mouse, liver injury was assessed using three tissue sections taken at different levels, and the cumulative injury area was calculated and reported in µm².

### Detection of serum autoantibodies

Anti-dsDNA Abs were detected by ELISA kit (Alpha Diagnostics). Sera were diluted 100-fold before assay, and the manufacturer’s protocol was followed for procedure and determination of positive versus negative results.

### ConA-induced hepatitis

Concanavalin A (ConA; Sigma-Aldrich) was administered i.v. at a dose of 15 mg kg^−1^. Mice were killed 24 h later, and livers were collected for histopathological analysis. Sera were collected and analyzed for ALT and AST levels using ELISA kits (Abcam) following the manufacturer’s instructions.

### Patient-derived xenograft mouse models

NSCLC-PDX models were generated by implanting fresh tumor biopsies from patients with NSCLC into 4–6-week-old NSG mice. At the end point, tumors were collected, enzymatically digested into single cells and resuspended in a 1:1 mixture of RPMI-1640 and Matrigel (Thermo Fisher Scientific). A total of 1 × 10^6^ cells were injected subcutaneously into the flanks of new NSG recipient mice.

SCLC PDX models were established by injecting 1 × 10^6^ primary culture cells from SCLC suspended in a 1:1 mix of RPMI-1640 and Matrigel (Thermo Fisher Scientific).

For both models, once tumors became palpable, mice were randomized into control and treatment groups and i.v. administered 5 × 106 patient-matched (autologous) TILs. Tumor volumes were monitored three times per week and calculated as: $\mathrm{volume}=\mathrm{length}\times {\mathrm{width}}^{2}\div2$.

### Isolation of TILs

Lymphocytes from s.c. tumors were isolated by dicing the tissues followed by enzymatic digestion in PBS containing collagenase type 4 (2 mg ml^−1^, Worthington Biochemical) and DNase I (0.25 mg ml^−1^, Sigma-Aldrich) for 45 min with occasional shaking at 37 °C. Cell suspensions were successively filtered through 100-μm and 40-μm cell strainers (Thermo Fisher Scientific) to obtain single-cell suspension followed by a PBS wash and red blood cell lysis using RBC Lysis Buffer (BioLegend). TILs were finally isolated by density gradient centrifugation using Percoll (GE Healthcare) and were further purified using a CD3ε MicroBeads kit (Miltenyi Biotec) according to the manufacturer’s instructions. Fresh TILs were used directly for TCR signaling studies.

### Construction and production of lentivectors

Human and mouse LCK point mutations (K228Q/R) expressing vectors were generated by site-directed mutagenesis from human and mouse pLenti-LCK-mGFP-P2A-Puro vectors (OriGene) using a Q5 Site-Directed Mutagenesis kit (New England Biolab). The following primers were used to generate human LCKK228Q forward: 5’-CCAGACCCAGcAGCCCCAGAAGC-3’ and reverse: 5’-CAGGGGCGGCTCAACCGT-3’; human LCKK228R forward: 5’- CAGACCCAGAgGCCCCAGAAGCC-3’ and reverse: 5’- GCAGGGGCGGCTCAACCG-3’; mouse LCKK228Q forward: 5’- CCAGACCCAGcAGCCCCAGAA-3’ and reverse: 5’- CAAGGACGGCTCAACTTTG-3’; and mouse LCKK228R forward: 5’- CAGACCCAGAgGCCCCAGAAA-3’ and reverse: 5’- GCAAGGACGGCTCAACTTTG-3’.

The mouse SIRT2 open reading frame was cloned from the pCMV6-Sirt2-expressing vector (Origene) into the pLenti-C-mGFP-P2A-Puro Tagged Cloning Vector (Origene).

Lentivectors were generated using Lenti-vpak Packaging kit (Origene) by transfecting 293T cells with the lentiviral expressing vectors and the packaging plasmids. Viral supernatants were collected 48 and 72 h after transfection, spun at 3,000 rpm for 10 min and filtered through 0.45-mm filters. Lentivectors were finally concentrated using Lenti-X Concentrator (Takara Bio) according to the manufacturer’s instructions (Clontech).

### CRISPR/Cas9-mediated knockout of SIRT2 and LCK

crRNA targeting human *SIRT2* cr#1: 5’- UCUGGGAGAAUAAGUUCCGCGUUUUAGAGCUAUGCU-3’, cr#2: 5’- UCUGCUGGACGAGCUGACCUGUUUUAGAGCUAUGCU-3’ and cr#3: 5’- GACUUUCGCUCUCCAUCCACGUUUUAGAGCUAUGCU-3’, crRNA targeting human *LCK* cr#1: 5’- AUCCGUAAUCUGGACAACGGGUUUUAGAGCUAUGCU-3’, cr#2: 5’-GACCCACUGGUUACCUACGAGUUUUAGAGCUAUGCU-3’ and cr#3: 5’-GCCCUCUCACGACGGAGAUCGUUUUAGAGCUAUGCU-3’, crRNA targeting mouse *Lck* cr#1: 5’-GCGGACUAGAUCGUGCAAUCGUUUUAGAGCUAUGCU-3’, cr#2: 5’-GCUUUCGCCACGAAGUUGAAGUUUUAGAGCUAUGCU-3’ and cr#3: 5’- GUCGAAGUCUCUGACCGACAGUUUUAGAGCUAUGCU-3’, and crRNA NTC (all from IDT) were reconstituted to 100 µM in Nuclease-Free Duplex Buffer (IDT). crRNAs were then mixed at equimolar concentrations with Alt-R CRISPR-Cas9 tracrRNA, ATTO 550 (IDT) in a sterile PCR tube. crRNA–tracrRNA duplexes were annealed by heating at 95 °C for 5 min in PCR thermocycler, then slowly cooled to room temperature. Then, 9 µl of crRNA–tracrRNA duplexes were mixed with 6 µl (180 pmol) of TrueCut Cas9 Protein v.2 (Invitrogen), followed by incubation at room temperature for 10 min to form Cas9 ribonucleoproteins (RNPs). To ablate *SIRT2* or *LCK* from primary human and/or mouse T cells, 2 × 10^6^ activated CD3^+^ T cells were resuspended in 100 µl buffer T (Neon Transfection System; Thermo Fisher) and 15 µl of the Cas9 RNPs were added to the resuspended cells and electroporation was performed at 1,350 V, 10 ms, three cycles. T cells were then cultured in X-VIVO 20 (LONZA) supplemented with 20% FBS with 500 U ml^−1^ IL-2. Ablation of *SIRT2* or *LCK* from Jurkat cells was performed similarly; however, 2 × 10^6^ cells were resuspended in buffer R, and the electroporation was performed at 1,400 V, 10 ms for three cycles. ATTO 550-positive cells were sorted 3 days post-electroporation and the loss of SIRT2 or LCK was confirmed by immunoblot, 2 weeks later.

### Lentivector transduction

Enriched CD3^+^ T cells were stimulated for 24 h in anti-CD3 Abs-coated plates (5 μg ml^−1^, 145-2C11, BioXCell). Freshly concentrated lentivectors were spun-inoculated into activated T cells or Jurkat cells supplemented with Polybrene (6 mg ml^−1^, Sigma) at 2,000 rpm, 32 °C for 2 h.

### L-Selectin shedding assay

WT versus *Sirt2*^−/−^ CD3^+^ T cells were purified from mouse spleens and then surface stained with anti-CD62L. Stained cells remained unstimulated or stimulated with anti-CD3-biotin Abs (0.5 μg ml^−1^; clone 145-2C11, BD Biosciences) for 20 min at room temperature, followed by anti-CD3 crosslinking with 2.5 μg ml^−1^ streptavidin (Thermo Fisher Scientific) for 90 min. CD62L shedding was determined by comparing L-selectin expression on WT versus *Sirt2*^−/−^ T cells.

### In vivo trafficking experiments

CD3^+^ T cells were purified from spleen of WT and *Sirt2*^−/−^ mice. *Sirt2*^−/−^ CD3^+^ T cells were labeled with 2 μM of CellTrace CFSE (Thermo Fisher Scientific) and WT CD3^+^ T cells were labeled with 1 μM CTV (Thermo Fisher Scientific) for 30 min at room temperature. In a parallel experiment, dye assignments were reversed to exclude potential dye-related artifacts. Labeled T cells remained unstimulated or stimulated with 0.5 μg ml^−1^ anti-CD3-biotin Abs (Clone 145-2C11, BD Biosciences) for 20 min on ice, followed by anti-CD3 crosslinking with 2.5 μg ml^−1^ streptavidin (Thermo Fisher Scientific) for 1 h at 37 °C CO_2_ incubator. WT and *Sirt2*^−/−^ T cells were then washed twice, counted, and mixed at 1:1 ratio of CFSE:CTV-labeled cells. Then, 10 × 10^6^ 1:1 ratio CFSE:CTV-labeled cells were injected i.v. into C57BL/6 recipient mice. After 1 h, the recipient mice were killed and LNs, spleens and heparinized blood were collected for downstream flow cytometry analysis.

### Adoptive cell transfer

A total of 5 × 10^6^ purified CD45.2^+^CD8^+^ PMEL T cells or CD45.2^+^CD4^+^ OT-II T cells from WT or *Sirt2*^*−*/*−*^ mice were labeled with CTV (Thermo Fisher Scientific) and i.v. transferred into CD45.1^+^ recipient mice. At 1 week later, mice were vaccinated s.c. with 0.5 × 10^6^ dendritic cells (DCs) pulsed with either gp100_25–33_ peptide (Anaspec, PMEL model) or OVA_323–339_ peptide (InvivoGen, OT-II model). On days 3 and 5 post-immunization, spleens and LNs were collected and T cell proliferation and activation were assessed by flow cytometry.

In the ConA-induced hepatitis model, 5 × 10^6^ purified CD3^+^ T cells from WT or *Sirt2*^−/−^ C57BL/6 mice were i.v. transferred into *Rag2*^−/−^ recipient mice 1 day before ConA administration. A control group of *Rag2*^−/−^ mice did not receive T cells.

### In vitro generation of DCs

BM cells were isolated from the femurs of C57BL/6 mice and cultured at 1 × 10^6^ cells ml^−1^ in complete RPMI-1640 medium supplemented with 20 ng ml^−1^ murine granulocyte–macrophage colony-stimulating factor and 10 ng ml^−1^ murine IL-4 (both from PeproTech) for 7 days. Fresh cytokine-supplemented medium was added on days 3 and 6. On day 7, BM-derived DCs were activated with lipopolysacharide (LPS; 20 ng ml^−1^; Thermo Fisher Scientific). On day 8, nonadherent and loosely adherent cells were collected, washed and pulsed for 4 h at 37 °C with either gp100_25–33_ peptide (1 μg ml^−1^, Anaspec), OVA peptide (10 μg ml^−1^, InvivoGen) or left unloaded as a control. After peptide loading, cells were washed three times with PBS before injection. DC purity, maturation and activation status were confirmed by flow cytometry.

### Mixed BMCs

To generate mixed BMCs, bone-marrow cells from *Sirt2*^*−*/*−*^ CD45.2^+^ donor mice were mixed at a 1:1 ratio with BM cells from WT CD45.1^+^ mice. Then, 0.5 × 10^6^ total mixed BM cells were injected i.v. into 800Gy-irradiated C57BL/6xC57BL/6SJL F1 recipient mice. Chimeras were rested for at least 8 weeks to allow for BM reconstitution.

### Flow cytometry

For surface marker analysis, mouse T cells were stained in PBS containing 2% BSA (FACS buffer) with the following Abs: CD3 (APC or BUV395, clone 145-2C11), CD4 (BUV805 or BV785, clone GK1.5), CD8 (Alexa Fluor 700, 53-6.7), CD44 (Alexa Fluor 488, IM7), CD25 (BV510 or BV711), CD62L (PE-Cy7, MEL-14), CD69 (PE-CF594, H1.2F3), TCRβ (PE), CD5 (BV605), CD24 (BV711), CD45.2 (BV650) CD45.1 (BV510), TCR Vβ5.1/5.2 (PE), TCR Vβ13 (PE) and NK1.1 (BV605). To exclude non-T cells, a dump channel was used consisting of B220, CD19, CD11b, Gr-1 and TER119 all conjugated to APC-Cy7. Cells were incubated at 4 °C for 20 min to 1 h, washed twice with FACS buffer and fixed in PBS containing 1% paraformaldehyde. Dead cells were excluded using the Zombie Violet or Zombie NIR Fixable Viability kit (BioLegend), following the manufacturer’s instructions.

For human T cells staining the following Abs were used: CD8 (BUV395), CD3 (BUV496), CD4 (BUV737) and CD45RA (FITC) from BD Biosciences.

For cytokine intracellular staining, cells were first re-stimulated with phorbol 12-myristate 13-acetate (10 ng ml^−1^, Sigma-Aldrich) and ionomycin (1 μM, Sigma-Aldrich) for 1 h, followed by GolgiPlug treatment (1‰, BD Biosciences) for an additional 6 h. Cells were then surface stained before fixation/permeabilization using the Cytofix/Cytoperm kit (BD Biosciences), followed by intracellular staining with the following Abs: TNF (PE-Cy7, BioLegend), IFN-γ (BB700 or BV711, BD Biosciences), IL-2 (APC), IL-4 (BV605), granzyme B (PE or APC, BD Biosciences) and perforin (Alexa Fluor 647 or BV605, BioLegend).

For FoxP3 intracellular staining, cells were processed using the eBioscience Foxp3/Transcription Factor Staining Buffer Set (Thermo Fisher Scientific) according to the manufacturer’s instructions. Intracellular staining was performed with FoxP3 (PE, BioLegend).

For Nur77 intracellular staining, cells were processed using the BD Pharmingen Transcription Factor Buffer Set (BD Biosciences) according to the manufacturer’s instructions. Intracellular staining was performed with Nur77 (PE or AF647, BioLegend).

For proximal TCR signaling analysis, cells were surface stained before fixation/permeabilization with Cytofix/Cytoperm kit (BD Biosciences), followed by staining with LCK Phospho Tyr394 (PE, BioLegend), LCK Phospho Tyr505 (PerCP-eFluor 710, Thermo Fisher Scientific), Phospho-ERK1/2 Thr202/Tyr204 (APC, Thermo Fisher Scientific), Phospho-ZAP70 (Tyr319/Tyr352, APC, eBioscience) and Phospho-ZAP70 (Tyr493, FITC, eBioscience).

For BMC experiments, BM, thymus, spleen and LN single-cell suspensions were stained in FACS buffer with the following Abs: CD3 (APC, clone 145-2C11), TCRβ (PE), CD4 (BV785, GK1.5), CD8 (Alexa Fluor 700, 53-6.7), CD45.2 (BV421), CD45.1 (BV510), CD69 (PE-CF594, H1.2F3), CD5 (BV605), CD24 (BV711), CD44 (Alexa Fluor 488, IM7), CD25 (PE-Cy5), CD62L (PE-Cy7, MEL-14), c-Kit (BV650) and Sca-1 (AF647). Dump channel Abs included CD19, B220, CD11b, Gr-1, TER119 and NK1.1, all conjugated to APC-Cy7. Dead cells were excluded using the Zombie NIR Fixable Viability Kit (BioLegend).

For assessing DC maturation and activation, single-cell suspensions were stained in FACS buffer with the following Abs: CD11b (BV605), CD11c (Alexa Fluor 488), CD86 (BV510), MHC class II IA/IE (Alexa Fluor 647) and F4/80 (BV711). T cells were excluded using CD3 (APC). A dump channel was used to exclude non-DC populations, consisting of CD19, B220, Gr-1, TER119 and NK1.1, all conjugated to APC-Cy7. Dead cells were excluded using Zombie Violet Fixable Viability Dye (BioLegend).

Cells were acquired on a BD FACSymphony A5, LSR II (Becton Dickinson), and Cytek (Biosciences) and data were analyzed with FlowJo v.10.0 software.

### Ca^2+^ flux assay

In brief, 2 × 10^6^ T cells were washed twice with PBS and loaded with 1 μM Indo-1 AM Ca^2+^ indicator dye (Thermo Fisher Scientific) at 37 °C for 30 min in 1 ml DMEM. T cells were washed again twice with PBS and incubated for additional 30 min at 37 °C in 1 ml complete medium. After washing, cells were surface stained, then incubated with the indicated concentration of biotinylated anti-CD3e monoclonal antibody (0.015–10 μg ml^−1^) for 20 min at 4 °C. Indo-1 fluorescence ratios were recorded for 30 s or 60 s to obtain the baseline relative Ca^2+^ levels, followed by the addition of streptavidin (2.5 μg ml^−1^, Thermo Fisher Scientific) at 90 s or 120 s and this was followed by the addition of 1 µM ionomycin (Thermo Fisher Scientific) at 360 s.

In a similar experimental setting, EL4 cells were loaded with gp100 peptide or left unloaded (control cells) and CD8^+^ PMEL T cells were loaded with Indo-1 AM. Indo-1 fluorescence ratios were recorded for 60 s to obtain the baseline relative Ca^2+^ levels, followed by the addition of gp100-loaded EL4 at 120 s and this was followed by the addition of 1 µM ionomycin (Thermo Fisher Scientific) at 480 s.

### Ca^2+^ live imaging

Freshly prepared OT-II T cells loaded with Ca^2+^ indicator Fluo-4-AM (Thermo Fisher Scientific) and Ova-pulsed B cells (APCs) labeled with Cell Tracker Orange CMRA Dye (Thermo Fisher Scientific) were mixed 1:1 and were then immediately loaded onto a TomoDish (Tomocube). Samples on a TomoDish were placed on a TomoChamber (Tomocube) to maintain CO_2_ (5%) and temperature (37 °C) levels. Cells were subjected to time-lapse imaging with an objective and a condenser lens, UPLASAPO ×60 W 1.2 NA lens (Olympus), on a holotomographic microscope, HT-2 (Tomocube). Holographic images were generated from interfered images at the camera plane between the two split 532 nm laser beams of a reference beam and a sample illuminated beam obtained at various incident angles modulated by a high-speed illumination scanner using a digital micromirror device. Following the holographic imaging acquisition in 400 ms, single z-plane fluorescent images of Fluo-4 AM for T cells and the Cell Tracker Orange CMRA for B cells illuminated with an LED light source (470 and 570 nm, respectively) were sequentially acquired with 100-ms exposure time. The refractive index distribution was reconstructed and visualized for the three-dimensional ODT images and the trace of Fluo-4 AM fluorescence intensities was determined using TomoAnalysis software (Tomocube v.1.5).

### Confocal imaging

After isolation from mouse spleens, WT and *Sirt2*^*−*/*−*^ T cells were activated with anti-mouse CD3ε Abs-coated 24-well plates (5 μg ml^−1^, 145-2C11, InVivoMab) for the indicated time periods. T cells were then fixed with paraformaldehyde (4%, 15 min, 37 °C) and permeabilized with 0.2% Triton X-100 in PBS for 5 min. Following a blocking step in blocking buffer (0.1% Triton X-100, 10% donkey serum, 60 min, room temperature), T cells were incubated with NFATc2 mouse Abs (Invitrogen, cat. MA1-025, Clone no. 25A10.06.02; 1:500 dilution) and AP-1 rabbit Abs (PeproTech; cat. no. 22114-1 AP; 1:250 dilution) in PBS with 2% normal goat serum overnight at 4 °C. After washing, T cells were labeled with secondary Abs goat anti-mouse Alexa Fluor 488 (Invitrogen, cat. no. A28175 1:500 dilution) and goat anti-rabbit Alexa Fluor 647 (Invitrogen, cat. no. A21245; 1:500 dilution) in 0.1% Triton/PBS for 1 h. T cells were then mounted in mounting medium with DAPI (4′,6′-diamidino-2-phenylindole; Vector Laboratories) and analyzed using a Carl Zeiss LSM confocal microscopy.

Quantification of nuclear NFATc2 and AP-1 signal was performed using ImageJ software by gating the nucleus. Mean fluorescence intensity values for NFATc2 and AP-1 within the nucleus are reported. The colocalization index was calculated using Pearson’s correlation coefficient (*r*), where values ranged from –1 (no colocalization) to +1 (perfect colocalization).

### RNA isolation and reverse transcription quantitative PCR

Total RNAs were extracted from cells using the RNeasy Mini kit (QIAGEN) according to manufacturer’s instructions. Following extraction, 1 μg of total RNAs was reverse transcribed using the iScript reverse transcription kit (Bio-Rad). Quantitative PCR of the indicated genes were performed using SsoAdvanced Universal SYBR Green Supermix (Bio-Rad) in the CFX Connect Real-Time System (Bio-Rad). Sense and antisense primers for mouse *Nr4a1*, mouse *Actb*, human *NR4A1* and human ACTB were predesigned by Bio-Rad and provided as a mixture, PrimePCR SYBR Green Assay.

### Immunoprecipitation

For IP assays, activated CD3^+^ T cells were lysed in IP lysis buffer (20 mM HEPES, pH 7.9, 180 mM KCl, 0.2 mM EDTA, 1.5 mM MgCl_2_, 20% glycerol and 0.1% Nonidet P-40, containing a mixture of protease inhibitors). Cell lysates were incubated overnight at 4 °C with specific Abs against acetyl-lysine (9441, 1:50 dilution) from Cell Signaling, SIRT2 (ab211033, 1:50 dilution) and LCK (ab227975, 1:50 dilution) from Abcam. Rabbit monoclonal Abs IgG (Cell Signaling, 3900) was used as isotype control. After addition of anti-rabbit Ig agarose-beads (TrueBlot, Rockland), samples were incubated at 4 °C for 2 h. Beads were washed five times with IP lysis buffer and proteins were released from the beads by boiling in 3× SDS sample loading buffer and loaded into 10 % SDS–PAGE gel for immunoblot analysis.

### Subcellular protein extraction

Cell extracts from WT and *Sirt2*^*−*/*−*^ T cells were fractionated by the ProteoExtract Subcellular Proteome Extraction kit (Millipore Sigma) according to the manufacturer’s instructions.

### Immunoblotting analysis

Whole cell lysates were prepared using lysis buffer (Pierce RIPA buffer, Thermo Fisher Scientific) supplemented with cOmplete protease inhibitor cocktail and PhosSTOP phosphatase inhibitor cocktail (Roche Applied Science).

Cell lysates (20 µg) or IP samples were loaded onto 10% SDS–PAGE and separated by electrophoresis followed by semi-dry transfer into polyvinylidene fluoride membranes (Immun-Blot PVDF membrane, Bio-Rad) using Trans-Blot Turbo transfer system (Bio-Rad). After transfer, the membranes were blocked at RT with Tris-buffered saline containing 0.05% Tween-20 (TBST) and 5% nonfat dry milk for 1 h and then incubated overnight at 4 °C with specific primary Abs (indicated below). The membranes were washed three times with TBST and then incubated for 1 h with horseradish peroxidase (HRP)-conjugated goat anti-mouse IgG H&L (Abcam, no. ab97051, 1:3,000 dilution) for regular immunoblot analysis, or HRP-conjugated mouse anti-rabbit IgG light-chain specific (Cell Signaling, 93702, 1:1,000 dilution) for IP samples. After washing three times with TBST, bound Abs were detected by chemiluminescence using the Pierce ECL Western Blotting Substrate and the Super Signal West Femto Maximum Sensitivity Substrate kits (Thermo Fisher Scientific). Image acquisition was performed with the Amersham imager 600 system (GE Healthcare Bio-Sciences).

Immunoblotting was performed using primary Abs against acetyl-lysine (9441, 1:1,000 dilution), SIRT2 (12650, 1:1,000 dilution), LCK (2752, 1:1,000 dilution), ZAP70 (3165, 1:1,000 dilution) LAT (45533, 1:1,000 dilution) SLP-76 (25361, 1:1,000 dilution), PLCγ1 (5690, 1:1,000 dilution), Erk1/2 (9102, 1:1,000 dilution), GFP (2956, 1:1,000 dilution), Phospho-LAT (Tyr220) (20172, 1:1,000 dilution), Phospho-PLCγ1 (Tyr783) (14008, 1:1,000 dilution), Phospho-SLP-76 (Ser376) (14745, 1:1,000 dilution), Phospho-ZAP70 (Tyr319)/Syk (Tyr352) (2717, 1:1,000 dilution), Phospho-Src Family (Tyr416) (6943, 1:1,000 dilution), phospho-LCK (Tyr505) (37458, 1:1,000 dilution) and Phospho-p44/42 MAPK (Erk1/2) (Thr202/Tyr204) (4370, 1:1,000 dilution) from Cell Signaling, β-actin (ab8227, 1:5,000 dilution) and GAPDH (ab181602, 1:10,000 dilution) from Abcam.

### Pulldown polyHis protein–protein interaction assay

Human histidine-LCK fusion protein was manufactured, immobilized on HisPur cobalt resin (Thermo Fisher Scientific) and used as a bait protein. Human SIRT2 protein was manufactured and used as prey proteins. The pulldown assay was performed following the manufacturer’s protocol (Pierce Pulldown PolyHis Protein:Protein Interaction kit, Thermo Fisher Scientific). A nontreated gel control (minus bait, plus prey) and the immobilized bait control (plus bait, minus prey) were included. The eluted proteins were subjected to SDS–PAGE (4–12% gel) and transferred to PVDF membranes for immunoblotting analysis. Immunoblotting was performed using primary Abs against SIRT2 (12650, 1:1,000 dilution), LCK (2752, 1:1,000 dilution) and His-tag (2365, 1:1,000 dilution) from Cell Signaling.

### Pulldown GST protein–protein interaction assay

The coding region of the LCK-SH3 domain (183 bp) was cloned into the pcDNA3.1(+)-N-GST vector (GenScript). The resulting vector expresses a fusion protein of LCK-SH3 with GST at the N terminus. Similarly, the coding region of the LCK-LR domain (60 bp), including the WT sequence or mutants K228R and K228Q, was cloned into the pcDNA3.1(+)-N-eGFP vector (GenScript). The resultant vectors express fusion proteins of LCK-LR whether WT or mutants with GFP at the N terminus.

Each vector was transfected into 293T cells using Lipofectamine 3000 Transfection Reagent (Thermo Fisher Scientific). The 293T cell lysates expressing LCK-SH3-GST fusion proteins were applied to glutathione resin (GenScript). The glutathione resin, bound with SH3-GST, was washed five times before the introduction of 293T cell lysates expressing either Empty or LCK-LR (WT, K228R or K228Q mutants) GFP fusion proteins. After another five rounds of washing, the bound SH3-GST/LR-GFP proteins were eluted with 10 mmol l^−1^ of reduced glutathione. The eluted proteins were subjected to 4–12% SDS–PAGE gel and transferred to PVDF membranes for immunoblotting analysis. Immunoblotting was performed using primary Abs against GFP (2956S, 1:1,000 dilution) and GST-Tag (2625, 1:1,000 dilution) from Cell Signaling.

### Structural modeling using AlphaFold

Structural predictions were performed using AlphaFold (v.2.3.1)^33^ to investigate LCK-LR binding to the LCK-SH3 domain. Modeling included the following peptides: unacetylated K228 LCK-LR, acetylated K228 LCK-LR and the K228R and K228Q mutant LCK-LR peptides. Structural comparison and visual inspection were performed using PyMOL (Schrödinger) for figure preparation and structural interpretation.

### Fluorescence polarization assays

The FP experiments were performed in 384-well black plates (Thermo Fisher Scientific) and the sample signals were read by a Synergy 2 plate reader (Biotek). The polarization was measured at room temperature with an excitation wavelength at 485 nm and an emission wavelength at 535 nm. All FP experiments were performed in an assay buffer of 25 mM HEPES (pH 7.4), 100 mM NaCl, 0.01% Triton X-100 and 100 µg ml^−1^ *γ*-globulin. The final reaction volume was set to 15 µl.

In the FP saturation experiments investigating interactions between LCK-SH3-GST and LCK-LR peptides, four peptide variants were tested: acetylated K228, deacetylated K228, K228R and K228Q. The concentration of the fluorescently labeled LCK-LR tracer was fixed at 10 nM in assay buffer, while LCK-SH3-GST was titrated across a concentration range of 0 to 160 µM. After combining each peptide variant with the indicated concentrations of LCK-SH3-GST, assay plates were incubated for 3 h at room temperature in the dark on an orbital shaker before measurement of FP signals.

To assess nonspecific interactions, LCK-LR peptides were also combined with empty-GST proteins at various concentrations under the same conditions before recording the polarization signals.

The data were analyzed by nonlinear least-square analyses using GraphPad Prism v.8.0 to derive the *K*_d_ value. Each experiment was repeated three times. The results were expressed as mean ± s.e.m.

### HPLC-based SIRT2 deacetylase activity assay

The deacetylation activity of SIRT2 was assessed by high-performance liquid chromatography (HPLC). Acetylated K228 LCK-LR peptides (32 µM) were incubated with or without purified SIRT2 (0.2 µM) in the reaction buffer (20 mM Tris, pH 8.0, 1 mM dithiothreitol (DTT) and 1 mM NAD^+^) at 37 °C. To quench the reactions, acetonitrile was added into the reaction mixture. After centrifuging at 10,000*g* for 10 min, the supernatant was collected and lyophilized to concentrate the samples. Then, 50 µl of biological water was added to each sample which was then analyzed by HPLC on a Luna C18(2) column (100 A, 250 × 4.6 mm, 5 µm Phenomenex).

Solvents used for HPLC were water with 0.1% trifluoroacetic acid (solvent A) and acetonitrile (solvent B). The gradient for HPLC condition was 5 % B for 2 min, 5—20 % B in 2 min, 20–40% B in 14 min, 40–95% B in 3 min, 95% B for 4 min, 95—5% B in 4 mins then 5% B for 1 min. The flow rate was 0.75 ml min^−1^. UV-Vis detector was set to measure at wavelength 280 nm. To locate the product, substrate and NAD^+^ peaks, a sample with the standard product, substrate peptide and NAD^+^ was ran under the same chromatographic gradient conditions.

### RNA sequencing analysis

RNA sequencing analysis was performed at the Molecular Genomics Core Facility of H. Lee Moffitt Cancer Center.

CD4^+^ and CD8^+^ TILs were isolated from B16F10 s.c. tumors from WT and *Sirt2*^−/−^mice (*n* = 4 per group). Total RNAs were extracted using the RNeasy Micro kit (QIAGEN; cat. no. 74004) according to the manufacturer’s instructions. Extracted RNAs were quantitated with the Qubit Fluorometer (Thermo Fisher Scientific) and screened for quality on the Agilent TapeStation 4200 using the high-sensitivity RNA ScreenTape (Agilent Technologies). The samples were then processed for RNA sequencing using the Takara SMARTer Stranded Total RNA-seq Kit v.2 Pico Input Mammalian kit (Takara Bio USA). Briefly, 1 ng of RNA was used to generate cDNA and a strand-specific ribosomal RNA-depleted library following the manufacturer’s protocol. Quality control steps were performed, including TapeStation size assessment and quantification using the Kapa Library Quantification kit (Roche). The final libraries were then normalized, denatured and sequenced on the Illumina NextSeq 2000 sequencer with the P3-200 cycle reagent kit to generate at least 60 million 100-base read pairs per sample (Illumina).

### T cell receptor sequencing

CD8^+^ SP thymocytes were isolated from WT and *Sirt2*^−/−^ mouse thymi. Total DNA was extracted using DNeasy Blood & Tissue Kit (QIAGEN). Amplification and sequencing of the TCR β complementarity-determining region 3 (CDR3) sequences were performed using the ImmunoSEQ platform (Adaptive Biotechnologies)^52,53^. Clonality scores based on Shannon’s entropy were calculated using the ImmunoSEQ Analyzer software v.3.0 (https://clients.adaptivebiotech.com/) and reported on a scale of 0 to 1, with 0 and 1 indicating a maximally diverse and completely monoclonal T cell population, respectively.

### LC–MS/MS

LC–MS/MS analysis was performed at the Proteomics Core Facility of H. Lee Moffitt Cancer Center.

For the identification of LCK acetylated-lysine sites, WT and *Sirt2*^−/−^ preactivated murine CD3^+^ T cells, CD4^+^ OT-II T_M_-like cells, B16F10 TILs and human Jurkat cells were washed twice with PBS and lysed in the IP lysis buffer (Thermo Fisher Scientific). IP assay was performed as described previously using Anti-Lck Abs (Abcam).

IP beads were resuspended in 20 µl of SDS–PAGE MOPS running buffer (Bio-Rad, cat. no. 1610788), 10 µl of loading buffer (Bio-Rad, cat. no. 1610791) and 2 µl of reducing agent (Bio-Rad, cat. no. 1610792), then boiled and denatured at 95 °C for 5 min. Samples were cooled and loaded onto a 10% Bis–Tris Criterion XT Precast Gel (Bio-Rad). SDS–PAGE was performed at 125 V for 90 min. The gel bands were then stained with Coomassie Brilliant Blue, imaged and cut. An in-gel digestion was performed with TCEP reduction (2 mM) and IAA alkylation (20 mM) followed by digestion with 200 ng of trypsin overnight. Then, 200 ng more of trypsin was added the next day for an additional 2-h digest. Peptides were extracted from the gel pieces using 50% acetonitrile, 0.1% trifluoroacetic acid, then dried down in a vacuum centrifuge. Peptides were resuspended in 200 µl of 0.1% trifluoroacetic acid for C18 desalting using a Thermo SOLAu plate. Eluted, desalted peptides were dried down in a vacuum centrifuge before being resuspended in 20 µl of 2% acetonitrile, 0.1% formic acid for MS analysis.

A nanoflow ultra high-performance liquid chromatograph (RSLC, Dionex) coupled to an electrospray bench top orbitrap mass spectrometer (Q-Exactive plus, Thermo) was used for tandem mass spectrometry peptide sequencing experiments. The sample was first loaded onto a pre-column (2 cm × 100 µm ID packed with C18 reversed-phase resin, 5 µm, 100 Å) and washed for 8 min with aqueous 2% acetonitrile and 0.04% trifluoroacetic acid. The trapped peptides were eluted onto the analytical column, (C18, 75 µm ID x 25 cm, 2 µm, 100 Å, Dionex). The 120-min gradient was programmed as 95% solvent A (2% acetonitrile + 0.1% formic acid) for 8 min, solvent B (90% acetonitrile + 0.1% formic acid) from 5% to 38.5% in 90 min, then solvent B from 50% to 90% B in 7 min and held at 90% for 5 min, followed by solvent B from 90% to 5% in 1 min and re-equilibrate for 10 min. The flow rate on analytical column was 300 nl min^−1^. Spray voltage was 1,900 V. Capillary temperature was 275 °C. S lens RF level was set at 50. Data-dependent acquisition was performed using top 16 precursors. The resolutions for MS and MS/MS were set at 70,000 and 17,500 respectively. Dynamic exclusion was 15 s for previously sampled peaks.

Raw files were loaded into Proteome Discoverer v.3.0 for database searching with Mascot and Sequest. Scaffold v.5.0 and Skyline v.23.1 were used to visualize the data.

### LCK sequence alignment

Sequences of LCK orthologs from various species were found by search using the NCBI nucleotide database. Multiple Sequence Alignment was performed on Clustal Omega program at https://www.ebi.ac.uk/Tools/msa/clustalo/ ref. ^54^.

### Enrichment analysis

For gene set enrichment analysis, the list of upregulated genes in *Sirt2*^*−*/*−*^ versus WT TILs was uploaded to MSigDB^55^ and the overlap with the HALLMARK gene sets was calculated^56^. A false discovery rate *q*-value < 0.1 was used as a cutoff.

### Quantification and statistical analysis

Statistical analyses were performed with Prism software v.7.01 (GraphPad Software) using a two-tailed unpaired or paired Student’s *t*-test, Dunn’s test, chi-squared test and a one- or two-way ANOVA. In all cases, statistical significance was considered when *P* < 0.05. Error bars show mean ± s.e.m. and *P* values were represented as *P < 0.05, **P < 0.01, ***P < 0.001 and ****P < 0.0001.

### Reporting summary

Further information on research design is available in the Nature Portfolio Reporting Summary linked to this article.

## Online content

Any methods, additional references, Nature Portfolio reporting summaries, source data, extended data, supplementary information, acknowledgements, peer review information; details of author contributions and competing interests; and statements of data and code availability are available at 10.1038/s41590-025-02377-3.

## Supplementary information

Reporting Summary

## Source data

Source Data Figs. 1–5 and Extended Data Figs. 1–10 Statistical source data.

Source Data Figs. 1–3 and 5 and Extended Data Figs. 1, 3–5 and 10 Unprocessed immunoblots.

## Data Availability

The RNA sequencing dataset generated during this study has been submitted in the Gene Expression Omnibus under accession no. GSE265880. The TCR sequencing data have been deposited into the ImmuneACCESS platform at https://clients.adaptivebiotech.com/pub/hamaidi-2024-s. Source data are provided with this paper.

## Code availability

No custom code was used in this study. All analyses were performed using standard software packages as described in Methods.
