# Supplementary material for: SIRT2-mediated deacetylation of LCK governs the magnitude of T cell receptor signaling
Source: Nat Immunol. 2026 Jan 29;27(2):213–24. doi: 10.1038/s41590-025-02377-3 (PMC12864039; doi:10.1038/s41590-025-02377-3)
Supplement: Supplementary file 1 — Reporting Summary [file 41590_2025_2377_MOESM1_ESM.pdf]

nature portfolio

Corresponding author(s): Sungjune Kim

Last updated by author(s): Oct 31, 2025

Reporting Summary

Nature Portfolio wishes to improve the reproducibility of the work that we publish. This form provides structure for consistency and transparency in reporting. For further information on Nature Portfolio policies, see our Editorial Policies and the Editorial Policy Checklist.

Statistics

For all statistical analyses, confirm that the following items are present in the figure legend, table legend, main text, or Methods section.

n/a	Confirmed	
☐	☒	The exact sample size (*n*) for each experimental group/condition, given as a discrete number and unit of measurement
☐	☒	A statement on whether measurements were taken from distinct samples or whether the same sample was measured repeatedly
☐	☒	The statistical test(s) used AND whether they are one- or two-sided<br>*Only common tests should be described solely by name; describe more complex techniques in the Methods section.*
☐	☒	A description of all covariates tested
☐	☒	A description of any assumptions or corrections, such as tests of normality and adjustment for multiple comparisons
☐	☒	A full description of the statistical parameters including central tendency (e.g. means) or other basic estimates (e.g. regression coefficient) AND variation (e.g. standard deviation) or associated estimates of uncertainty (e.g. confidence intervals)
☐	☒	For null hypothesis testing, the test statistic (e.g. *F*, *t*, *r*) with confidence intervals, effect sizes, degrees of freedom and *P* value noted<br>*Give P values as exact values whenever suitable.*
☒	☐	For Bayesian analysis, information on the choice of priors and Markov chain Monte Carlo settings
☒	☐	For hierarchical and complex designs, identification of the appropriate level for tests and full reporting of outcomes
☐	☒	Estimates of effect sizes (e.g. Cohen's *d*, Pearson's *r*), indicating how they were calculated

Our web collection on statistics for biologists contains articles on many of the points above.

Software and code

Policy information about availability of computer code

Data collection	• Flow cytometry data were acquired using BD FACSymphony™ A5, BD LSR II (Becton Dickinson), and Cytek® Aurora (Cytek Biosciences) instruments. BD FASC Diva™ software version 8 and Cytek SpectroFlo v3.2.1 were used to collect data from flow cytometry.<br>• Bulk RNA-seq libraries were sequenced on an Illumina NextSeq 2000 sequencer (Illumina, San Diego, CA).<br>• Confocal microscopy images were acquired using a Zeiss LSM 880 confocal microscope (Carl Zeiss AG, Oberkochen, Germany).<br>• qRT-PCR was performed using the CFX Connect™ Real-Time PCR System (Bio-Rad).<br>• Histology slides were scanned using the Aperio AT2 digital whole-slide scanner (Leica Biosystems).<br>• Fluorescence and absorbance measurements were collected using a Synergy™ HTX multi-mode microplate reader (BioTek Instruments).<br>• Calcium imaging was conducted using a holotomographic microscope (HT-2, Tomocube, Republic of Korea).<br>• Western blot and chemiluminescent imaging were performed using the Amersham Imager 600 (GE Healthcare) and Bio-Rad ChemiDoc™ systems.<br>• HPLC-based SIRT2 deacetylase activity assays were performed using a Luna® C18(2) column (Phenomenex) and UV-Vis detection at 280�nm.<br>• TCRβ CDR3 sequencing was performed using the ImmunoSEQ® platform (Adaptive Biotechnologies).<br>• LC-MS/MS analysis was conducted at the Proteomics Core Facility of H. Lee Moffitt Cancer Center. Immunoprecipitated LCK proteins from murine and human T cells were subjected to SDS-PAGE, in-gel digestion, and C18 desalting. Peptides were analyzed on a Dionex RSLC nanoflow UHPLC system coupled to a Q-Exactive Plus Orbitrap mass spectrometer (Thermo Scientific). Data were acquired using Top16 data-dependent acquisition and analyzed using Proteome Discoverer 3.0 (Mascot and Sequest), with visualization in Scaffold 5.0 and Skyline (v23.1).
Data analysis	All software used for data analysis is publicly available unless otherwise stated:<br>• Flow cytometry data were analyzed using FlowJo™ v10 (BD Biosciences).<br>• Statistical analyses and graph generation were performed using GraphPad Prism 10.0 (GraphPad Software).

• Western blot quantification and confocal image intensity measurements were performed using ImageJ v1.54d.
• Histological analysis (areas of hepatic apoptosis and necrosis) was conducted using ImageScope x64 (Leica Biosystems).
• Proteomics data (IP-MS/MS) were analyzed using MASCOT and SEQUEST (commercial software) for protein identification, and MaxQuant v1.2.2.5 (academic software) for identification and quantification of acetyl-lysine–containing peptides.
• Heatmaps were generated using Cluster 3.0 and Java TreeView v1.1.6r4.
• Gene set enrichment analysis (GSEA) of RNA-seq data was performed using MSigDB (http://software.broadinstitute.org/gsea/msigdb/) with hallmark gene sets.
• TCR clonality scores based on Shannon's entropy were calculated using the ImmunoSEQ Analyzer 3.0 platform (https://clients.adaptivebiotech.com/).
• Multiple sequence alignment was performed using the Clustal Omega online tool (https://www.ebi.ac.uk/Tools/msa/clustalo/).
• Calcium imaging fluorescence intensities were quantified using TomoAnalysis software (Tomocube, Version 1.5).

For manuscripts utilizing custom algorithms or software that are central to the research but not yet described in published literature, software must be made available to editors and reviewers. We strongly encourage code deposition in a community repository (e.g. GitHub). See the Nature Portfolio guidelines for submitting code & software for further information.

Data

Policy information about availability of data

All manuscripts must include a data availability statement. This statement should provide the following information, where applicable:

- Accession codes, unique identifiers, or web links for publicly available datasets
- A description of any restrictions on data availability
- For clinical datasets or third party data, please ensure that the statement adheres to our policy

• The RNA-sequencing dataset generated during this study has been submitted in the Gene Expression Omnibus under the accession number GSE265880. To review the RNA-sequencing data, go to https://www.ncbi.nlm.nih.gov/geo/query/acc.cgi?acc=GSE265880 and enter the token mbcjaewghjwzfuj.
• The TCR sequencing data have been deposited into the ImmuneACCESS platform and can be accessed at https://clients.adaptivebiotech.com/pub/hamaidi-2024-s (DOI: 10.21417/hamaidi2024s).

Research involving human participants, their data, or biological material

Policy information about studies with human participants or human data. See also policy information about sex, gender (identity/presentation), and sexual orientation and race, ethnicity and racism.

Reporting on sex and gender	N/A
Reporting on race, ethnicity, or other socially relevant groupings	N/A
Population characteristics	Human peripheral blood mononuclear cells (PBMCs) were obtained from de-identified adult healthy donors. Buffy coats were purchased from OneBlood.<br><br>Tumor-infiltrating lymphocytes (TILs) were isolated from de-identified tumor biopsies of patients with non-small cell lung cancer (NSCLC) and small cell lung cancer (SCLC). All human samples were fully de-identified prior to analysis in accordance with institutional guidelines and ethical regulations.
Recruitment	None
Ethics oversight	Institutional Review Board (IRB) approval was not required for the use of human peripheral blood mononuclear cell (PBMC) samples from healthy adult donors, as these were purchased de-identified from OneBlood.<br><br>Studies involving the collection and expansion of tumor-infiltrating lymphocytes (TILs) from patients with non-small cell lung cancer (NSCLC) and small cell lung cancer (SCLC) were conducted under approved IRB protocols. All human TIL samples were provided in a fully de-identified manner.

Note that full information on the approval of the study protocol must also be provided in the manuscript.

Field-specific reporting

Please select the one below that is the best fit for your research. If you are not sure, read the appropriate sections before making your selection.

☒ Life sciences ☐ Behavioural & social sciences ☐ Ecological, evolutionary & environmental sciences

For a reference copy of the document with all sections, see nature.com/documents/nr-reporting-summary-flat.pdf

Life sciences study design

All studies must disclose on these points even when the disclosure is negative.

Sample size	No statistical methods were used to predetermine sample size for the experiments reported in this manuscript. All sample sizes are clearly indicated in the corresponding figure legends. Sample sizes were chosen to maximize the likelihood of detecting biologically meaningful differences while maintaining statistical power.<br>For in vivo experiments, efforts were made to minimize unnecessary use of animals. Typically, more than five animals per group were used, based on the availability of transgenic genotypes and age- and sex-matched controls.<br>For in vitro functional assays involving primary mouse T cells and human tumor-infiltrating lymphocytes (TILs), sample sizes were determined based on the availability and yield of viable target cells from each donor or mouse.
Data exclusions	We are not excluding data
Replication	The number of biologically independent replicates for each experiment is indicated in the figure legends. All key experiments were independently repeated at least twice, with sufficient replicates per group to ensure statistical robustness. All attempts at replication were successful and produced consistent results.
Randomization	To study any difference relating to genotype between WT and Sir2KO mice, animals were not intentionally randomized. Mice were allocated to groups based on their genotype with age- and sex-matched.<br>For other different treatments, mice with the same genotype were assigned randomly to experimental and control groups.
Blinding	No blinding method was used. Blinding was not relevant in this study because all data were obtained through automated or quantitative analyses that eliminate observer bias.

Reporting for specific materials, systems and methods

We require information from authors about some types of materials, experimental systems and methods used in many studies. Here, indicate whether each material, system or method listed is relevant to your study. If you are not sure if a list item applies to your research, read the appropriate section before selecting a response.

Materials & experimental systems

n/a	Involved in the study	
☐	☒	Antibodies
☐	☒	Eukaryotic cell lines
☒	☐	Palaeontology and archaeology
☐	☒	Animals and other organisms
☒	☐	Clinical data
☒	☐	Dual use research of concern
☒	☐	Plants

Methods

n/a	Involved in the study	
☒	☐	ChIP-seq
☐	☒	Flow cytometry
☒	☐	MRI-based neuroimaging

Antibodies

Antibodies used

1- Immunoprecipitation
• Antibody, Supplier, #Catalogue No, Clone No. (if available), Dilution
• Anti-SIRT2, Abcam, #ab211033, Clone: EPR20411-105, 1:30
• Anti-LCK, Abcam, #ab227975, Clone: EPR20798-107, 1:30
• Anti-acetyl-lysine, Cell signaling technology, #9441, 1:50
• Rabbit mAb IgG, Cell signaling technology, #3900, Clone: DA1E, 1:50
2- Western blotting analysis
• Antibody, Supplier, #Catalogue No, Clone No. (if available), Dilution
• Anti-acetyl-lysine, Cell signaling technology, #9441, 1:1,000
• Anti-β-Actin, Abcam, #ab8227, 1:5,000
• Anti-GAPDH, Abcam, #ab181602, Clone: EPR16891, 1:10,000
• Goat Anti-Rabbit IgG-HRP H&L, Abcam, #ab97051, 1:3000
• Mouse Anti-Rabbit IgG-HRP (Light-Chain Specific), Cell signaling technology, #93702, Clone: D4W3E, 1:1,000
• Anti-SIRT2, Cell signaling technology, #12650, Clone: D4O5O, 1:1,000
• Anti-LCK, Cell signaling technology, #2752, 1:1,000
• Anti-Zap70, Cell signaling technology, #3165, Clone: D1C10E, 1:1,000
• Anti-LAT, Cell signaling technology, #45533, Clone: E3U6J, 1:1,000
• Anti-SLP-76, Cell signaling technology, #25361, Clone: E4N7E, 1:1,000
• Anti-PLCγ1, Cell signaling technology, #5690, Clone: D9H10, 1:1,000
• Anti-p44/42 MAPK (Erk1/2), Cell signaling technology, #9102, 1:1,000
• Anti-GFP, Cell signaling technology, #2956, Clone: D5.1, 1:1,000
• Anti-Phospho-LAT (Tyr220), Cell signaling technology, #20172, Clone: E3S5L, 1:1,000
• Anti-Phospho-PLCγ1 (Tyr783), Cell signaling technology, #14008, Clone: D6M9S, 1:1,000
• Anti-Phospho-SLP-76 (Ser376), Cell signaling technology, #14745, Clone: D9D6E, 1:1,000

• Anti-Phospho-Zap-70 (Tyr319)/Syk (Tyr352), Cell signaling technology, #2717, Clone: 65E4, 1:1,000
• Anti-Phospho-Src Family (Tyr416), Cell signaling technology, #6943, Clone: D49G4, 1:1,000
• Anti-Phospho-Lck (Tyr505), Cell signaling technology, #37458, Clone: E3Z5E, 1:1,000
• Anti-Phospho-p44/42 MAPK (Erk1/2) (Thr202/Tyr204), Cell signaling technology, #4370, Clone: D13.14.4E, 1:1,000
3- Flow cytometry
• Antibody (Color), Supplier, #Catalogue No. Clone No., Dilution

• Anti-CD3 (BUV395), BD Biosciences, #563565, Clone: 145-2C11, 1:50
• Anti-CD3 (APC), Biolegend, #100312, Clone: 145-2C11, 1:50
• Anti-CD4 (BUV805), BD Biosciences, #564922, Clone: GK1.5, 1:200
• Anti-CD4 (BV785), Biolegend, #100453, Clone: GK1.5, 1:50
• Anti-CD8 (Alexa Fluor 700), Biolegend, #100730 Clone: 53-6.7, 1:200
• Anti-CD44 (Alexa Fluor 488), Biolegend, #103016, Clone: IM7, 1:200
• Anti-CD25 (BV510), Biolegend, #102042, Clone: PC61, 1:50
• Anti-CD25 (BV711), Biolegend, #102049, Clone: PC61, 1:50
• Anti-CD25 (PE/Cyanine5), Biolegend, #102010, Clone: PC61, 1:50
• Anti-CD62L (PE-Cy7), Biolegend, #104418, Clone: MEL-14, 1:200
• Anti-CD69 (PE-CF594) BD Biosciences, #562455 Clone: H1.2F3, 1:50
• Anti-TCR Vβ5.1, 5.2 (PE), Biolegend, #139504, Clone: MR9-4, 1:50
• Anti-TCR Vβ13 (PE), Biolegend, #140704, Clone: MR12-4, 1:50
• Anti-TCR β (PE) Biolegend, #109208, Clone: H57-597, 1:50
• Anti-CD45.2 (Brilliant Violet 420), Biolegend, #109831, Clone: 104, 1:50
• Anti-CD45.2 (Brilliant Violet 650), Biolegend, #109836, Clone: 104, 1:50
• Anti-CD45.1 (Brilliant Violet 510), Biolegend, #110741, Clone: A20, 1:50
• Anti-NK-1.1 (APC/Cyanine7), Biolegend, #156510, Clone: S17016D, 1:50
• Anti-NK-1.1 (Brilliant Violet 605), Biolegend, #156547, Clone: S17016D, 1:50
• Anti-CD11b (APC/Cyanine7), Biolegend, #101226, Clone: M1/70, 1:50
• Anti-Ly-6G/Ly-6C (Gr-1) (APC/Cyanine7), Biolegend, #108424, Clone: RB6-8C5, 1:50
• Anti-CD19 (APC/Cyanine7), Biolegend, #152412, Clone: 1D3/CD19, 1:50
• Anti-B220 (APC/Cyanine7), Biolegend, #103224, Clone: RA3-6B2, 1:50
• Anti-TNF-α (PE/Cyanine7), Biolegend, #506324, Clone: MP6-XT22, 1:50
• Anti-CD24 (Brilliant Violet 711), Biolegend, #101851, Clone: M1/69, 1:50
• Anti-CD5 (Brilliant Violet 605), Biolegend, #100651, Clone: 53-7.3, 1:50
• Anti-IFN-γ (Brilliant Violet 711), Biolegend, #505836, Clone: XMG1.2, 1:50
• Anti-IL-2 (APC), Biolegend, #503810, Clone: JES6-5H4, 1:50
• Anti-IL-4 (Brilliant Violet 605), Biolegend, #504126, Clone: 11B11, 1:50
• Anti-Granzyme B (PE), Biolegend #372208, Clone: QA16A02, 1:50
• Anti-Granzyme B (APC), Biolegend, #372204, Clone: QA16A02, 1:50
• Anti-Perforin (Alexa Fluor® 647) Biolegend, #308110, Clone: dG9, 1:50
• Anti-FOXP3 (PE), Biolegend, #126404, Clone: MF-14, 1:50
• Anti-CD11b (Brilliant Violet 605), Biolegend, #101257, Clone: M1/70, 1:50
• Anti-CD11c (Alexa Fluor® 488), Biolegend, #117311, Clone:
N418, 1:50
• Anti-CD86 (Brilliant Violet 510), Biolegend, #105040, Clone: GL-1, 1:50
• Anti-I-A/I-E (Alexa Fluor® 647), Biolegend, #107618, Clone: M5/114.15.2, 1:50
• Anti-F4/80 (Brilliant Violet 711), Biolegend, #123147, Clone: BM8, 1:50
• Anti-CD117 (c-kit) (Brilliant Violet 650), Biolegend, #105853, Clone: 2B8, 1:50
• Anti-Ly-6A/E (Sca-1) (Alexa Fluor® 647), Biolegend, #108118, Clone: D7, 1:50
• Anti-Nur77 (Alexa Fluor® 647), BD Bioscience, #566735, Clone: 12.14, 1:50
• Anti-Nur77 (PE), ThermoFisher Scientific, #12-5965-82, Clone: 12.14, 1:50
• Anti-LCK Phospho Tyr394 (PE), Biolegend, #933104, Clone: A18002D, 1:50
• Anti-Phospho-LCK (Tyr505) (PerCP-eFluor™ 710), ThermoFisher Scientific, #46-9076-42, Clone: SRRCHA, 1:50
• Anti-Phospho-ERK1/2 (Thr202, Tyr204) (APC), ThermoFisher Scientific, #17-9109-42, Clone: MILAN8R. 1:50
• Anti-Phospho-ZAP70/Syk (Tyr319, Tyr352) (APC), ThermoFisher Scientific, #17-9006-4, Clone: n3kobu5, 1:50
• Zombie Violet™ Fixable Viability Kit, Biolegend #432113 was used to exclude dead cells 1:1000
• Zombie NIR™ Fixable Viability Kit, Biolegend # 423106 was used to exclude dead cells 1:1000
4-Confocal imaging
• Anti-NFATc2 mouse Abs, Invitrogen, #MA1-025, Clone: 25A10.06.02; 1:500
• Anti-AP1 rabbit Abs, PeproTech, #22114-1 AP; 1:250
• Goat anti-mouse Alexa Fluor™ 488, Invitrogen, #A28175; 1:500
• Goat anti-rabbit Alexa Fluor™ 647, Invitrogen, #A21245; 1:500

Validation

All antibodies were obtained from commercial sources, validated by the vendors, and their validation data are accessible on the manufacturers' websites or have been previously reported in the literature. Western blot detection of SIRT2 and LCK was independently confirmed using genetically deficient mouse and human T cells that do not express these proteins. Prior to use, each antibody was titrated according to the manufacturer's recommendations to determine the optimal dilution and ensure specificity and signal quality in the relevant applications.

Eukaryotic cell lines

Policy information about cell lines and Sex and Gender in Research

Cell line source(s)

• Human Jurkat cells, murine EL4 cells, B16F10 and Yumm1.7 murine melanoma cells, and 293T human embryonic kidney cells were obtained from the American Type Culture Collection (ATCC).

	• KPMSH2KIN lung cancer cells, which express the same epitope recognized in human G12D-mutated KRAS, were obtained from Dr. José Conejo-García (Duke University).<br>• Primary murine T cells were isolated from spleens, lymph nodes, or subcutaneous tumor nodules of mice, as detailed in the Methods section.<br>• Human peripheral blood mononuclear cells (PBMCs) were purified from peripheral blood of healthy donors via density gradient centrifugation using Ficoll-Paque™ PLUS Media (GE Healthcare).<br>• Primary human tumor cells and matched tumor-infiltrating lymphocytes (TILs) were isolated from tumor biopsies of patients with non–small cell lung cancer (NSCLC) or small cell lung cancer (SCLC).
Authentication	No formal cell line authentication was performed, as the majority of cells used in this study were primary cells.<br>Human Jurkat cells, murine EL4 cells, B16F10 and Yumm1.7 murine melanoma cells, and 293T human embryonic kidney cells were obtained directly from the American Type Culture Collection (ATCC) and used at low passage numbers. All ATCC cell lines were maintained under standard culture conditions and were regularly monitored for morphology and proliferation rates. No abnormal behavior or morphology was observed in culture.
Mycoplasma contamination	All cell lines used in this study were routinely tested for mycoplasma contamination and were confirmed to be negative.
Commonly misidentified lines<br>(See ICLAC register)	No commonly misidentified lines were used.

Animals and other research organisms

Policy information about studies involving animals; ARRIVE guidelines recommended for reporting animal research, and Sex and Gender in Research

Laboratory animals	• C57BL/6J Mice, Jackson Laboratories.<br>• C57BL/6SJL mice, Jackson Laboratories.<br>• PMEL mice with gp100-reactive TCR: B6.Cg-Thy1a/Cy Tg(TcraTcrb)8Rest/J, Jackson Laboratories.<br>• OT-II mice with MHC II-restricted OVA-specific TCR: B6.Cg-Tg(TcraTcrb)425Cbn/J, Jackson Laboratories.<br>• Sirt2-/- mice: B6.129-Sirt2tm1.1Fwa/J, Jackson Laboratories.<br>• B6.Cg-Rag2tm1.1Cgn/J (Rag2-/-), Jackson Laboratories.<br>• NSG mice: NOD.Cg-Prkdcscid Il2rgtm1Wjl/SzJ, Jackson Laboratory.<br>• PMEL mice and OT-II mice were crossed with Sirt2-/- mice to generate Sirt2-/- PMEL mice and Sirt2-/- OT-II mice, respectively.<br>• C57BL/6J (CD45.2) and C57BL/6.SJL-Ptprca (CD45.1) mice were crossed to generate F1 hybrid CD45.1 × CD45.2 mice.<br>All mice were bred and maintained under specific pathogen–free (SPF) conditions at the animal facility of Mayo Clinic, Jacksonville, FL. Mice were housed under controlled environmental conditions with a 12-hour light/dark cycle, ambient temperature of 20–24 °C, and relative humidity of 40–60%, with ad libitum access to autoclaved food and water. All animal protocols were approved by the IACUC of Mayo Clinic and conducted in accordance with institutional and NIH guidelines. Mice were used at 7–8 weeks of age, with age- and sex-matched controls included in all experiments.
Wild animals	No wild animals were involved in this study.
Reporting on sex	Both, male and female mice were used.
Field-collected samples	No field collected samples were involved in this study.
Ethics oversight	All animal protocols were approved by the Institutional Animal Care and Use Committee:<br>-IACUC protocol #: A00007348<br>-IACUC protocol #: A00007297

Note that full information on the approval of the study protocol must also be provided in the manuscript.

Plants

Seed stocks	N/A
Novel plant genotypes	N/A
Authentication	N/A

Flow Cytometry

Plots

Confirm that:

☒ The axis labels state the marker and fluorochrome used (e.g. CD4-FITC).

☒ The axis scales are clearly visible. Include numbers along axes only for bottom left plot of group (a 'group' is an analysis of identical markers).

☒ All plots are contour plots with outliers or pseudocolor plots.

☒ A numerical value for number of cells or percentage (with statistics) is provided.

Methodology

Sample preparation	• Mouse spleens and lymph nodes were harvested, mechanically dissociated between frosted glass slides, and filtered through 70µm cell strainers. Red blood cells were lysed using RBC Lysis Buffer (BioLegend). $CD3^+$, $CD4^+$, and $CD8^+$ T cells were negatively enriched using Pan T Cell, $CD4^+$ T Cell, and $CD8^+$ T Cell Isolation Kits (Miltenyi Biotec).<br>• Human PBMCs were isolated from healthy donor peripheral blood by density gradient centrifugation using Ficoll-Paque™ PLUS (GE Healthcare). $CD3^+$ T cells were negatively enriched using a human Pan T Cell Isolation Kit (Miltenyi Biotec).<br>• Tumor-infiltrating lymphocytes (TILs) were isolated from subcutaneous tumor nodules by enzymatic digestion with collagenase type IV (2mg/mL, Worthington Biochemical) and DNase I (0.25mg/mL, Sigma-Aldrich) for 45 minutes at 37°C with agitation. Digested tissues were filtered sequentially through 100µm and 40µm strainers (Thermo Fisher Scientific), washed with PBS, and treated with RBC Lysis Buffer (BioLegend). TILs were enriched by Percoll density gradient centrifugation (GE Healthcare) and purified using the CD3ε MicroBeads Kit™ (Miltenyi Biotec).<br>• For surface marker analysis, mouse T cells were stained in PBS containing 2% BSA (FACS buffer) with combinations of directly labelled antibodies for extracellular markers. Cells were incubated at 4°C for 20 minutes to 1 hour, washed twice with FACS buffer, and fixed in PBS containing 1% paraformaldehyde. Dead cells were excluded using the Zombie Violet or Zombie NIR Fixable<br>• For cytokine intracellular staining, cells were first re-stimulated with phorbol 12-myristate 13-acetate (PMA, 10ng/ml, Sigma-Aldrich) and ionomycin (1µM, Sigma-Aldrich) for 1 hour, followed by GolgiPlug™ treatment (1‰, BD Biosciences) for an additional 6 hours. Cells were then surface stained prior to fixation/permeabilization using the Cytofix/Cytoperm™ Kit (BD Biosciences), followed by intracellular staining with combinations of directly labelled antibodies for cytokines.<br>• For FoxP3 intracellular staining, cells were first surface stained prior to fixation/permeabilization using the eBioscience™ Foxp3/Transcription Factor Staining Buffer Set (Thermo Fisher Scientific) according to the manufacturer's instructions, followed by intracellular staining with FoxP3 (PE, BioLegend).<br>• For Nur77 intracellular staining, cells were first surface stained prior to fixation/permeabilization using the BD Pharmingen™ Transcription Factor Buffer Set (BD Biosciences) according to the manufacturer's instructions, followed by intracellular staining with Nur77 (PE or AF647, BioLegend).<br>• For proximal TCR signaling analysis, cells were surface stained before fixation/permeabilization with Cytofix/Cytoperm™ Kit (BD Biosciences), followed by intracellular staining with combinations of directly labelled antibodies for proximal TCR targets.
Instrument	Data were acquired using BD FACSymphony™ A5, BD LSR II (Becton Dickinson), and Cytek® Aurora (Cytek Biosciences) instruments. BD FASC Diva™ software version 8 and Cytek SpectroFlo v3.2.1 were used to collect data from flow cytometry.
Software	• Flow cytometry data were acquired using BD FACSDiva™ Software v8.0 (BD Biosciences) and SpectroFlo® Software v3.2.1 (Cytek Biosciences).<br>• Data were further analyzed using FlowJo™ v10 (BD Biosciences).
Cell population abundance	An aliquot of sorted cell populations was routinely collected and analyzed by flow cytometry to verify sample purity, which consistently exceeded 95%.<br>Cell frequencies and population abundances are indicated in flow cytometry plots (insets) and are quantitatively represented in accompanying histograms.
Gating strategy	Flow cytometry analysis was performed using the following sequential gating strategies:<br>• T cells: Lymphocytes were first gated based on FSC-H vs. SSC-H, followed by singlet selection using FSC-H vs. FSC-A and SSC-H vs. SSC-A. Live cells were identified as Zombie-. B cells were excluded (B220-), and T cells were gated as CD3+. CD4+ and CD8+ subsets were then identified.<br>• Regulatory T cells (Tregs): Gating followed the same initial steps (lymphocyte, singlet, live). Cells were gated on CD3+, followed by CD4+CD8- cells. Tregs were defined as CD44+Foxp3+ within the CD4+ population.<br>• NK cells: Following lymphocyte, singlet, and live cell gating, lineage-negative cells were selected (B220-, CD19-, CD11b-, Gr1-). T cells were excluded (CD3-), and NK cells were identified as NK1.1+.<br>• Dendritic cells (DCs): Myeloid cells were gated based on FSC-H vs. SSC-H, followed by singlet and live cell gating. Non-DCs were excluded (B220-, CD19-, TER119-, Gr1-, NK1.1-, CD3-), and DCs were defined as CD11b+CD11c+.<br>• Hematopoietic stem cells (HSCs) in bone marrow: Cells were gated on FSC-H vs. SSC-H, singlets were selected, and live cells identified as Zombie-. Lineage-negative cells (B220-, CD19-, TER119-, CD11b-, Gr1-, NK1.1-) were gated, with mature T cells excluded (CD3-). HSCs were defined as c-Kit+Sca-1+.<br>• T cell development: In thymus, spleen, and lymph nodes of bone marrow chimeric mice, CD45.1+ (WT) and CD45.2+ (Sirt2-/-) T cells were assessed for CD4 and CD8 expression. DN1–DN4 stages were gated based on CD44 and CD25 expression, and DN1 cells were further analyzed for c-Kit+Sca-1+ phenotype.<br>Gating thresholds for positive and negative populations were set using isotype or biological control samples. Cytokine-

producing populations were identified by comparison to isotype-stained controls.

☒ Tick this box to confirm that a figure exemplifying the gating strategy is provided in the Supplementary Information.
