# Supplementary material for: SIRT2-mediated deacetylation of LCK governs the magnitude of T cell receptor signaling
Source: Nat Immunol. 2026 Jan 29;27(2):213–24. doi: 10.1038/s41590-025-02377-3 (PMC12864039; doi:10.1038/s41590-025-02377-3)

Fig. 1h

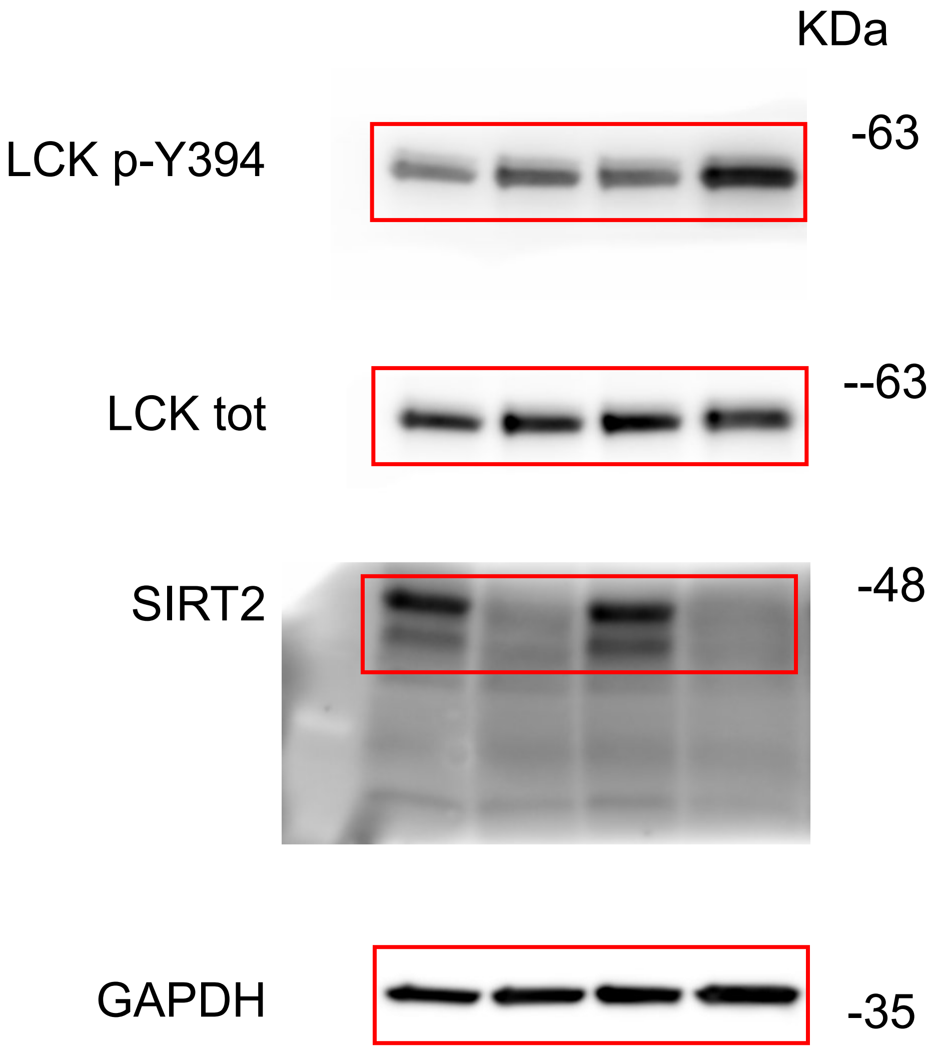

Fig. 1i

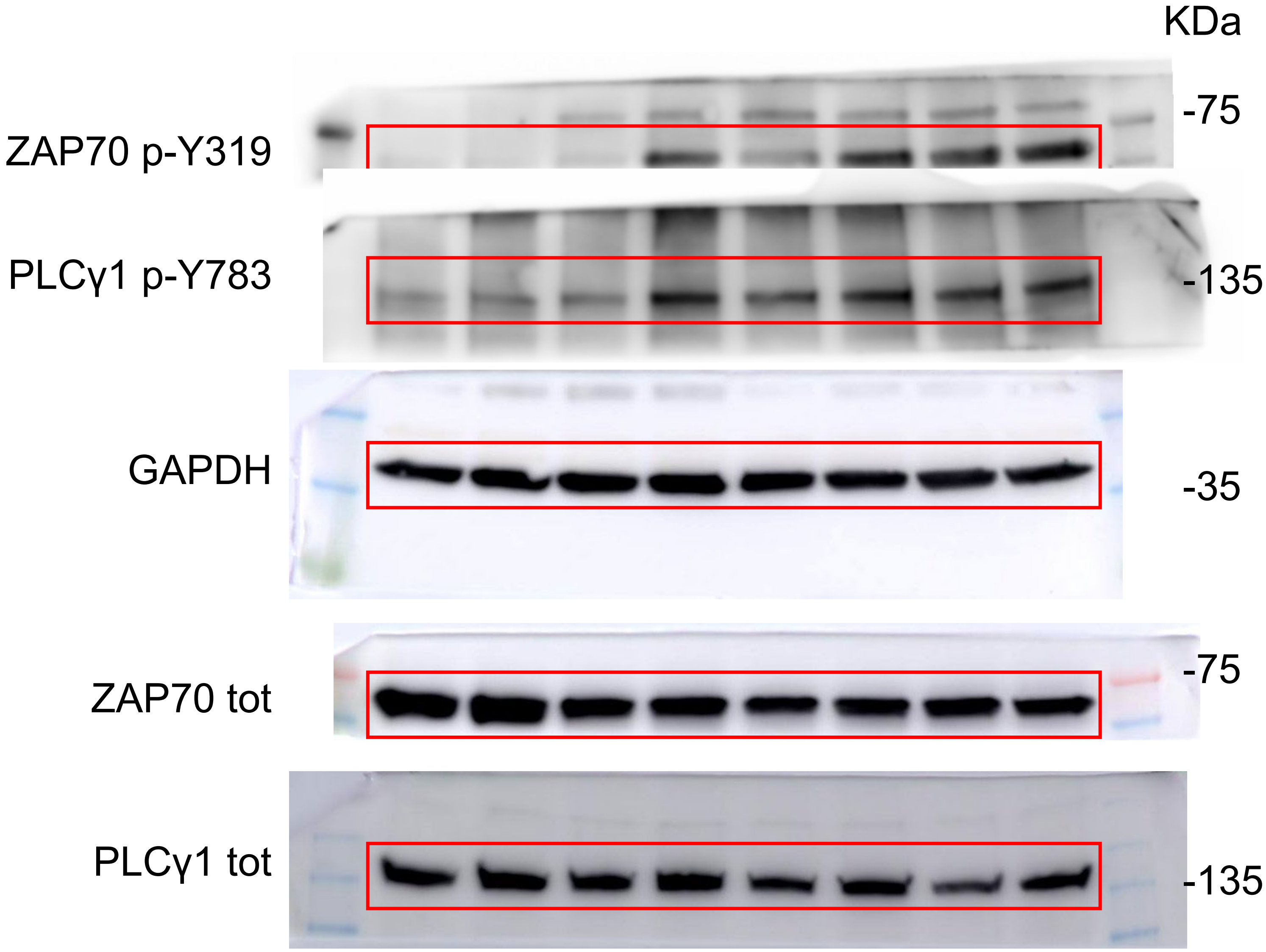
KDa
ZAP70 p-Y319
-75
PLCγ1 p-Y783
-135
GAPDH
-35
ZAP70 tot
-75
PLCγ1 tot
-135

Fig. 1j

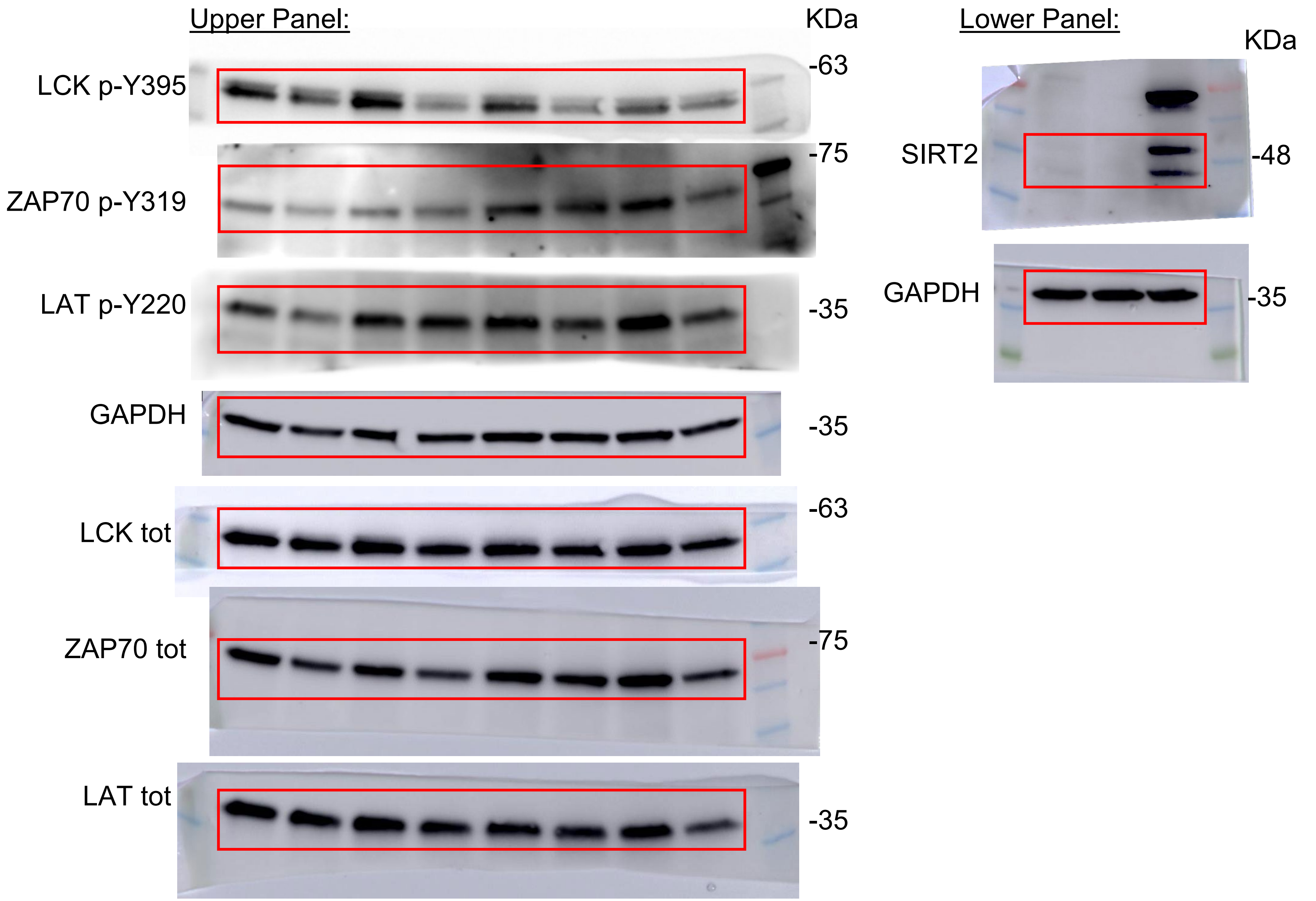
Upper Panel:
KDa
LCK p-Y395
-63
-75
ZAP70 p-Y319
LAT p-Y220
-35
GAPDH
-35
-63
LCK tot
-75
ZAP70 tot
LAT tot
-35
Lower Panel:
KDa
SIRT2
-48
GAPDH
-35

Fig. 2d

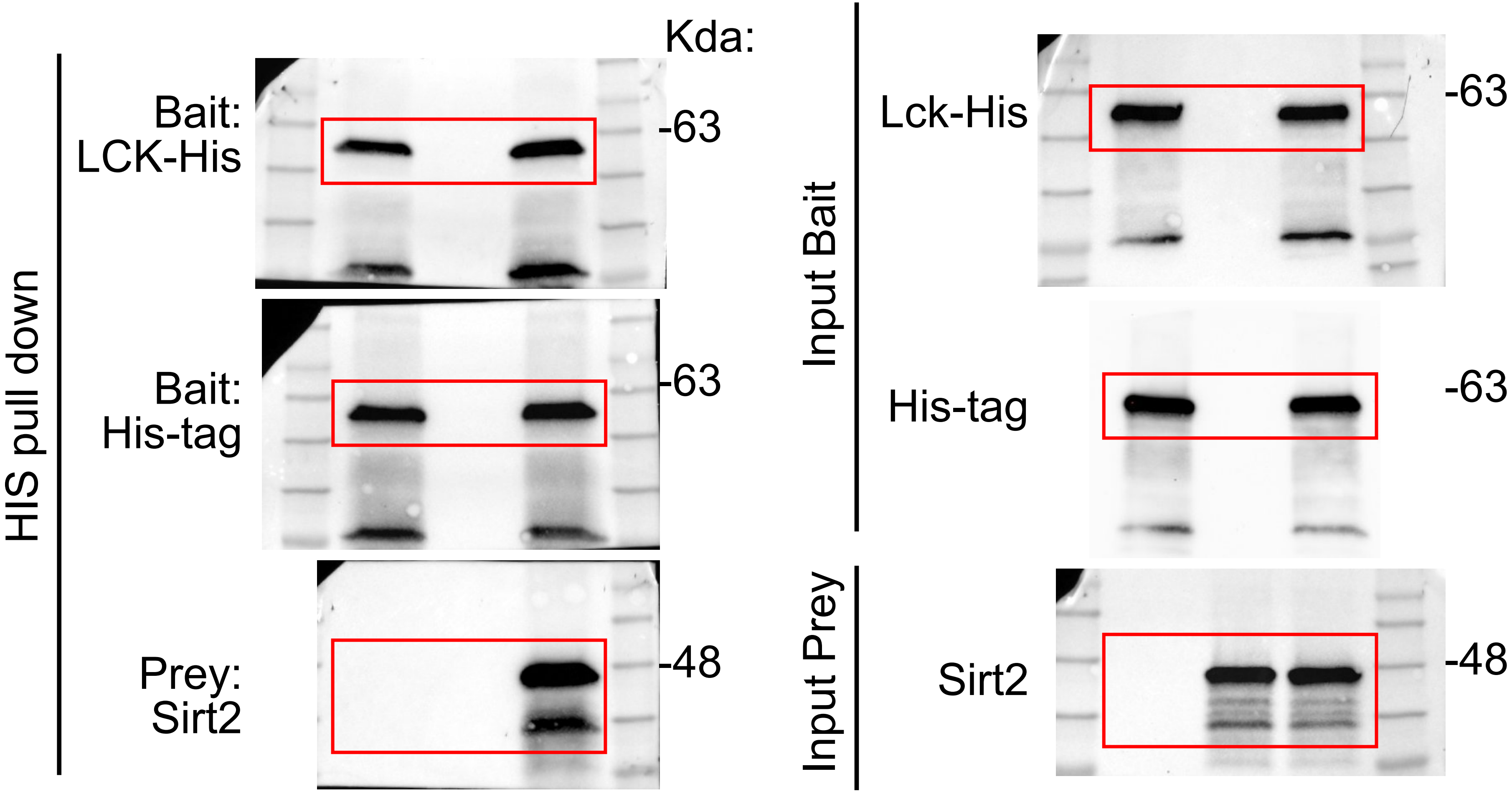
Kda:
HIS pull down
Bait:
LCK-His
-63
Bait:
His-tag
-63
Prey:
Sirt2
-48
Input Bait
Lck-His
-63
His-tag
-63
Input Prey
Sirt2
-48

Fig. 2I

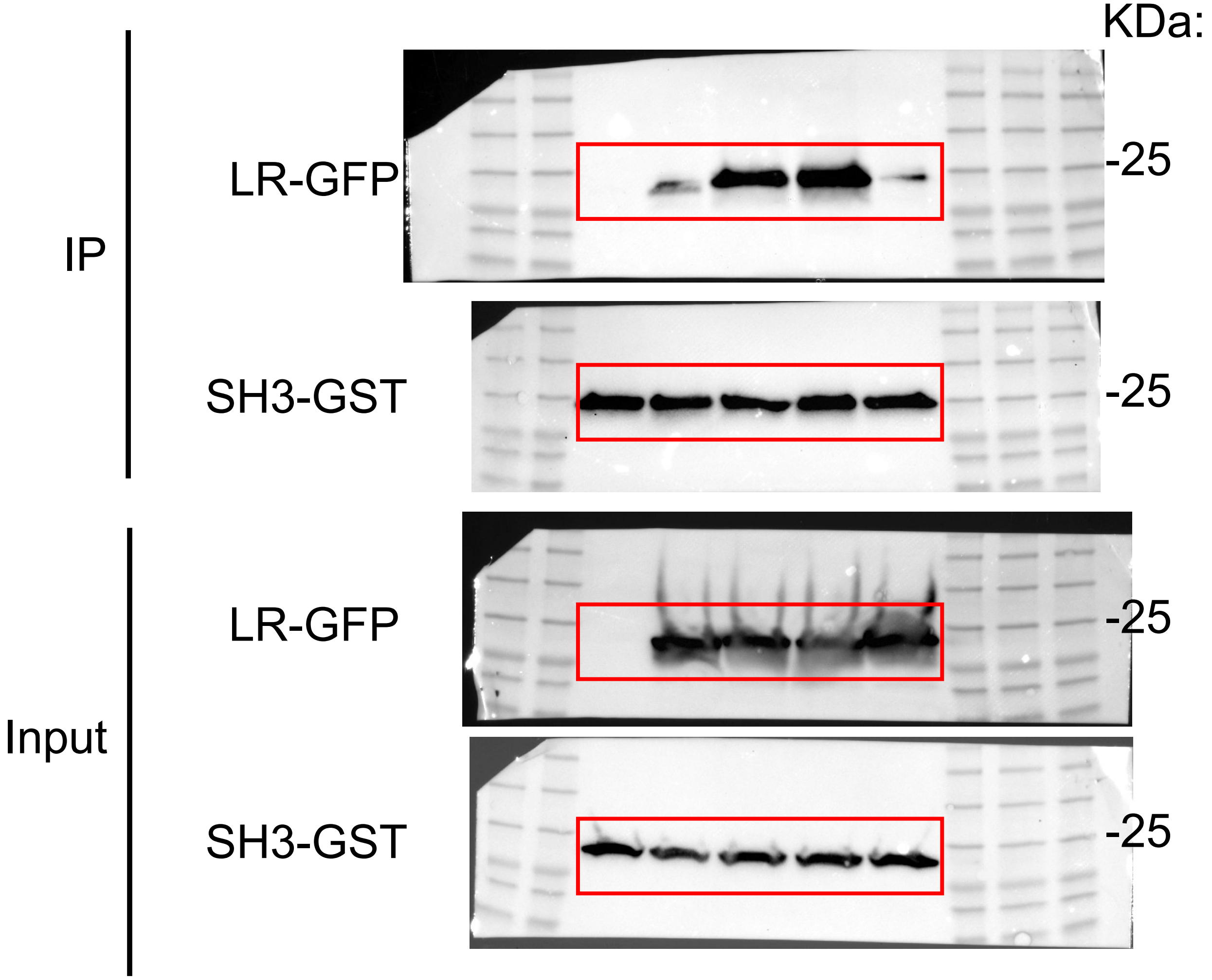

Fig. 2o

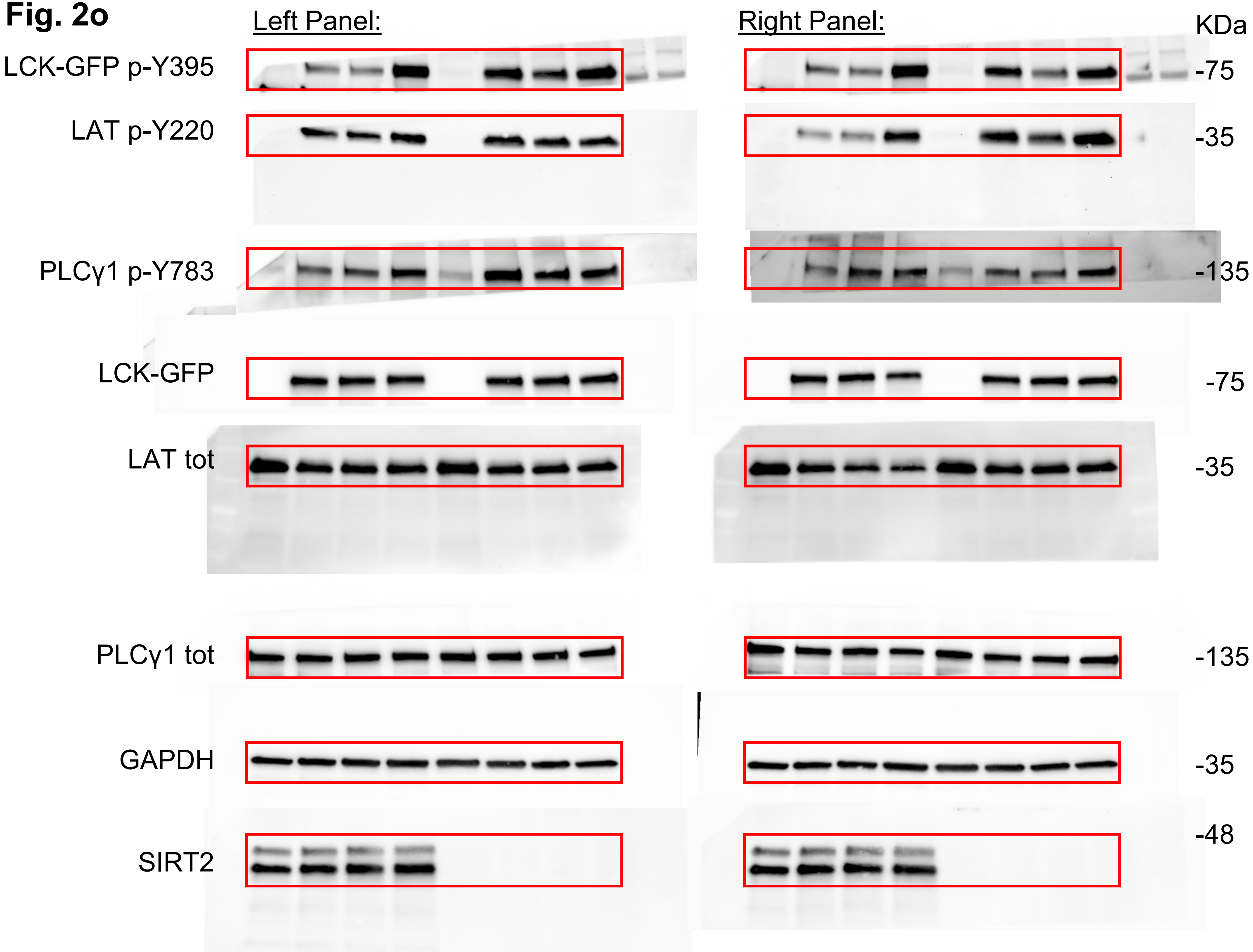
Left Panel:
Right Panel:
KDa
LCK-GFP p-Y395
-75
LAT p-Y220
-35
PLCγ1 p-Y783
-135
LCK-GFP
-75
LAT tot
-35
PLCγ1 tot
-135
GAPDH
-35
SIRT2
-48

Fig. 3a

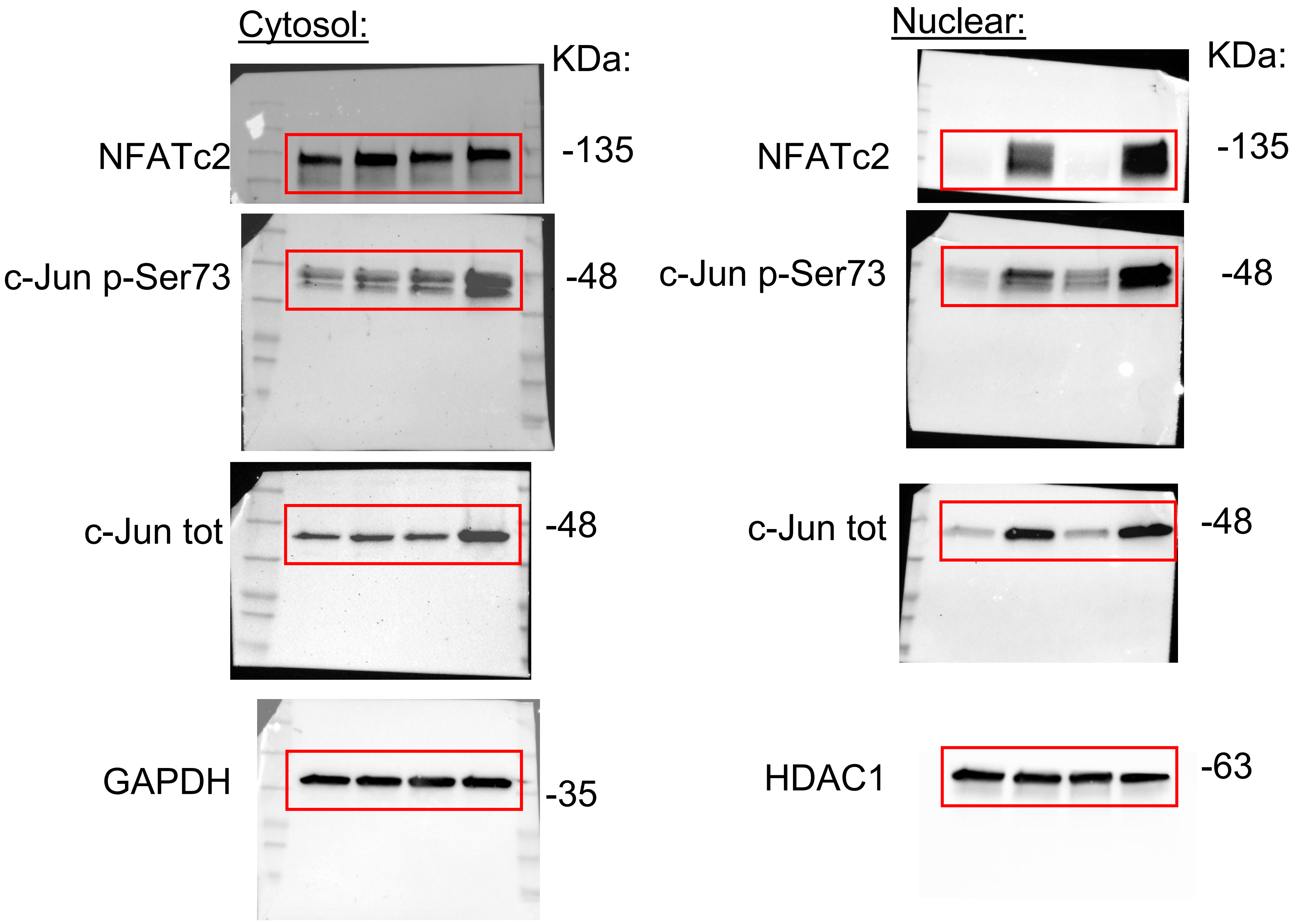

Cytosol:
KDa:
NFATc2
-135
c-Jun p-Ser73
-48
c-Jun tot
-48
GAPDH
-35
Nuclear:
KDa:
NFATc2
-135
c-Jun p-Ser73
-48
c-Jun tot
-48
HDAC1
-63

Fig. 3b

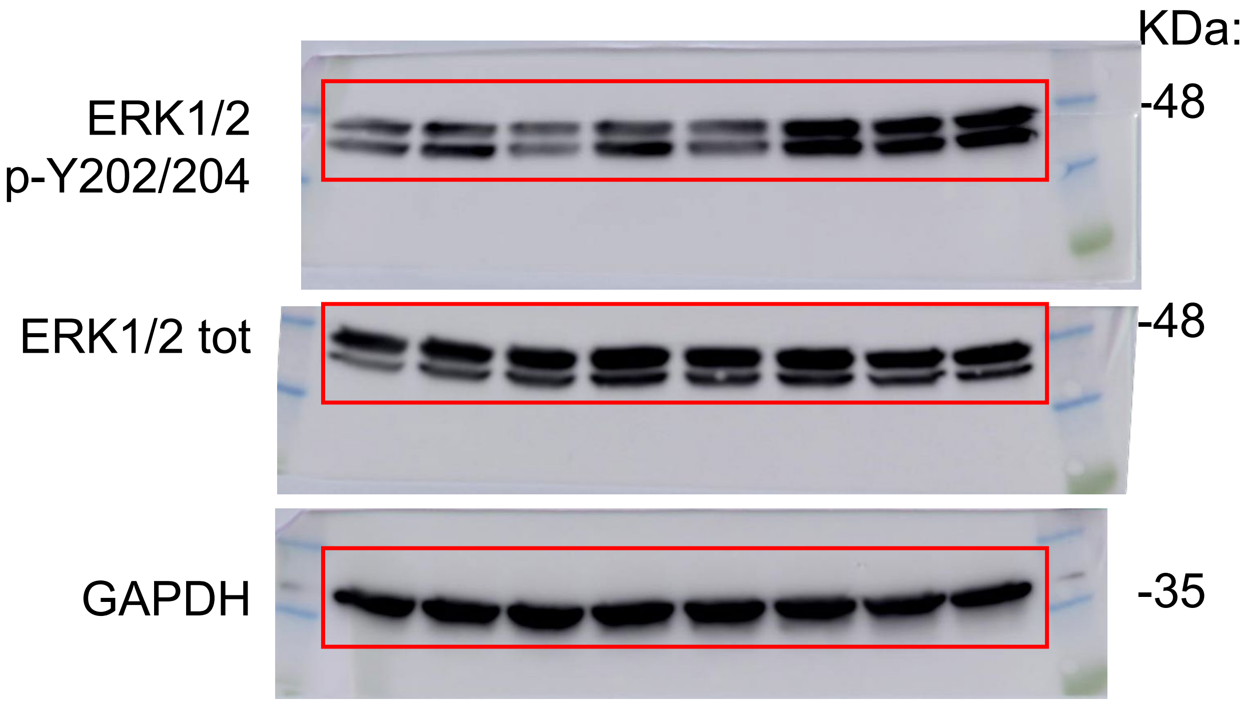

Fig. 5a

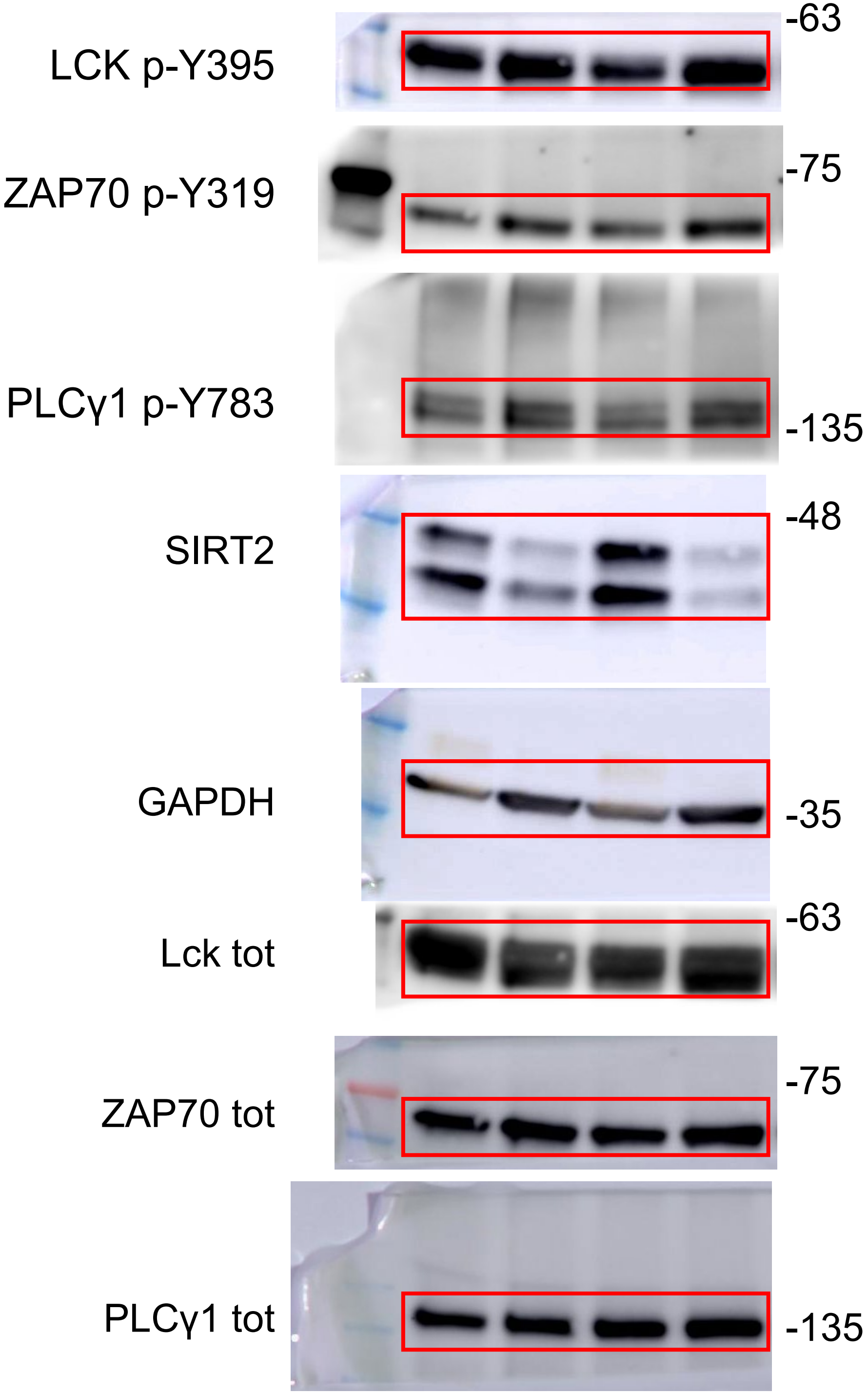

LCK p-Y395
-63
ZAP70 p-Y319
-75
PLCγ1 p-Y783
-135
SIRT2
-48
GAPDH
-35
Lck tot
-63
ZAP70 tot
-75
PLCγ1 tot
-135

Fig. 5b

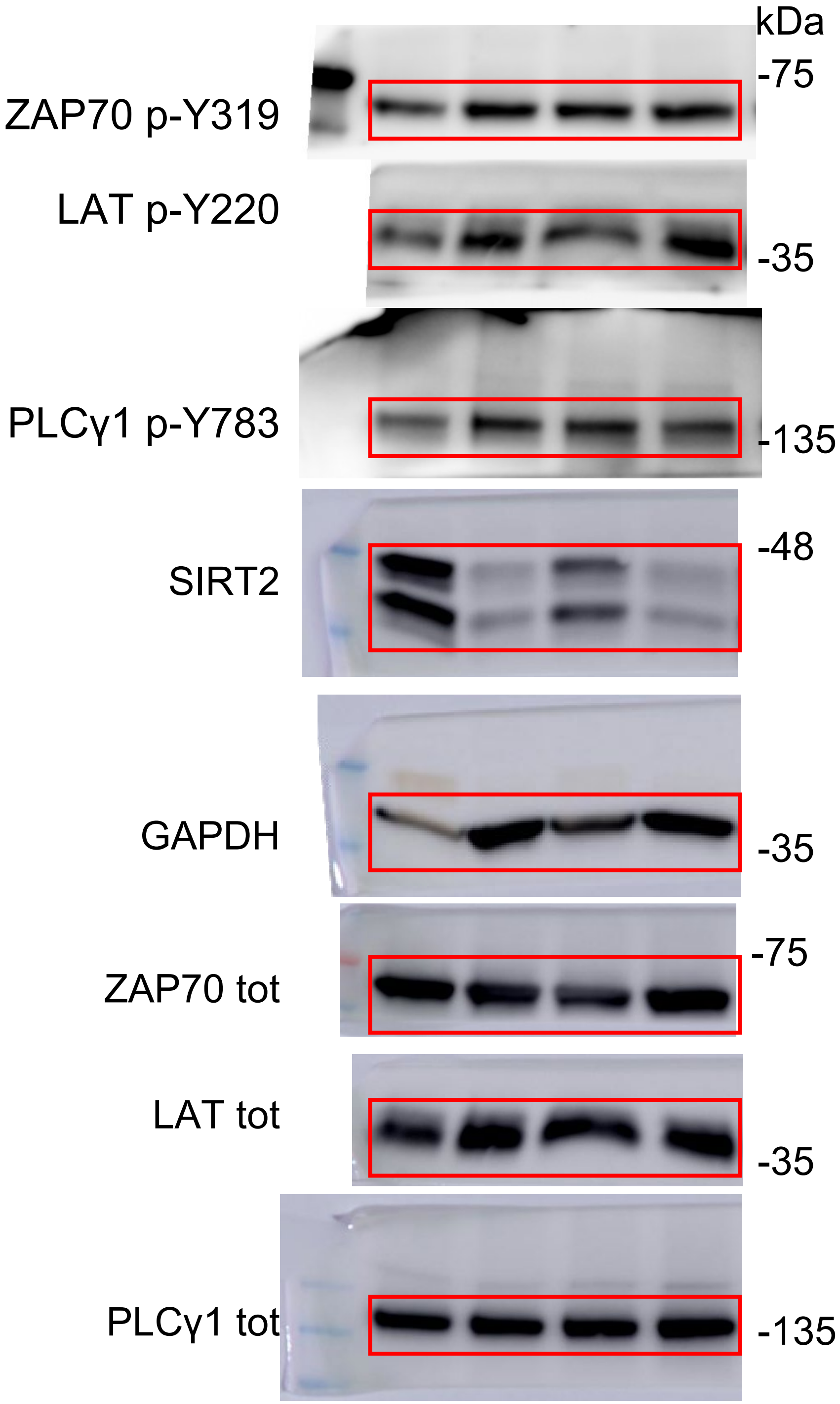

kDa
ZAP70 p-Y319
-75
LAT p-Y220
-35
PLCγ1 p-Y783
-135
SIRT2
-48
GAPDH
-35
ZAP70 tot
-75
LAT tot
-35
PLCγ1 tot
-135

Fig. 5g

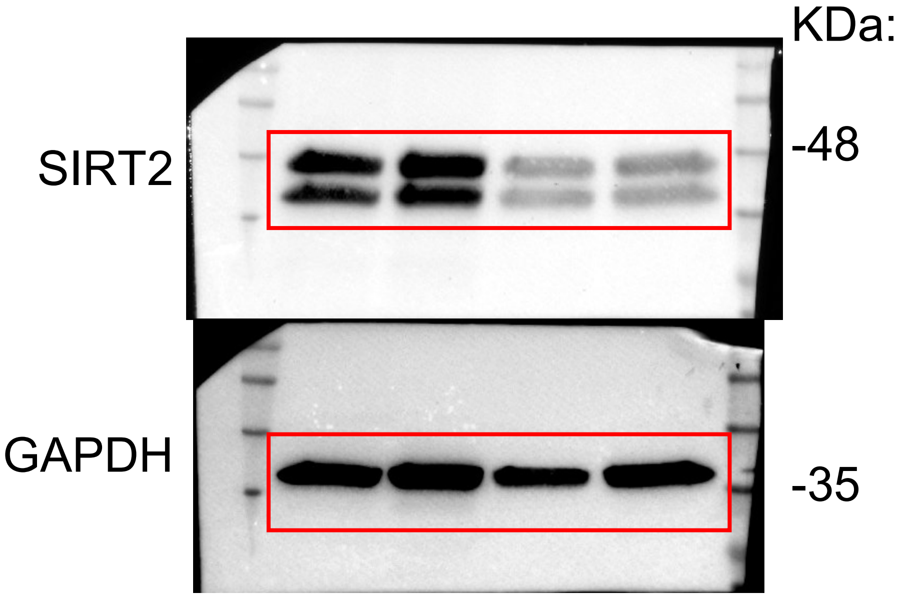

Fig. 5i

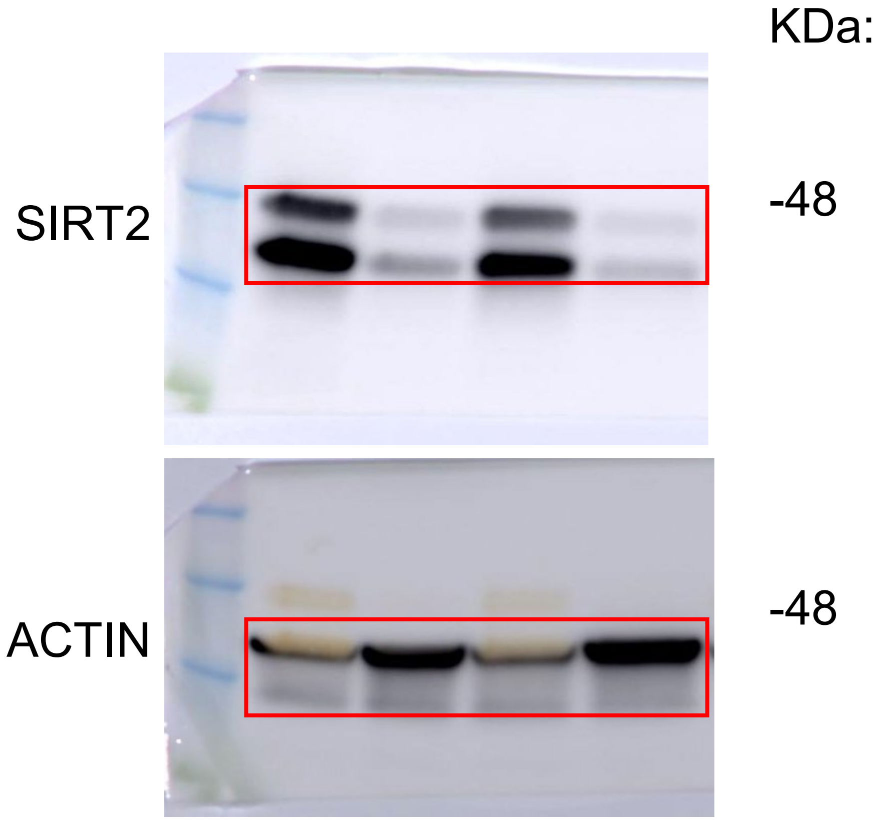

Extended Data Fig. 1c

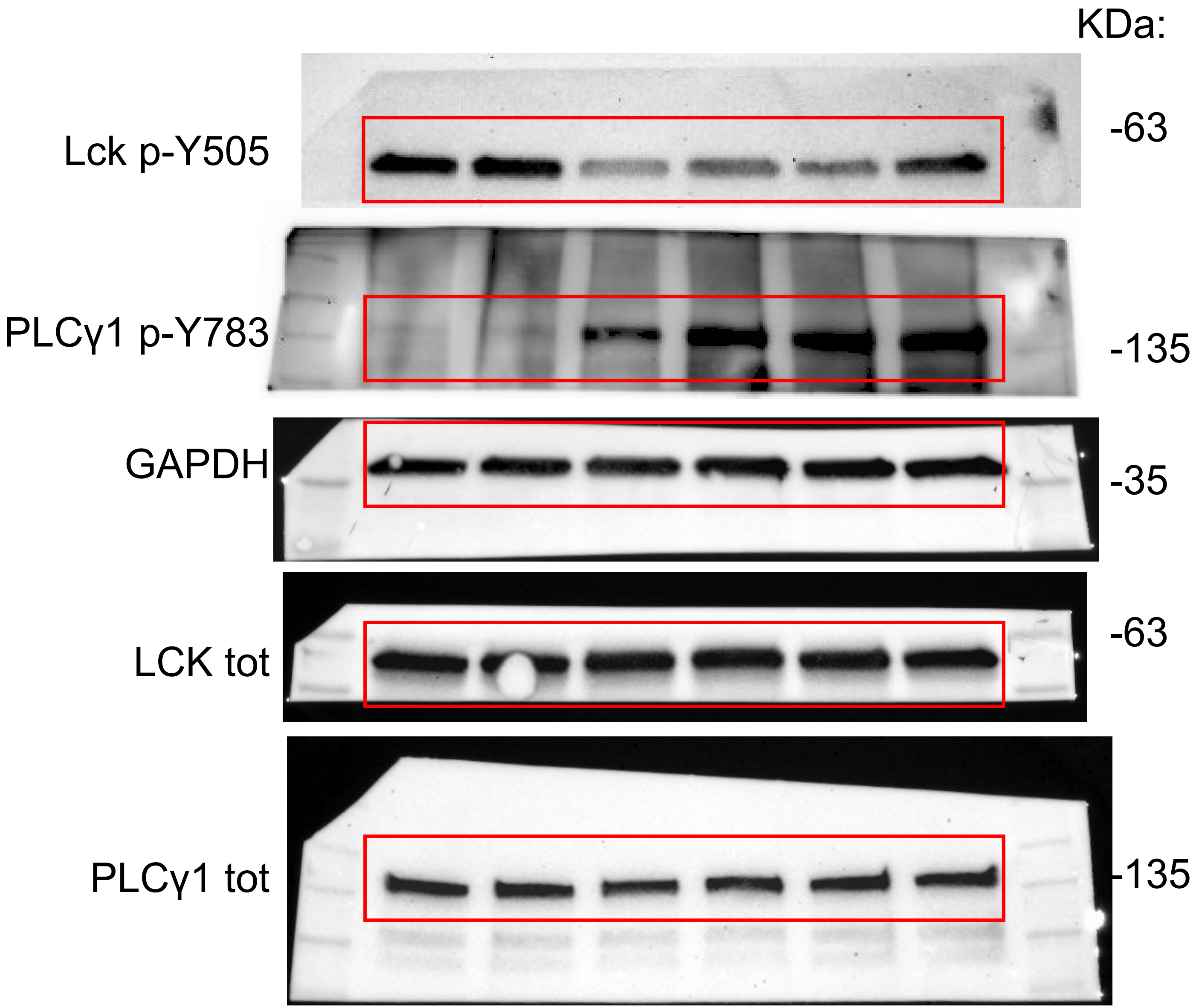

Extended Data Fig. 1d

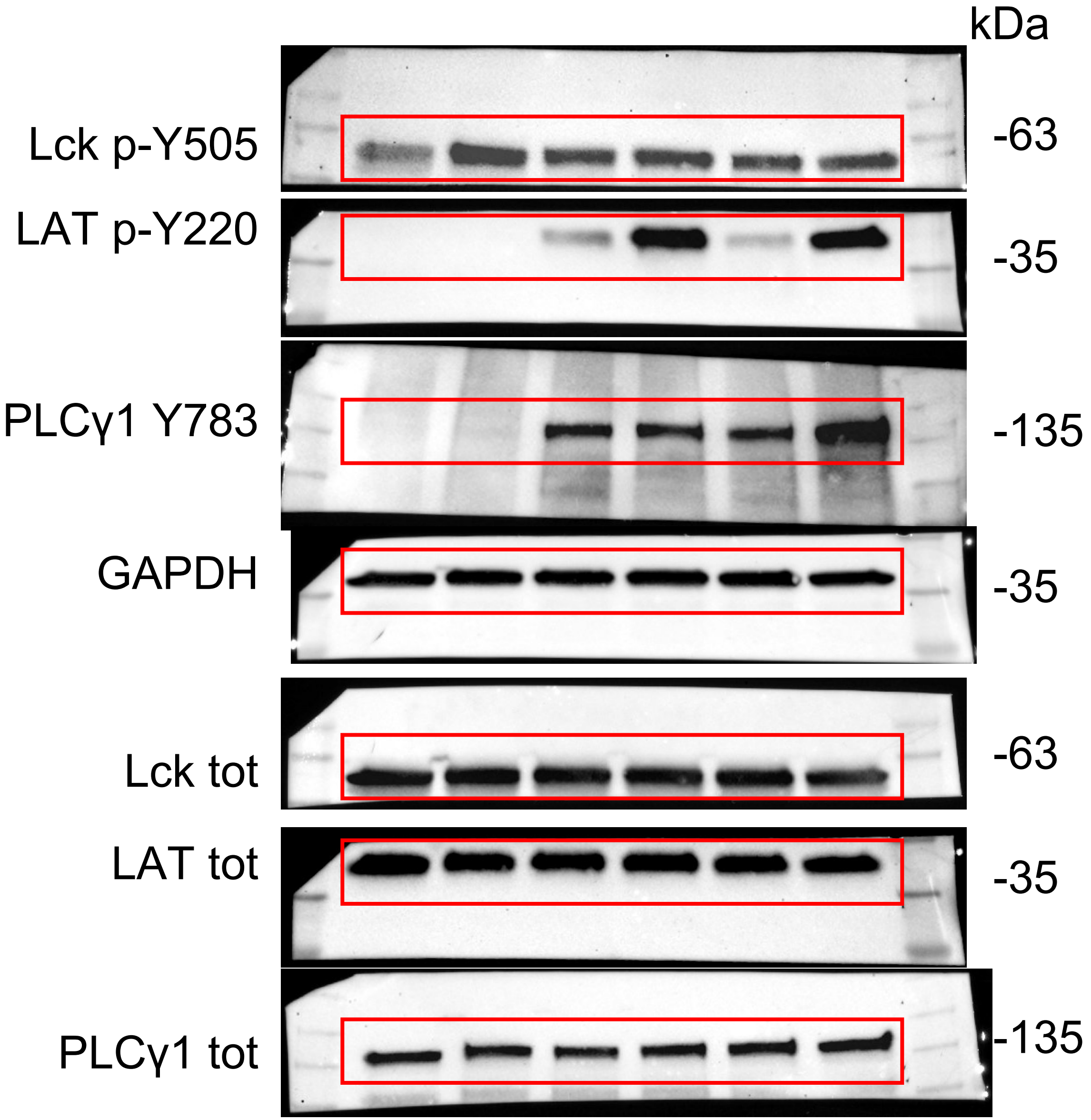

Extended Data Fig. 1e

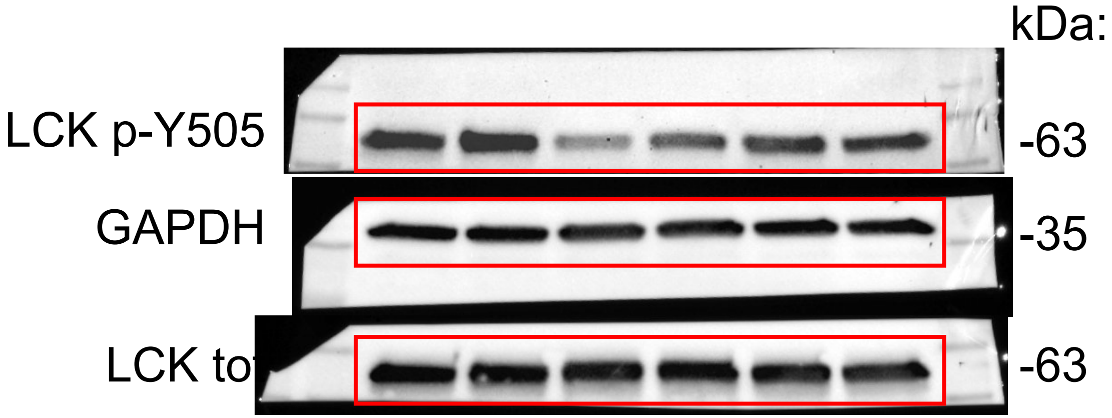

Extended Data Fig. 1f

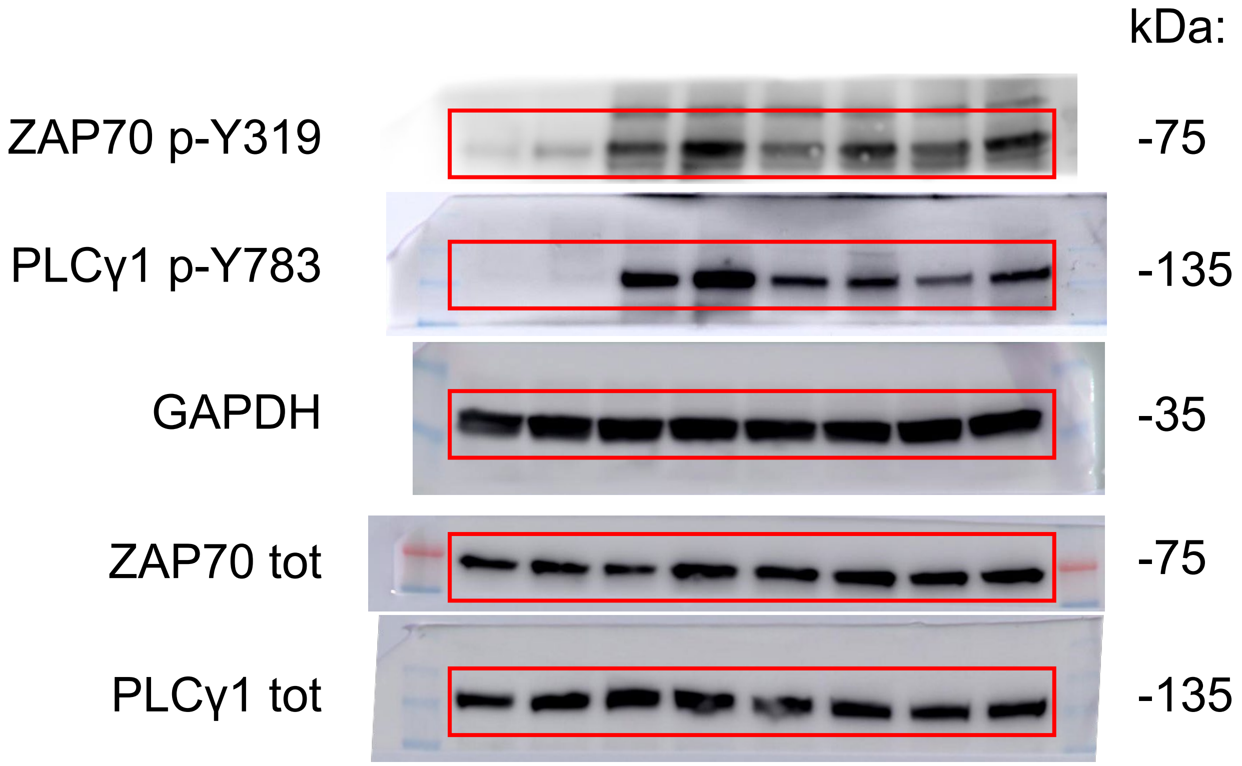

Extended Data Fig. 1g

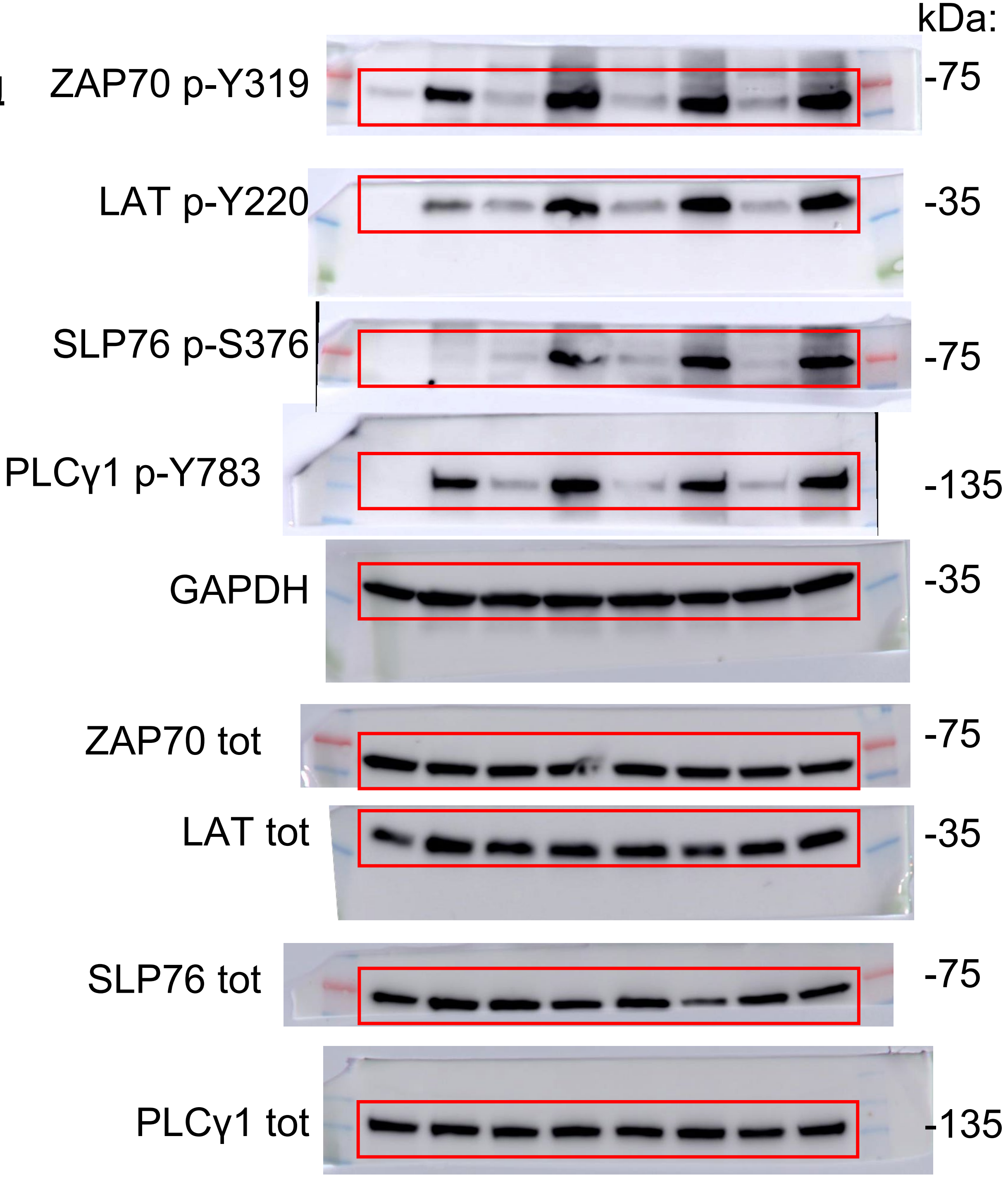

Extended Data Fig. 1i

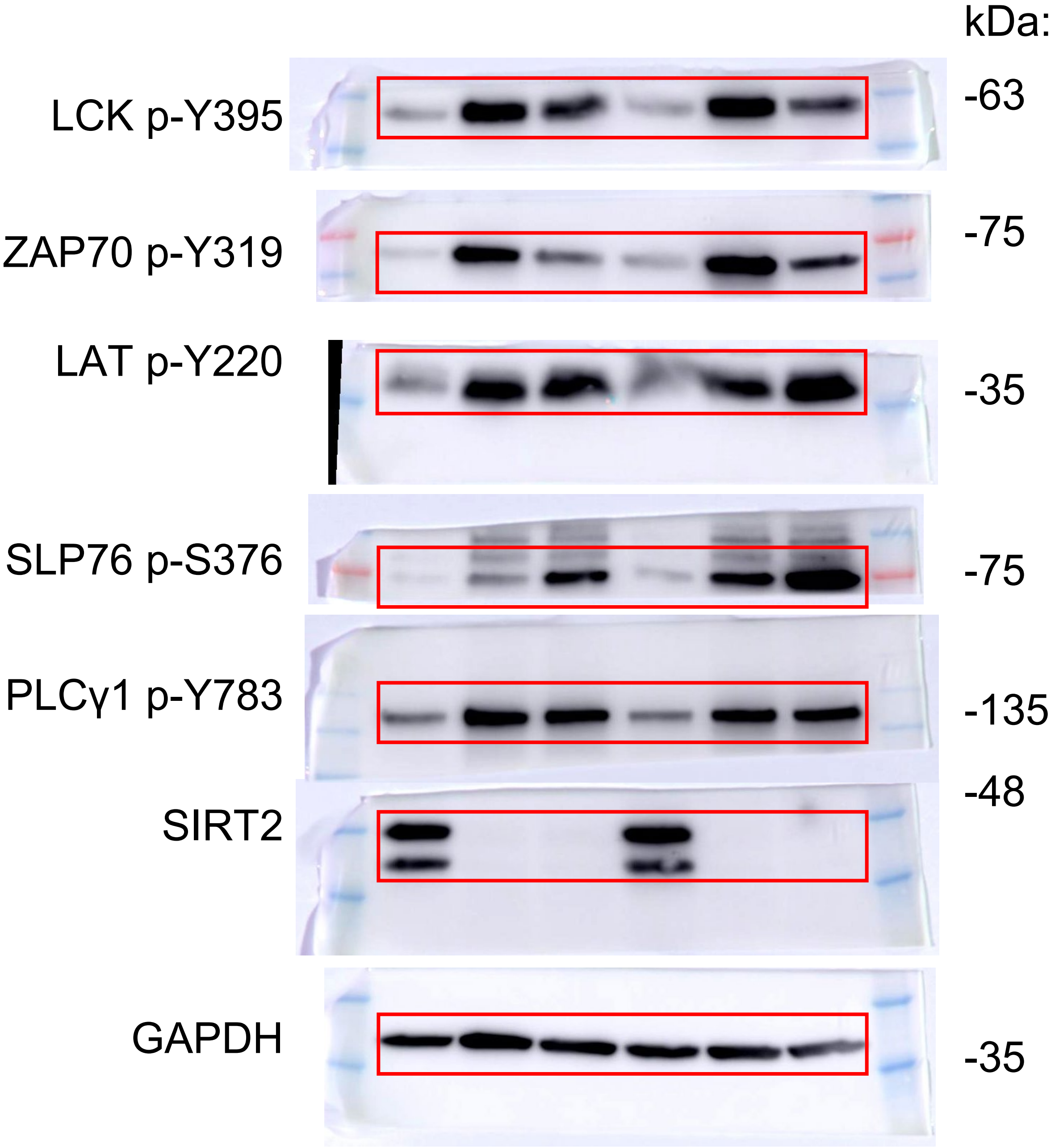

Extended Data Fig. 1j

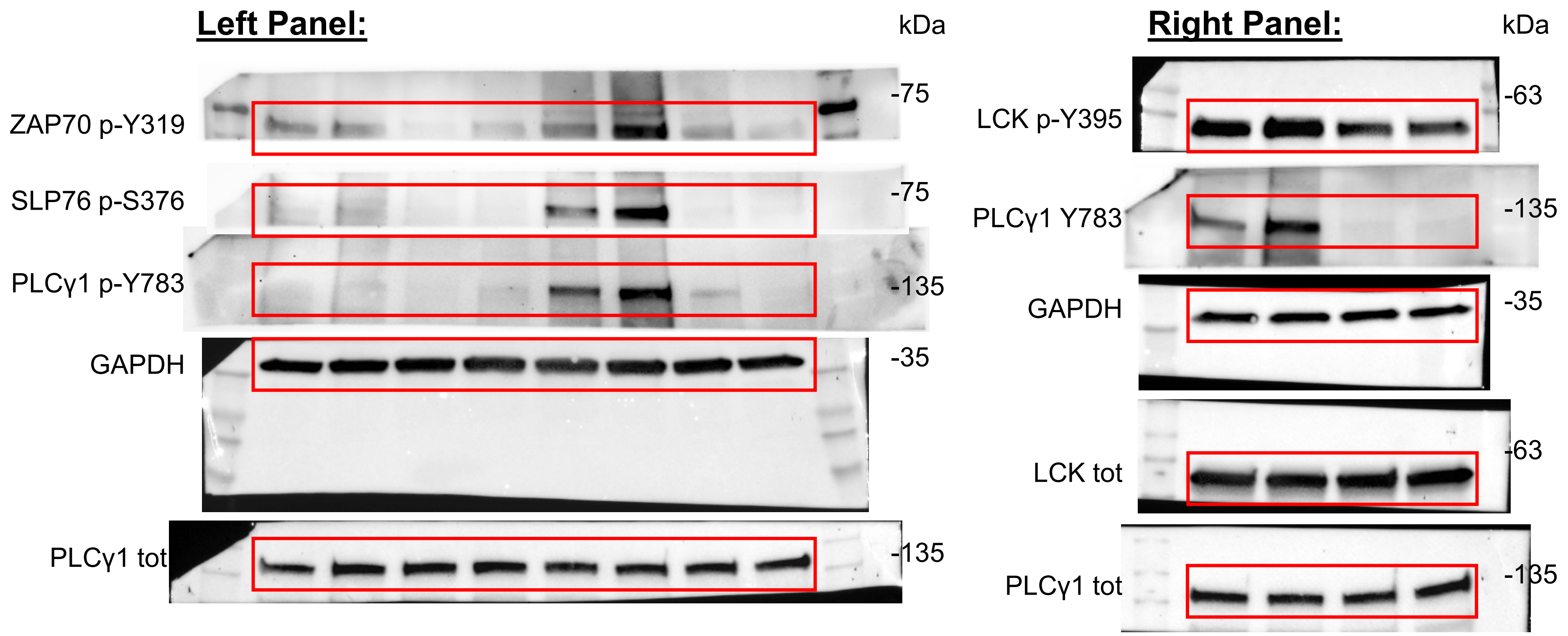

Extended Data Fig. 1k

Left Panel:

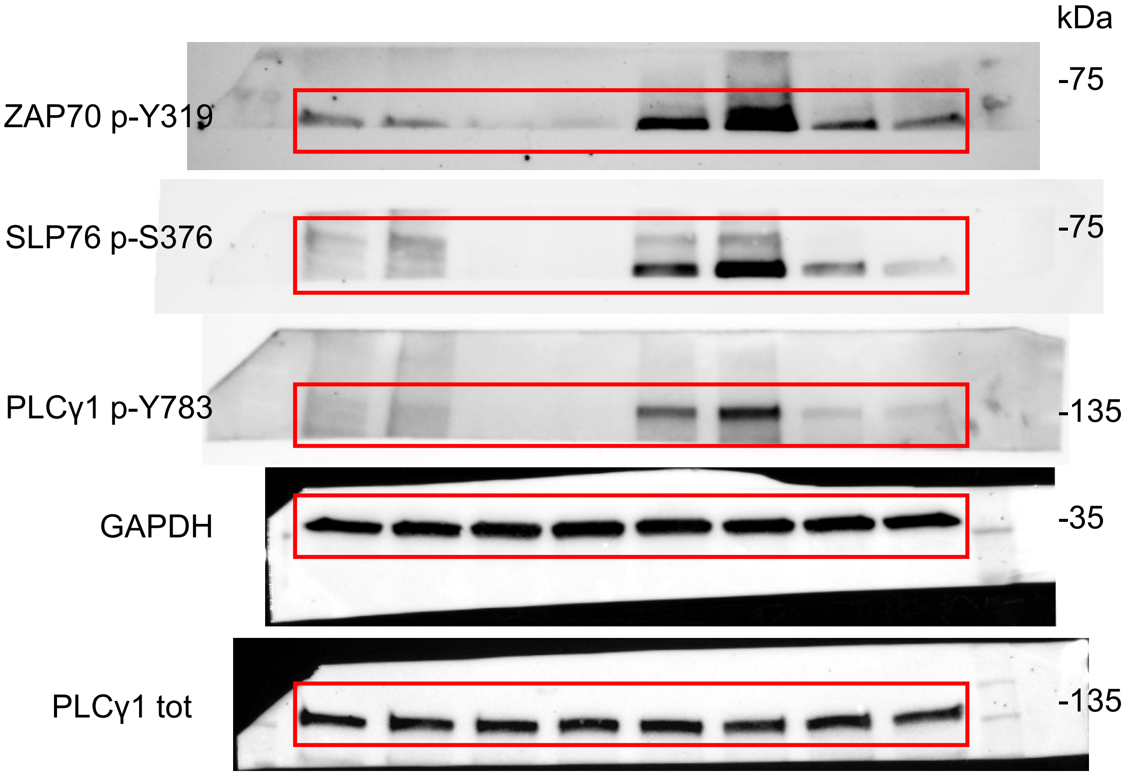

Right Panel:

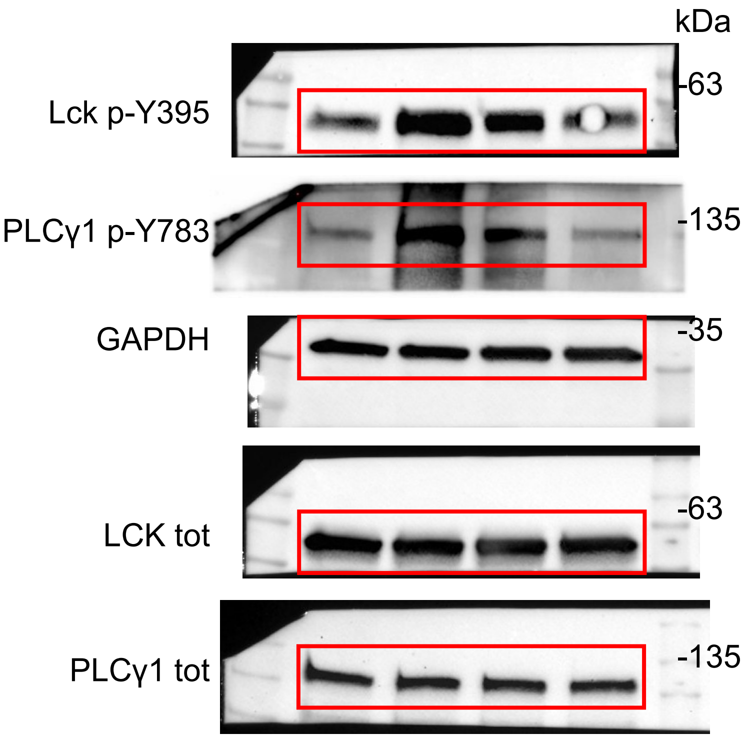

Extended Data Fig. 3d

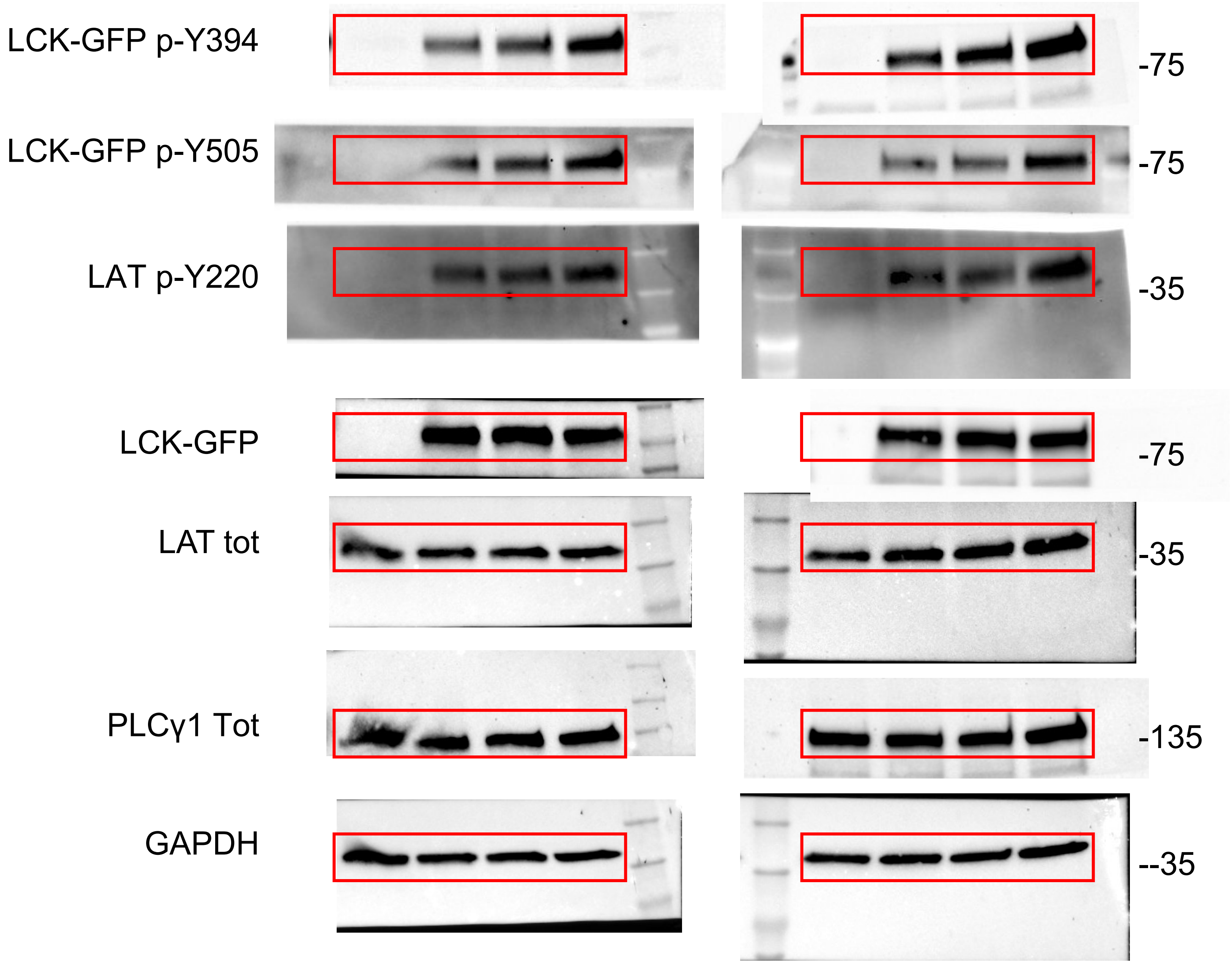

Extended Data Fig. 3e

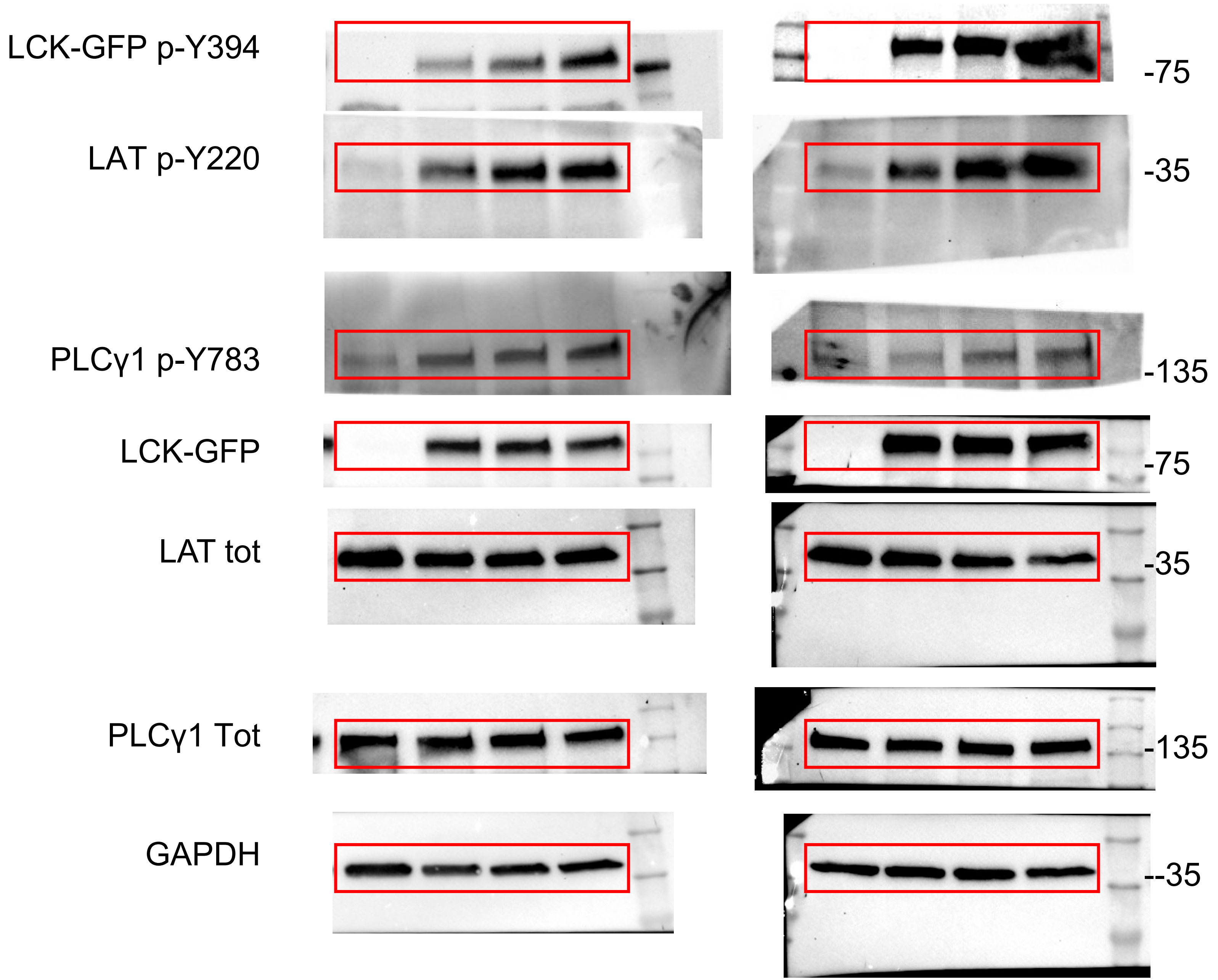

Extended Data Fig. 3h

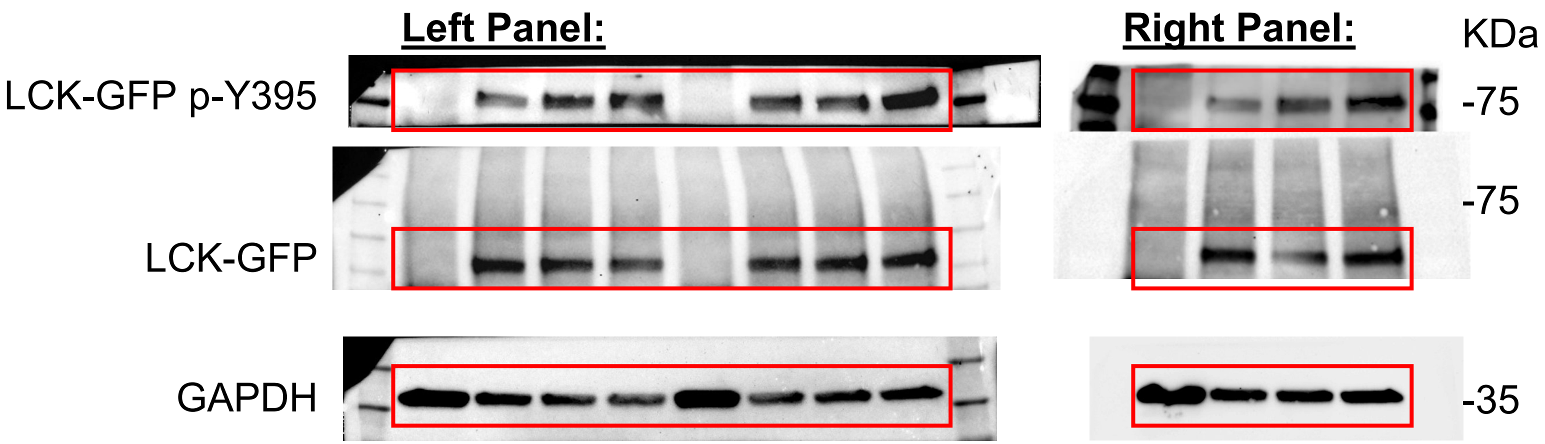

Extended Data Fig. 3j

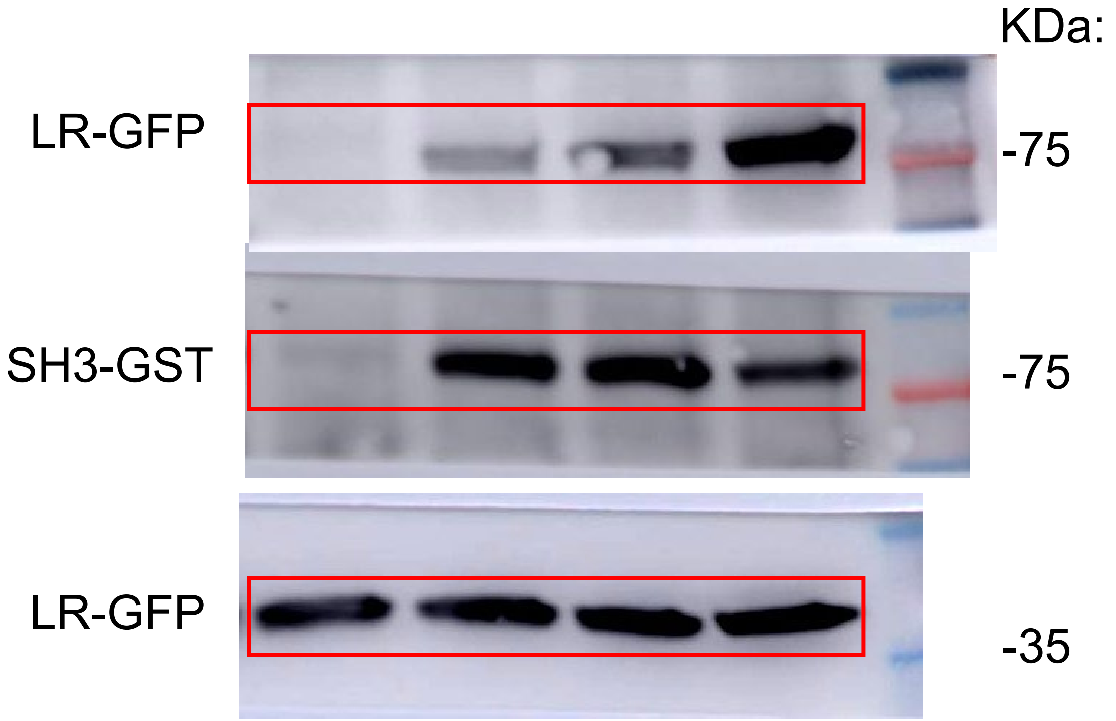

Extended Data Fig. 4a

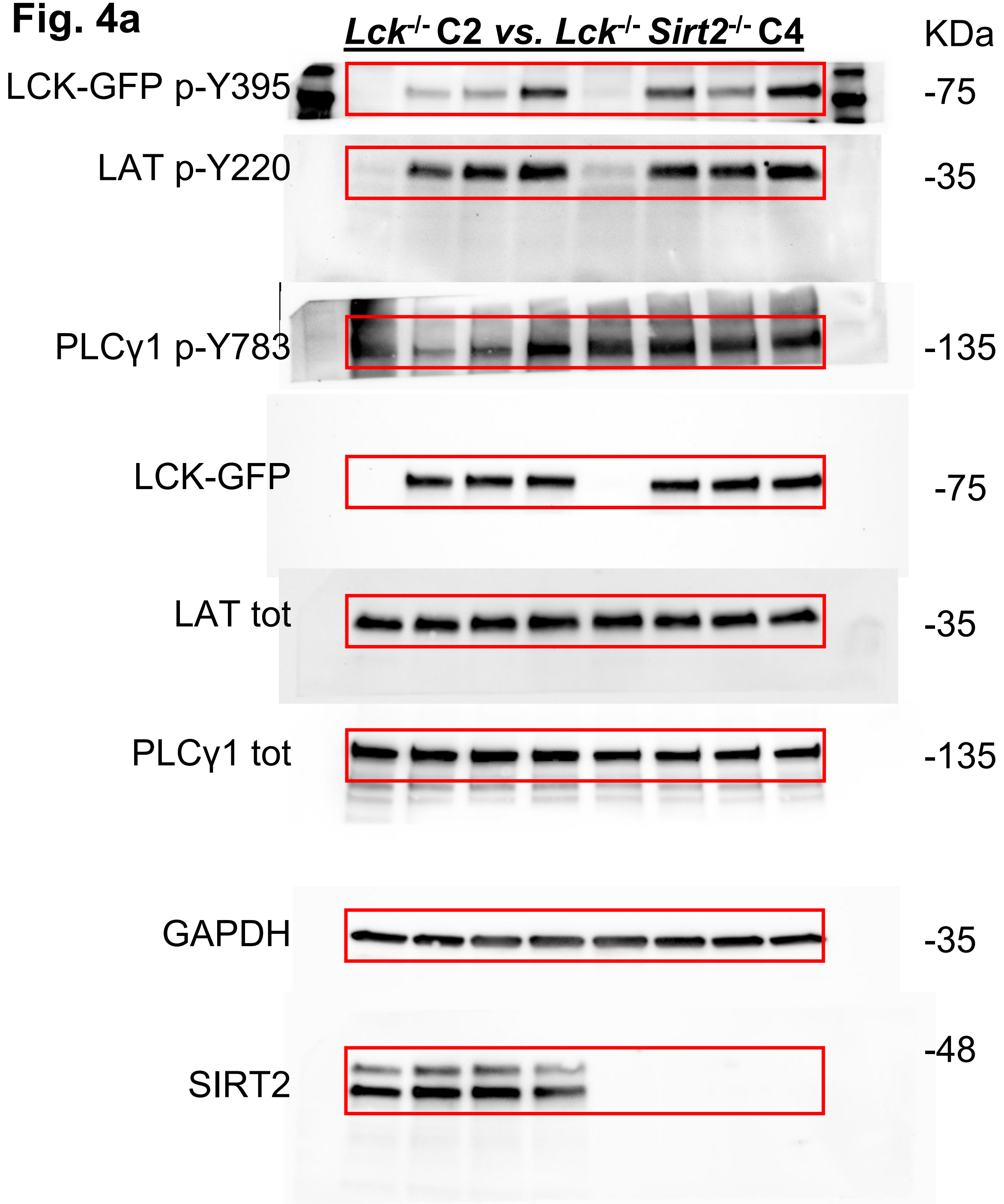

Extended Data Fig. 4a

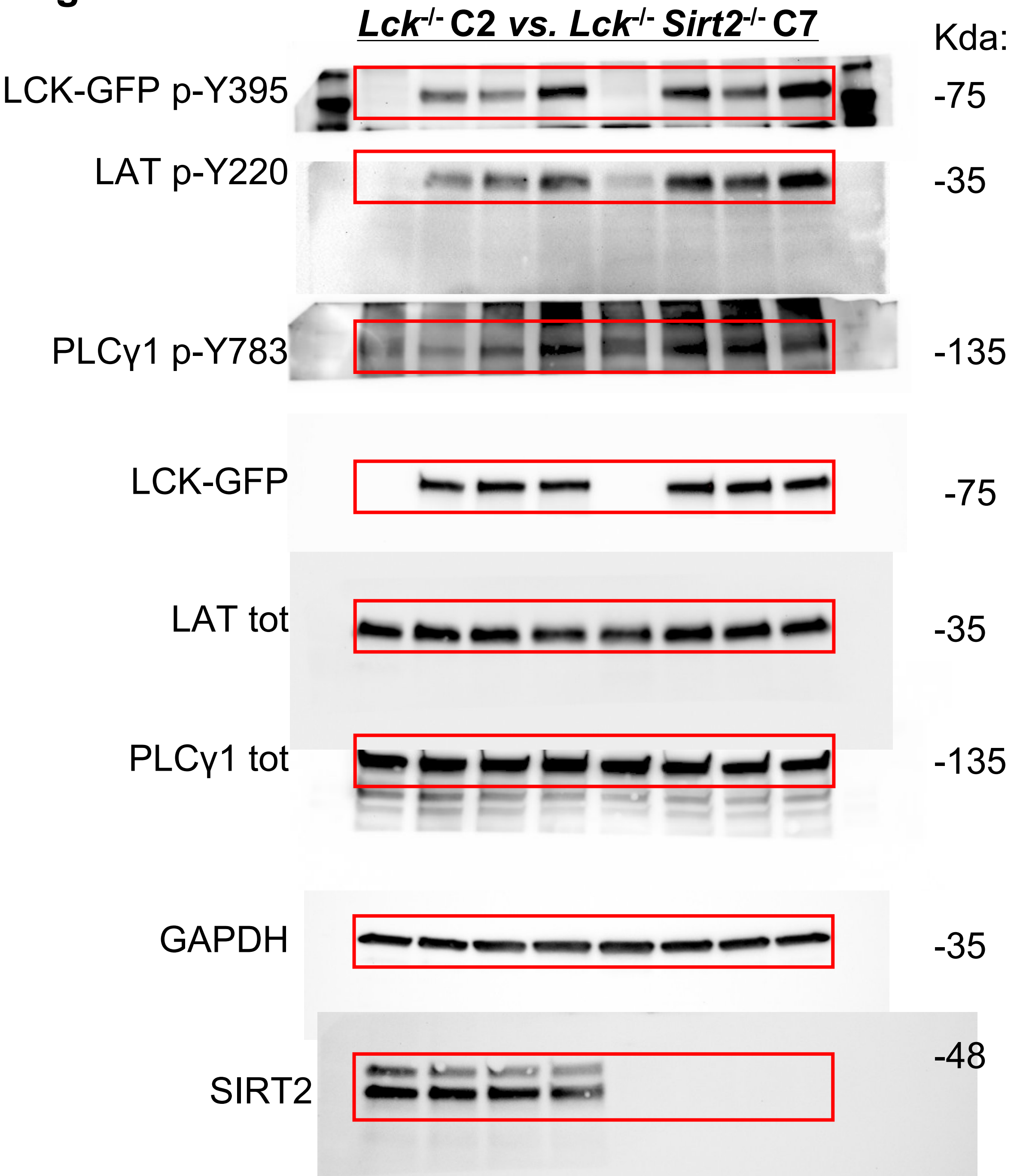

Extended Data Fig. 4a

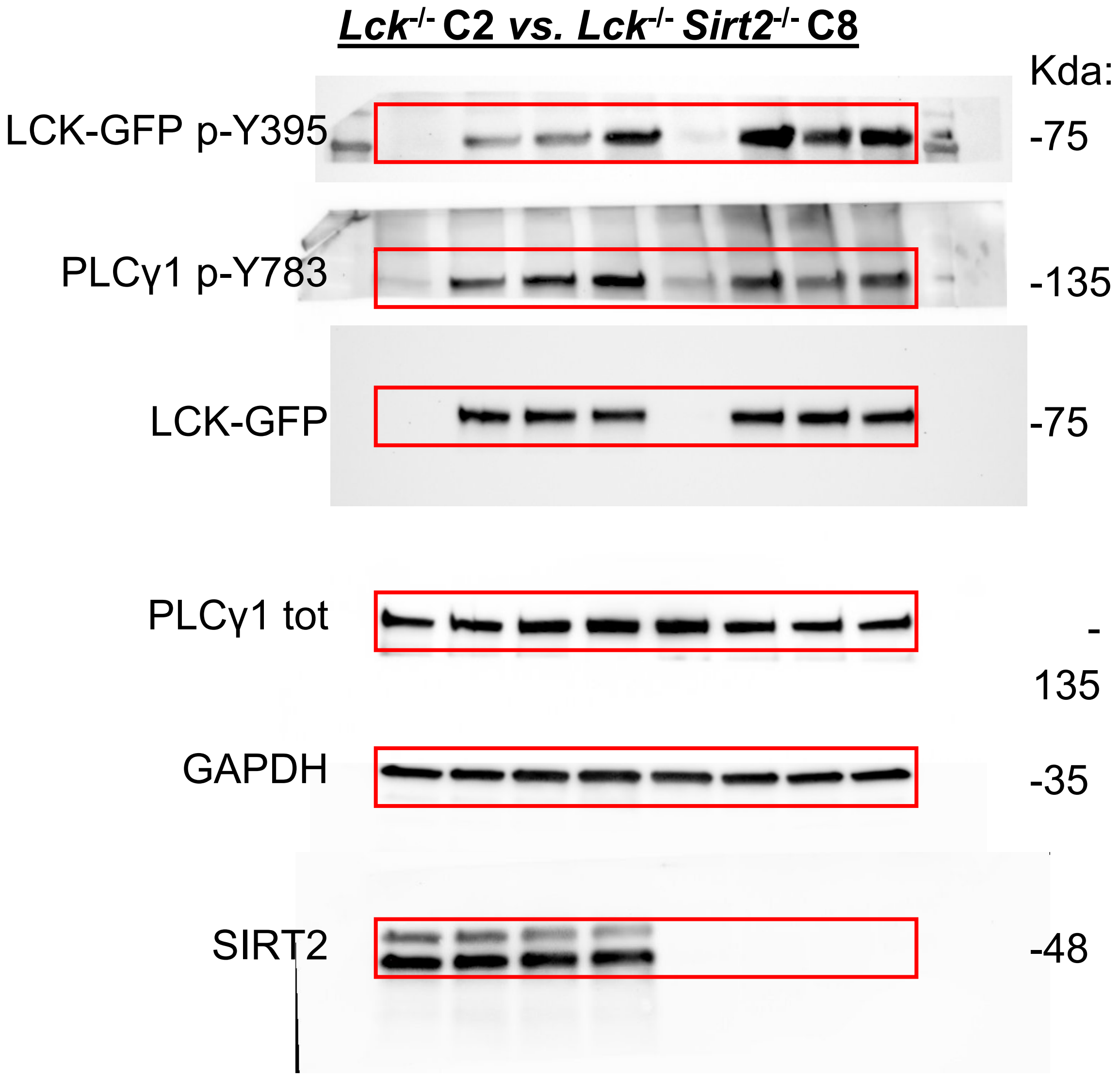

Extended Data Fig. 4a

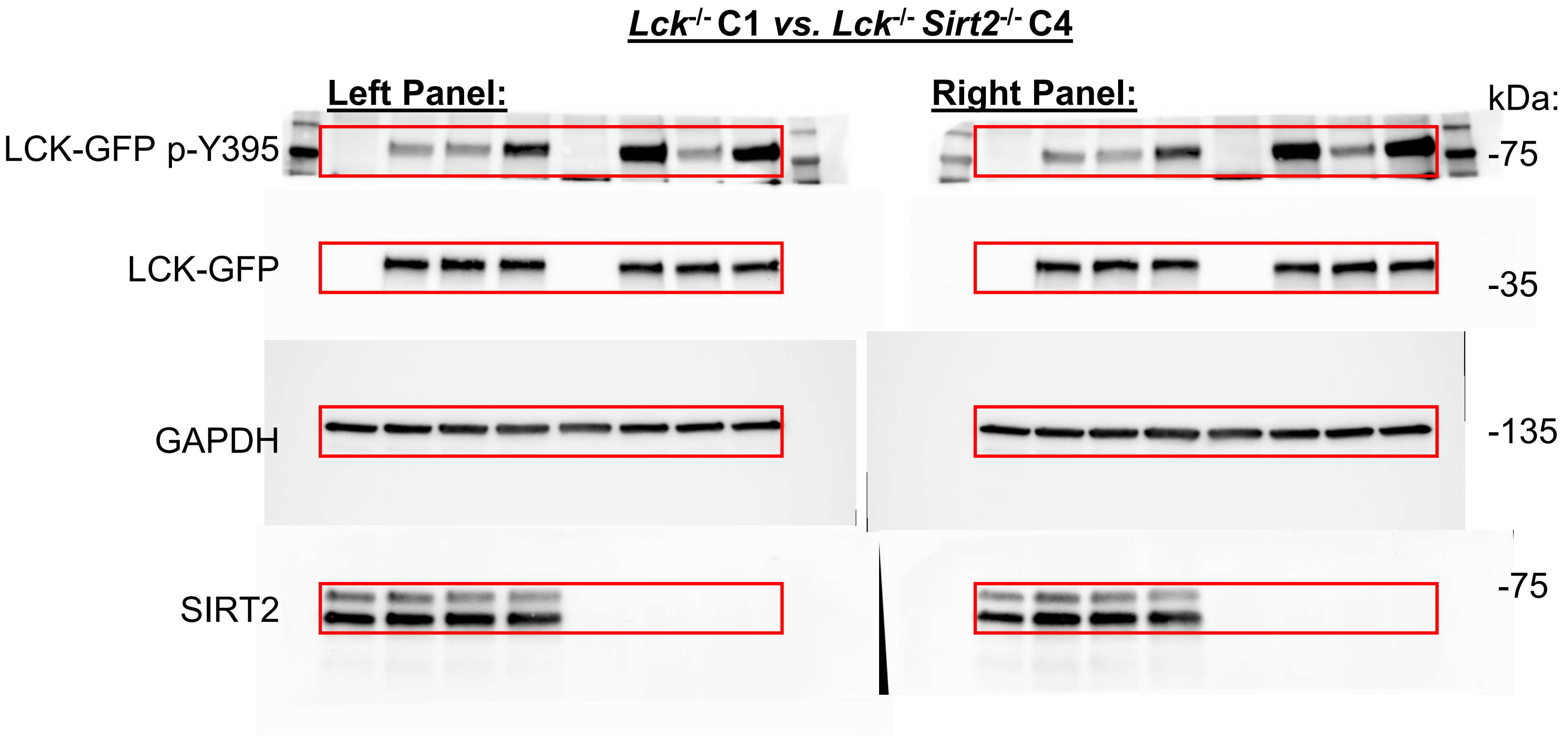

Extended Data Fig. 4a

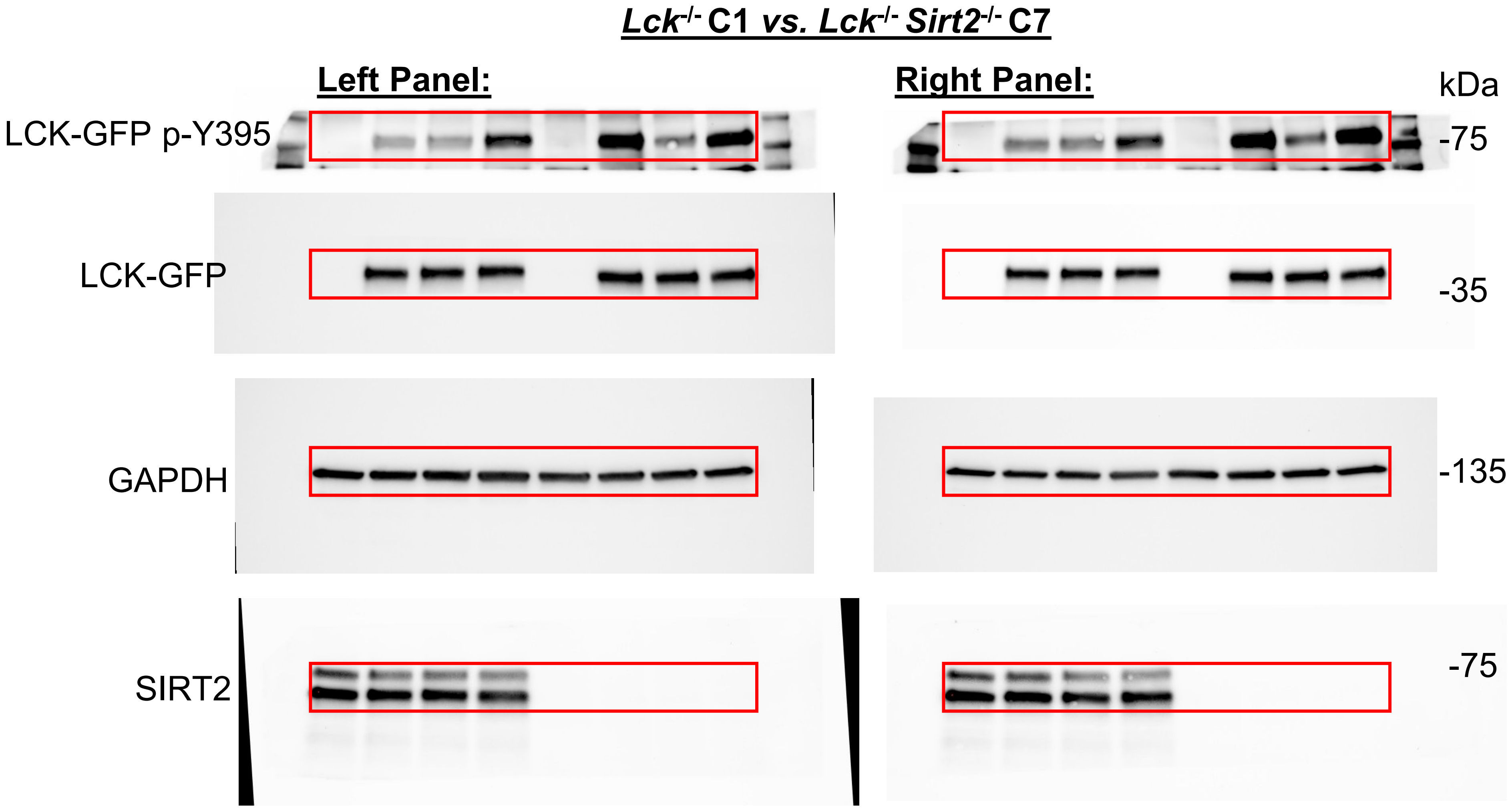

Extended Data Fig. 4b

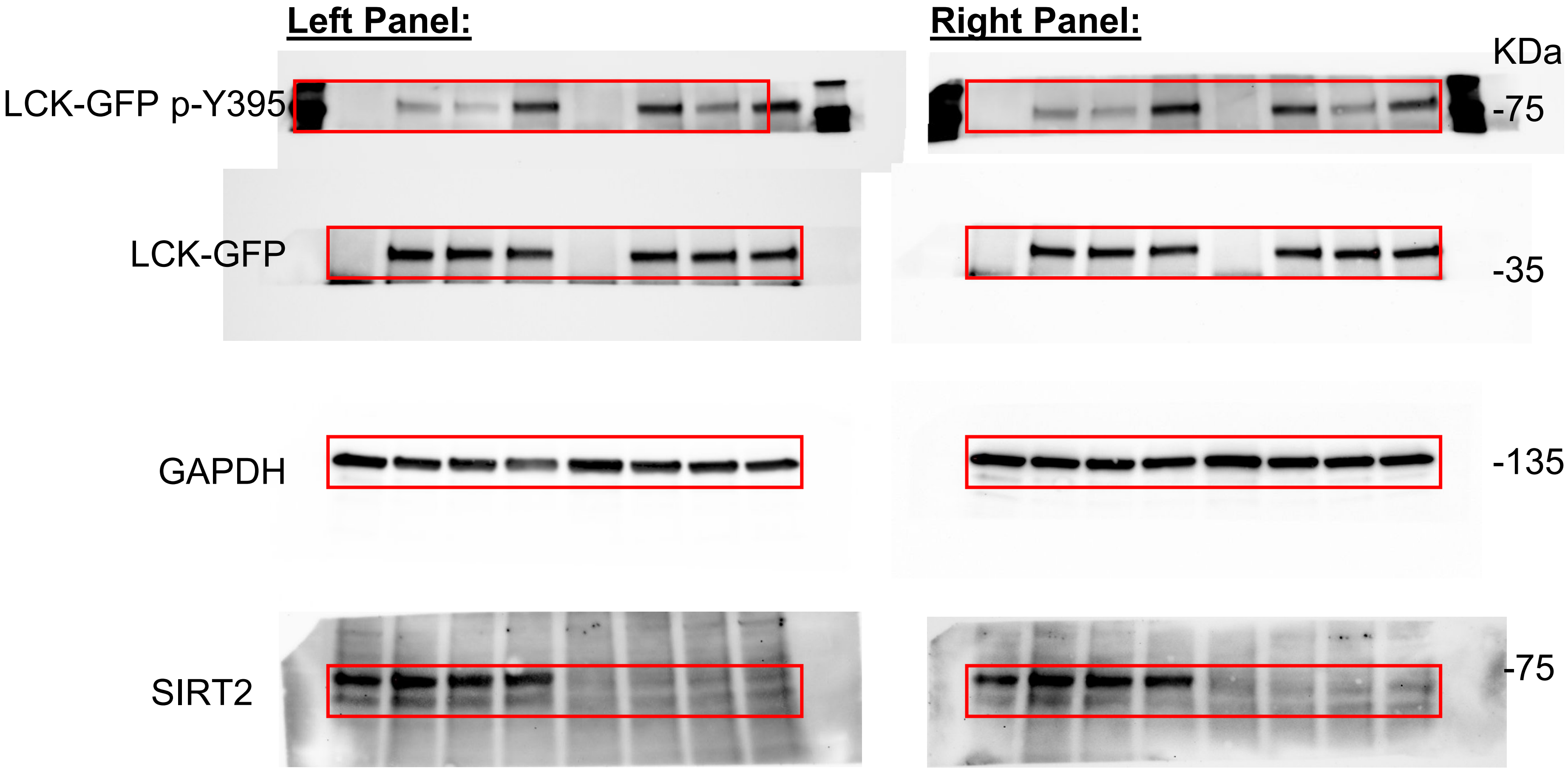

Extended Data Fig. 5b

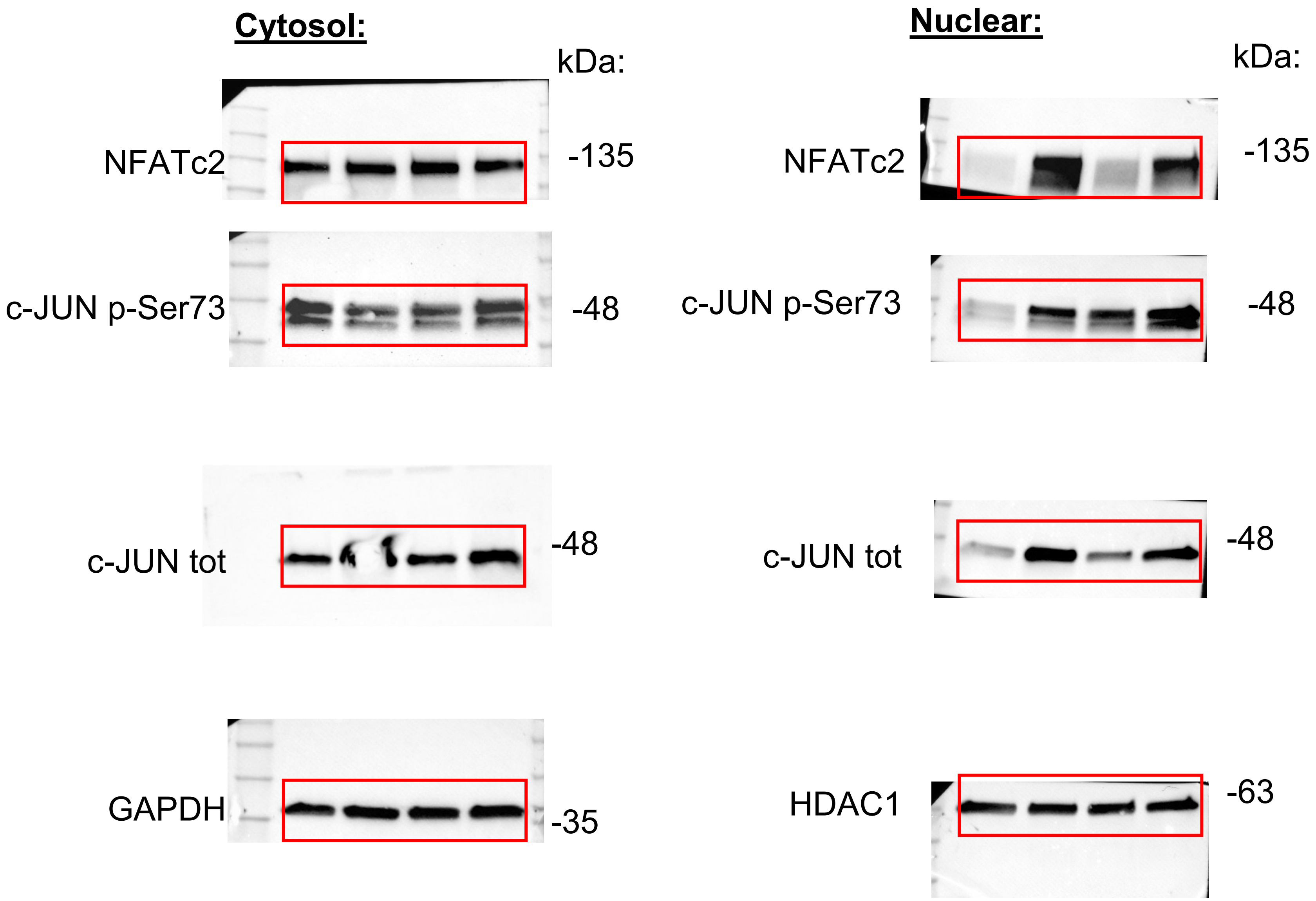

Extended Data Fig. 5c

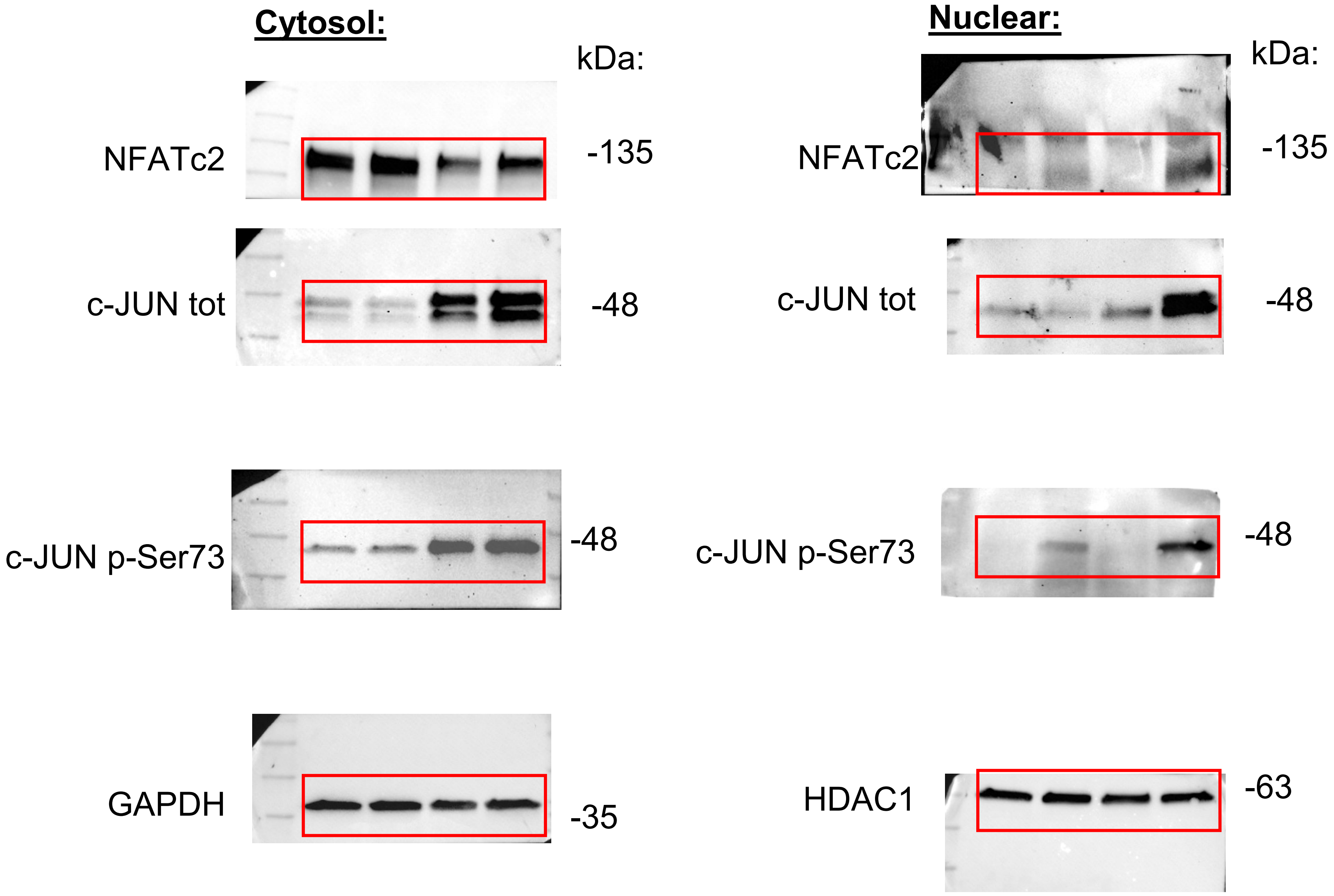

Extended Data Fig. 10f

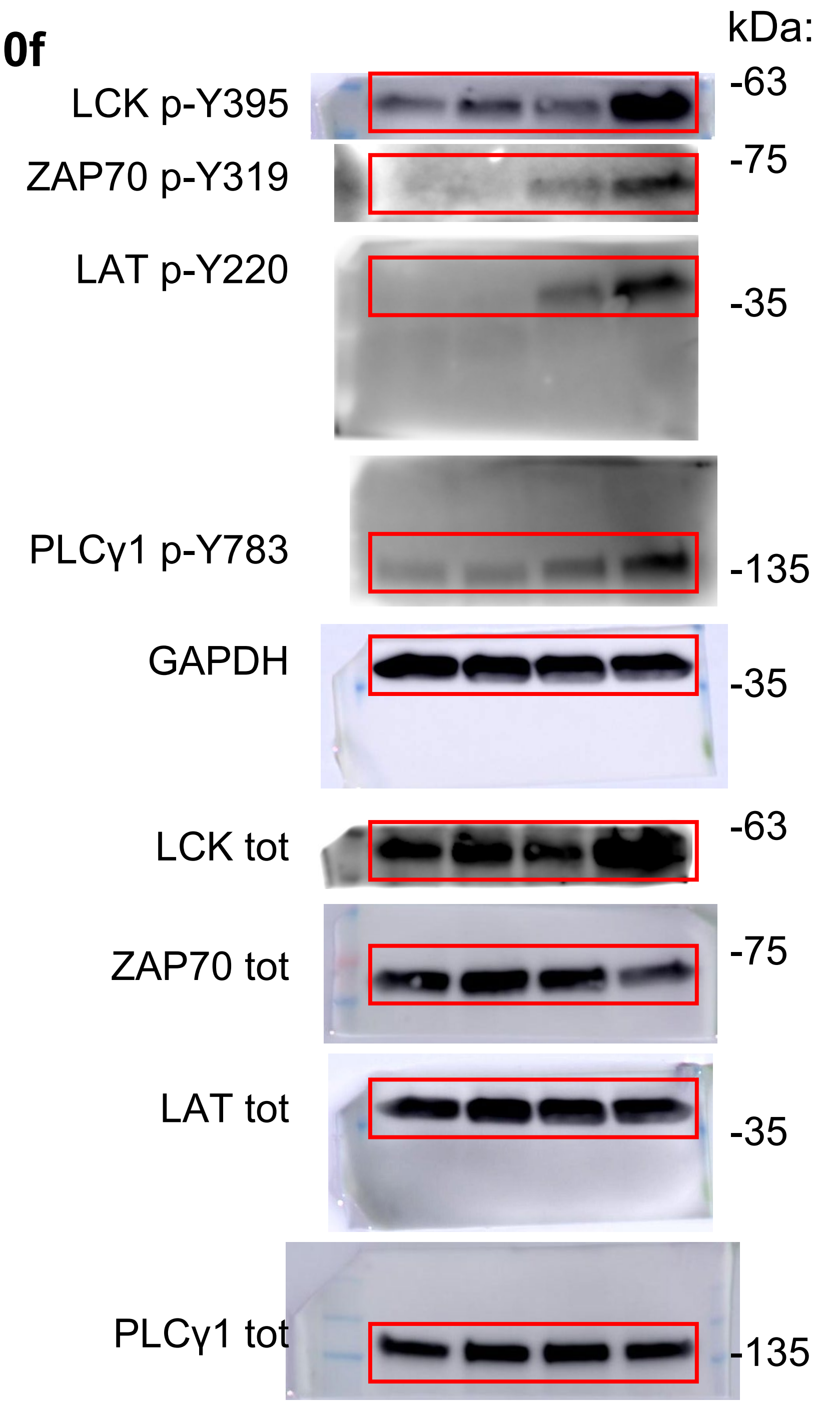

Extended Data Fig. 10g

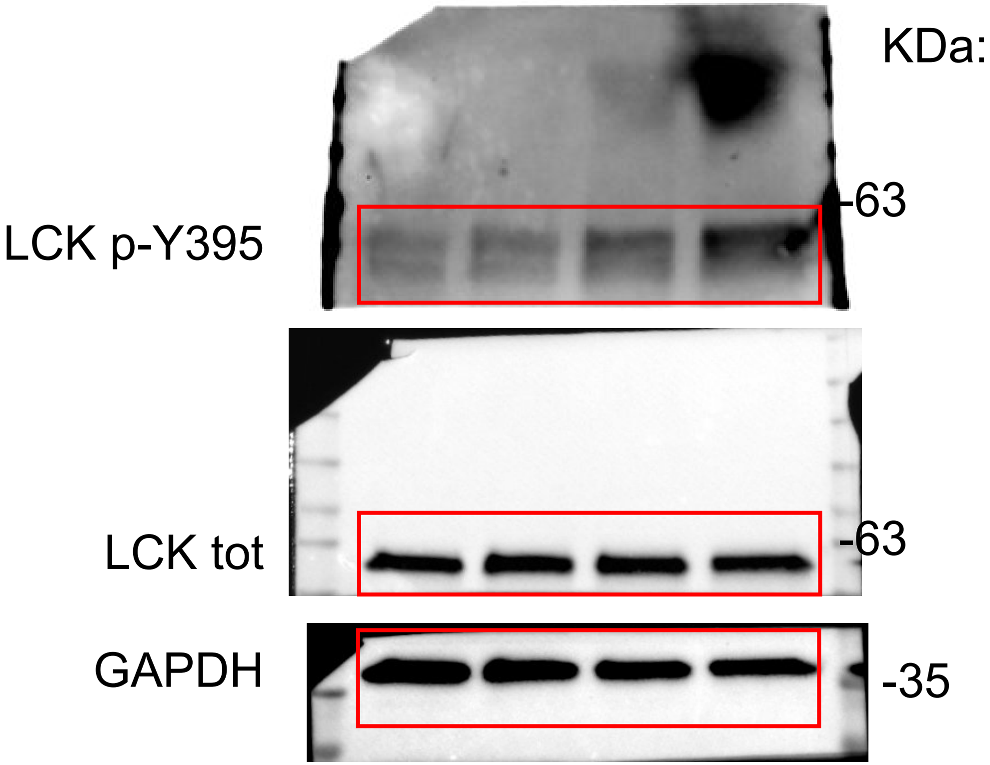

Extended Data Fig. 10h

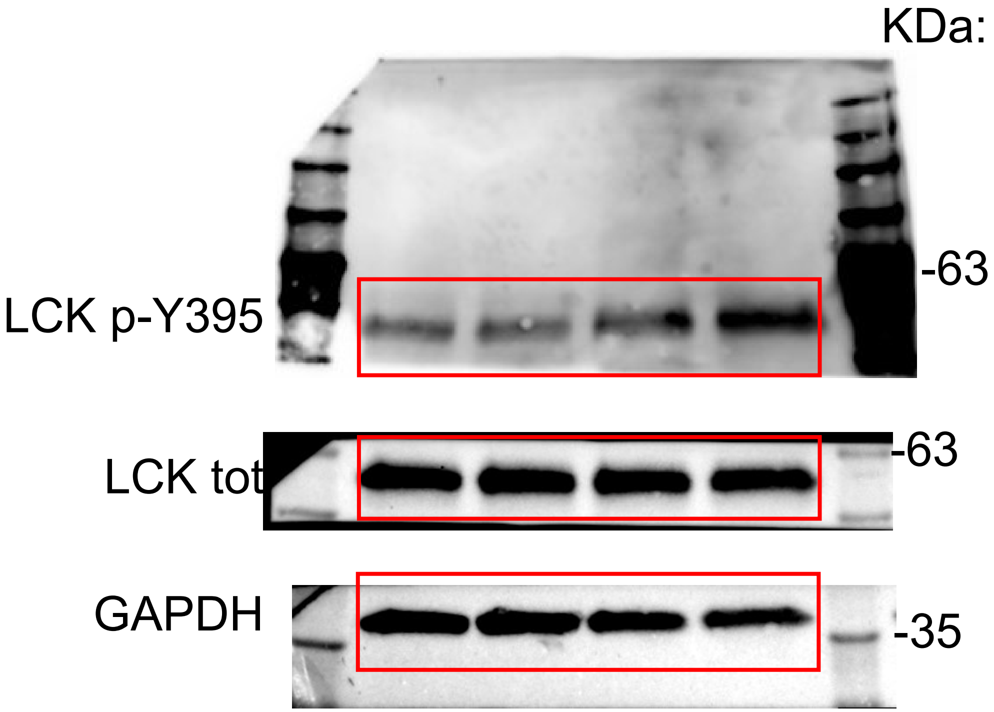

Extended Data Fig. 10i

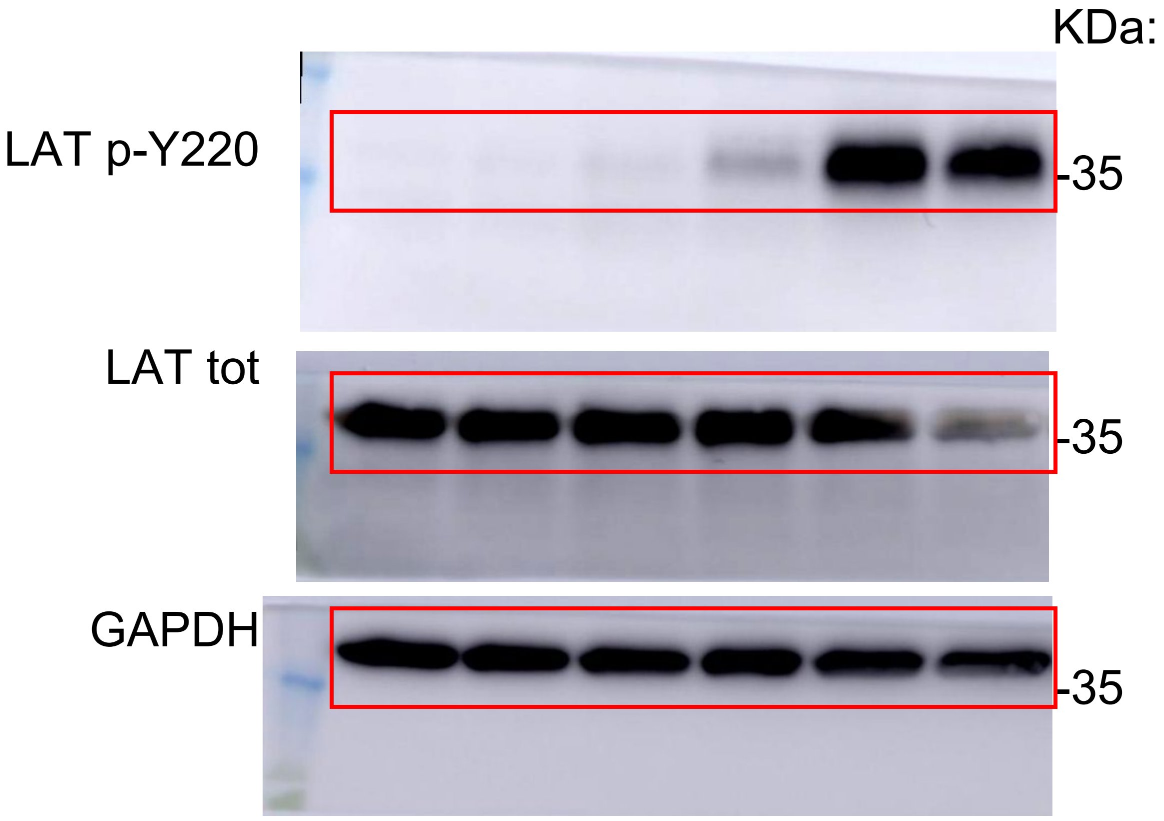

Extended Data Fig. 10j

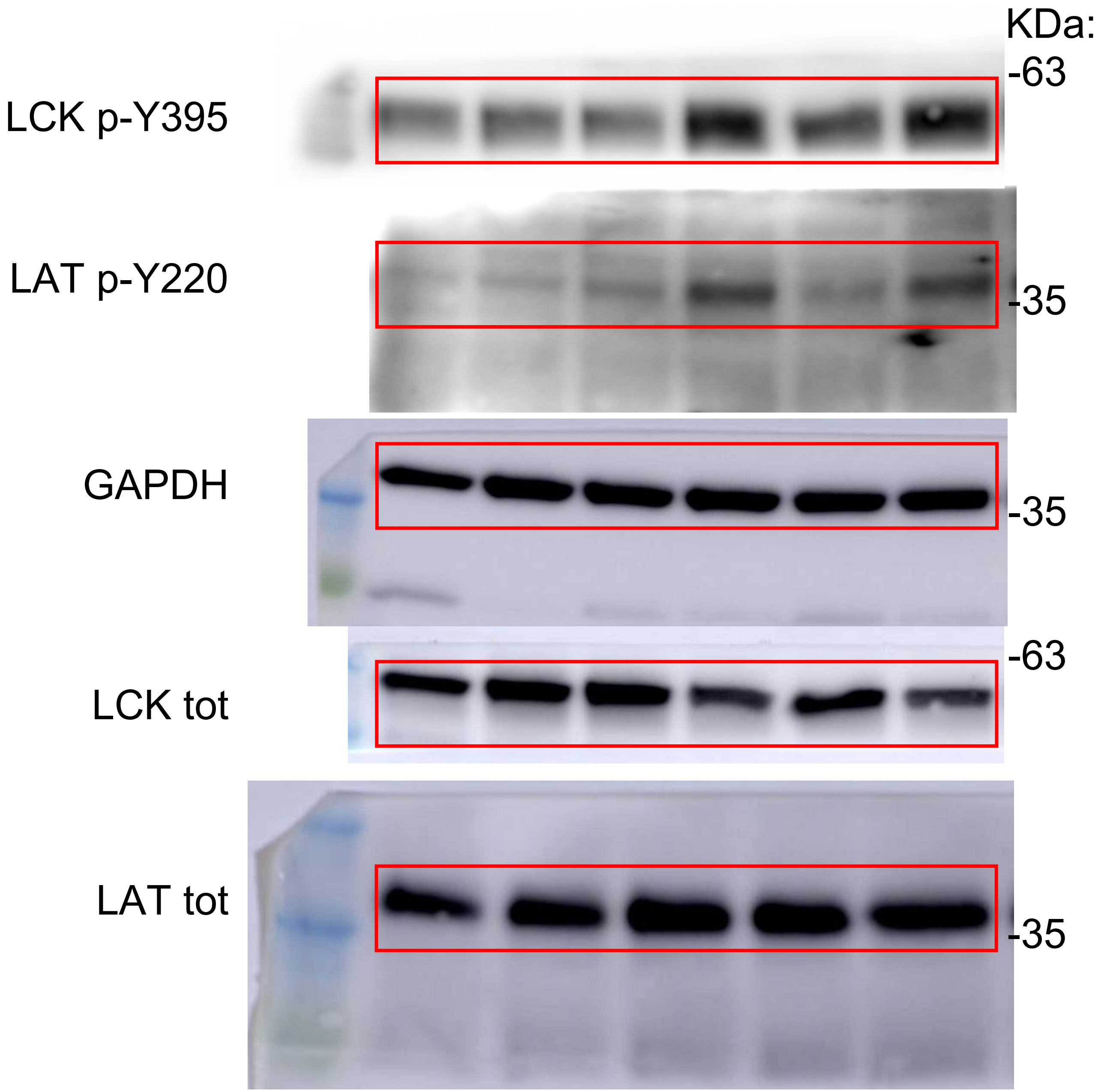

Extended Data Fig. 10k

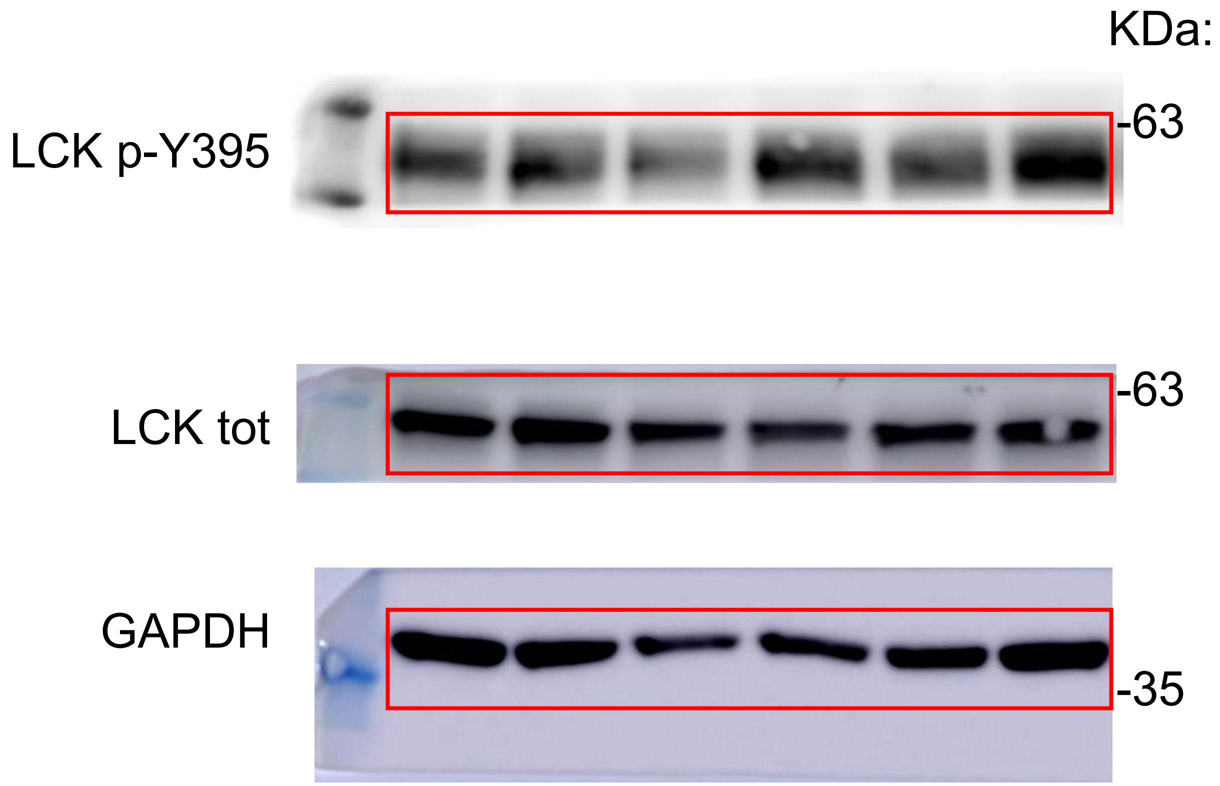

Supplement: Supplementary file 3 — Unprocessed immunoblots. [file 41590_2025_2377_MOESM3_ESM.pdf]
